# Discovery of Genetic Variation on Chromosome 5q22 Associated with Mortality in Heart Failure

**DOI:** 10.1371/journal.pgen.1006034

**Published:** 2016-05-05

**Authors:** J. Gustav Smith, Janine F. Felix, Alanna C. Morrison, Andreas Kalogeropoulos, Stella Trompet, Jemma B. Wilk, Olof Gidlöf, Xinchen Wang, Michael Morley, Michael Mendelson, Roby Joehanes, Symen Ligthart, Xiaoyin Shan, Joshua C. Bis, Ying A. Wang, Marketa Sjögren, Julius Ngwa, Jeffrey Brandimarto, David J. Stott, David Aguilar, Kenneth M. Rice, Howard D. Sesso, Serkalem Demissie, Brendan M. Buckley, Kent D. Taylor, Ian Ford, Chen Yao, Chunyu Liu, CHARGE-SCD consortium, EchoGen consortium, QT-IGC consortium, CHARGE-QRS consortium, Nona Sotoodehnia, Pim van der Harst, Bruno H. Ch. Stricker, Stephen B. Kritchevsky, Yongmei Liu, J. Michael Gaziano, Albert Hofman, Christine S. Moravec, André G. Uitterlinden, Manolis Kellis, Joyce B. van Meurs, Kenneth B. Margulies, Abbas Dehghan, Daniel Levy, Björn Olde, Bruce M. Psaty, L. Adrienne Cupples, J. Wouter Jukema, Luc Djousse, Oscar H. Franco, Eric Boerwinkle, Laurie A. Boyer, Christopher Newton-Cheh, Javed Butler, Ramachandran S. Vasan, Thomas P. Cappola, Nicholas L. Smith

**Affiliations:** 1 Department of Cardiology, Department of Clinical Sciences, Lund University, Lund, Sweden; 2 Department of Heart Failure and Valvular Disease, Skåne University Hospital, Lund, Sweden; 3 Program in Medical and Population Genetics, Broad Institute of Harvard and MIT, Cambridge, Massachusetts, United States of America; 4 Center for Human Genetic Research and Cardiovascular Research Center, Harvard Medical School and Massachusetts General Hospital, Boston, Massachusetts, United States of America; 5 Department of Clinical Sciences, Lund University, Malmö, Sweden; 6 Department of Epidemiology, Erasmus MC, University Medical Center Rotterdam, Rotterdam, the Netherlands; 7 Netherlands Consortium for Healthy Aging (NGI-NCHA), The Netherlands Genomics Initiative, Leiden, the Netherlands; 8 Human Genetics Center, University of Texas Health Science Center at Houston, Houston, Texas, United States of America; 9 Emory Clinical Cardiovascular Research Institute, Emory University, Atlanta, Georgia, United States of America; 10 Department of Cardiology, Leiden University Medical Center, Leiden, the Netherlands; 11 Department of Gerontology and Geriatrics, Leiden University Medical Center, Leiden, the Netherlands; 12 Brigham and Women's Hospital and Harvard Medical School, Boston, Massachusetts, United States of America; 13 Department of Biology, Massachusetts Institute of Technology, Cambridge, Massachusetts, United States of America; 14 Perelman School of Medicine, University of Pennsylvania, Philadelphia, Pennsylvania, United States of America; 15 The Framingham Heart Study, Framingham, Massachusetts, United States of America; 16 The Population Sciences Branch, National Heart, Lund and Blood Institute, Bethesda, Maryland, United States of America; 17 Department of Cardiology, Boston Children's Hospital, Boston, Massachusetts, United States of America; 18 Department of Epidemiology, University of Washington, Seattle, Washington, United States of America; 19 Novartis Institutes for BioMedical Research, Cambridge, Massachusetts, United States of America; 20 Department of Biostatistics, Boston University School of Public Health, Boston, Massachusetts, United States of America; 21 Academic Section of Geriatric Medicine, Institute of Cardiovascular and Medical Sciences, Faculty of Medicine, University of Glasgow, Glasgow, United Kingdom; 22 Baylor College of Medicine, Houston, Texas, United States of America; 23 Department of Biostatistics, University of Washington, Seattle, Washington, United States of America; 24 Department of Pharmacology and Therapeutics, University College Cork, Cork, Ireland; 25 Institute for Translational Genomics and Population Sciences, Los Angeles Biomedical Research Institute and Department of Pediatrics, Harbor-UCLA Medical Center, Torrance, California, United States of America; 26 Robertson Center for Biostatistics, University of Glasgow, Glasgow, United Kingdom; 27 Cardiovascular Health Research Unit, Department of Medicine, University of Washington, Seattle, Washington, United States of America; 28 Department of Cardiology, University of Groningen, University Medical Center Groningen, Groningen, the Netherlands; 29 Department of Internal Medicine, Erasmus MC, University Medical Center Rotterdam, Rotterdam, the Netherlands; 30 Inspectorate for Health Care, The Hague, the Netherlands; 31 Department of Medical Informatics, Erasmus MC, University Medical Center Rotterdam, Rotterdam, the Netherlands; 32 Department of Internal Medicine, Section on Geronotology and Geriatric Medicine, Wake Forest School of Medicine, Winston-Salem, North Carolina, United States of America; 33 Department of Epidemiology and Prevention, Division of Public Health Sciences, Wake Forest University Health Sciences, Winston-Salem, North Carolina, United States of America; 34 Department of Cardiovascular Medicine, Cleveland Clinic Foundation, Cleveland, Ohio, United States of America; 35 Department of Health Services, University of Washington, Seattle, Washington, United States of America; 36 Group Health Research Institute, Group Health Cooperative, Seattle, Washington, United States of America; 37 Durrer Center for Cardiogenetic Research, Amsterdam, the Netherlands; 38 Interuniversity Cardiology Institute of the Netherlands, Utrecht, the Netherlands; 39 Human Genome Sequencing Center, Baylor College of Medicine, Houston, Texas, United States of America; 40 Departments of Medicine and Preventive Medicine, Boston University School of Medicine, Boston, Massachusetts, United States of America; 41 Seattle Epidemiologic Research and Information Center, Department of Veteran Affairs Office of Research and Development, Seattle, Washington, United States of America; Institute for Molecular Medicine Finland (FIMM), FINLAND

## Abstract

Failure of the human heart to maintain sufficient output of blood for the demands of the body, heart failure, is a common condition with high mortality even with modern therapeutic alternatives. To identify molecular determinants of mortality in patients with new-onset heart failure, we performed a meta-analysis of genome-wide association studies and follow-up genotyping in independent populations. We identified and replicated an association for a genetic variant on chromosome 5q22 with 36% increased risk of death in subjects with heart failure (rs9885413, *P* = 2.7x10^-9^). We provide evidence from reporter gene assays, computational predictions and epigenomic marks that this polymorphism increases activity of an enhancer region active in multiple human tissues. The polymorphism was further reproducibly associated with a DNA methylation signature in whole blood (*P* = 4.5x10^-40^) that also associated with allergic sensitization and expression in blood of the cytokine *TSLP* (*P* = 1.1x10^-4^). Knockdown of the transcription factor predicted to bind the enhancer region (NHLH1) in a human cell line (HEK293) expressing NHLH1 resulted in lower *TSLP* expression. In addition, we observed evidence of recent positive selection acting on the risk allele in populations of African descent. Our findings provide novel genetic leads to factors that influence mortality in patients with heart failure.

## Author Summary

In this study, we applied a genome-wide mapping approach to study molecular determinants of mortality in subjects with heart failure. We identified a genetic variant on chromosome 5q22 that was associated with mortality in this group and observed that this variant conferred increased function of an enhancer region active in multiple tissues. We further observed association of the genetic variant with a DNA methylation signature in blood that in turn is associated with allergy and expression of the gene *TSLP* (Thymic stromal lymphoprotein) in blood. Knockdown of the transcription factor predicted to bind the enhancer region also resulted in lower *TSLP* expression. The *TSLP* gene encodes a cytokine that induces release of T-cell attracting chemokines from monocytes, promotes T helper type 2 cell responses, enhances maturation of dendritic cells and activates mast cells. Development of *TSLP* inhibiting therapeutics are underway and currently in phase III clinical trials for asthma and allergy. These findings provide novel genetic leads to factors that influence mortality in patients with heart failure and in the longer term may result in novel therapies.

## Introduction

Heart failure (HF) is a common clinical condition in which the heart fails to maintain blood circulation adequate to meet the metabolic demands of the body without increased cardiac filling pressures. HF is the result of chronic ventricular remodelling initiated by myocardial injury, volume/pressure overload, or intrinsic cardiomyopathic processes. Progression of HF is a complex process involving many tissues, driven by activation of neurohormonal pathways, which induce gradual myocardial hypertrophy, ventricular dilation, and deterioration of cardiac function, often resulting in death from low cardiac output, arrhythmia, or thromboembolic complications [1]. Activation of such neurohormonal pathways in the short term increases cardiac output when necessary. However, long-term activation results in accelerated ventricular remodelling and myocyte death. Inhibitors of deleterious neurohormonal pathways, including adrenergic [2–4] and renin-angiotensin-aldosterone (RAAS) [5–8] pathways have been shown to improve ventricular function and survival in patients with HF and are the mainstay of current pharmacological treatment of HF [9–10]. Despite advances in therapy with neurohormonal antagonists, mortality after onset of HF remains high [9–13] and continued progress to identify additional therapeutic targets is needed.

Genome-wide association (GWA) studies have the potential to identify in an agnostic manner genetic variants related to clinical outcomes in humans and has led to the identification of novel pathways [14] and potential treatments [15] for cardiovascular traits. Heritable factors have been shown to be predictive of mortality in certain heart failure patients [16]. We therefore implemented a genome-wide association approach to identify novel molecular determinants of mortality in patients with new-onset HF.

## Results

### Two-stage GWA study

We expanded our previously published GWA study [17] of HF mortality with additional samples and extended follow-up in Stage 1. Stage 1 included 2,828 new-onset HF patients from five community-based cohorts, thus representative of the general population of HF patients, as part of the Cohorts for Heart and Aging Research in Genomic Epidemiology (CHARGE) consortium [18]: the Atherosclerosis Risk in Communities (ARIC) Study, the Cardiovascular Health Study (CHS), the Framingham Heart Study (FHS), the Health, Aging and Body Composition (Health ABC) Study, and the Rotterdam Study (RS). Cohorts are described in detail in **S1 Text**. HF was defined using international published criteria as outlined in **S1 Table**. Subjects in Stage 1 cohorts were of European ancestry, predominantly male, and approximately 20–30% had a history of myocardial infarction at the time of HF diagnosis. Additional characteristics are shown in **Table 1**. During an average follow-up time of 3.5 years, 1,798 deaths occurred. The sample-size weighted average 1-year mortality rate was 28%. Among deaths, 51% were classified as cardiovascular, 19% were due to neoplasms, 10% were respiratory deaths, and the remaining were due to other miscellaneous causes. Genotyping using high-density Illumina or Affymetrix single nucleotide polymorphism (SNP) arrays, followed by imputation to the HapMap CEU release 22 imputation panel was performed in each cohort. Population stratification was assessed and corrected in each cohort as described in **S1 Text**. Association with time to death following HF diagnosis was examined in each cohort using Cox proportional hazards models with censoring at loss to follow-up. Mild inflation of test statistics was observed only in the Framingham Heart Study (FHS) as shown in **S1 Fig** (λ_GC_ = 1.07, other cohorts ≤ 1.03), and genomic control was applied in each individual study. In the meta-analysis of all cohorts, there was no evidence of inflated test statistics overall (λ_GC_ = 1.00) as shown in **S2 Fig**, so no further genomic control was needed. Results for all SNPs across the genome are plotted in **S3 Fig**. Single nucleotide polymorphisms (SNPs) passing a significance threshold specified *a priori* as *P* < 5.0x10^-7^, as used in our previous article [17], were carried forward to a second stage of genotyping in independent cohorts.

**Table 1:** Characteristics of cohorts in stage 1.

	ARIC	ARIC2	CHS	FHS	Health ABC	RS	RS2
**HF sample size**	691	84	838	249	173	748	45
**Age at HF** (years)	67.3 (6.6)	68.8 (6.5)	82.4 (6.1)	80.7 (9.6)	79.1 (3.8)	79.6 (8.0)	73.7 (8.6)
**Male sex** (%)	60.1	58.3	56.3	47.8	61.8	48.8	68.9
**Body mass index** (kg/m^2^)	29.5 (5.9)	28.7 (6.3)	27.0 (4.6)	28.5 (5.4)	26.5 (4.3)	27.2 (4.1)	27.1 (4.0)
**Diabetes** (%)	28.7	29.8	18.1	17.7	20.8	8.6	11.1
**Hypertension** (%)	58.5	53.7	52.3	76.3	79.8	81.0	73.3
**Current smoking (%)**	28.6	33.3	8.4	10.4	7.5	18.6	26.7
**History of MI** (%)	25.2	26.2	5.4	27.7	24.9	28.1	28.9
**Follow-up time** (years)	3.9 (3.9)	4.1 (4.0)	3.5 (2.4)	1.8 (1.9)	3.0 (2.7)	3.9 (3.7)	3.6 (2.6)
**Mortality rate, 1 year**	0.25	0.22	0.28	0.34	0.27	0.30	0.25
**All-cause mortality** (n, %)	327 (47.3)	42 (50.0)	606 (72.3)	156 (62.7)	87 (50.3)	556 (74)	24 (53)

Age, body mass index and follow-up time are presented as mean (standard deviation). Categorical variables are presented as percentages. Body mass index, diabetes, hypertension and smoking refer to the nearest study exam prior to heart failure diagnosis whereas age, follow-up time, mortality rate and all-cause death refer to the time of HF diagnosis. Mortality rate refers to the 1-year Kaplan-Meier estimate, with censoring at end of or loss to follow-up. The prevalence of myocardial infarction in CHS is low, because this was an exclusion criterion at the study baseline. MI, Myocardial infarction.

Five SNPs on chromosome 5q22 and one SNP on chromosome 3p22 passed the pre-specified *P*-value threshold. Results for all six SNPs are shown in **Table 2** and **S3 Table**. The five SNPs on chromosome 5q22 were highly correlated (pairwise r^2^ > 0.9). Two sentinel SNPs, rs9885413 and rs12638540, on chromosomes 5q22 and 3p22, respectively, were next genotyped in 1,870 European-ancestry subjects with new-onset HF from four independent cohorts in Stage 2: Malmö Diet and Cancer, Malmö Preventive Project, Physicians’ Health Study, and the PROSPER trial. Characteristics of populations in Stage 2 are shown in **S2 Table**. During an average sample-size weighted follow-up of 4.3 years in Stage 2 samples, 889 patients died. We observed evidence of association with mortality for rs9885413 on chromosome 5q22 (*P* = 0.006) but not for the SNP rs12638540 (*P* = 0.18) which reached nominal significance in our previous analysis [17]. Results for both SNPs are shown in **Table 2**. In the combined results from Stages 1 and 2, rs9885413 was associated with a 36% relative increase in mortality per minor allele (*P* = 2.7x10^-9^). There was no evidence for effect heterogeneity across cohorts in the two stages (*P* for heterogeneity = 0.39) as shown in **S4 Table**. The SNP had a similar minor allele frequency (MAF = 0.07) across cohorts. Information on cause-specific mortality was available from death certificates in a subset of cohorts (**S5 Table**) and was explored descriptively due well-known problems with substantial misclassification in death certificate data and low power for agnostic GWAS of individual causes. The minor allele frequency was slightly higher for several causes of death associated with heart failure, including renal, pulmonary and endocrine mortality and death from ischemic heart disease.

**Table 2:** Association of genetic polymorphisms with HF mortality.

SNP				Stage 1		Stage 2		Combined		Heterogeneity	
	*Chr*	*Alleles*	*MAF*	*HR (95% CI)*	*P*	*HR (95% CI)*	*P*	*HR (95% CI)*	*P*	*I*^*2*^	*P*
rs12638540	3p22	G / A	0.04	1.50 (1.28–1.75)	4x10^-7^	1.10 (0.89–1.37)	0.18	1.35 (1.19–1.54)	3.08x10^-6^	0	0.53
rs9885413	5q22	T / G	0.07	1.40 (1.23–1.58)	1x10^-7^	1.25 (1.05–1.49)	0.006	1.36 (1.23–1.51)	2.65x10^-9^	0.15	0.39
rs12658193	5q22	T / G	0.07	1.39 (1.23–1.58)	2x10^-7^	-		-		-	-
rs11956079	5q22	T / C	0.07	1.39 (1.23–1.58)	2x10^-7^	-		-		-	-
rs10069077	5q22	T / C	0.07	1.39 (1.23–1.58)	2x10^-7^	-		-		-	-
rs10068260	5q22	T / A	0.06	1.42 (1.24–1.62)	2x10^-7^	-		-		-	-

Results for SNPs with *P*-value < 5x10^-7^ in stage 1 are presented with minor/major alleles, minor allele frequency (MAF) and effect estimate (HR, hazard ratio) with 95% confidence intervals (95% CI) per minor allele from multivariable models adjusting for sex and age at the time of diagnosis. The index SNP at each locus was carried forward to stage 2. Effect heterogeneity was evaluated across the combined stage 1 and 2 samples. Chr, Chromosome.

### Lack of association with myocardial traits

We next examined whether rs9885413 on chromosome 5q22 that was associated with HF mortality was also associated with differences in myocardial structure and function, which could potentially mediate the association (**S6 Table**). In 12,612 individuals from the EchoGen Consortium [19], the SNP was not associated with major echocardiographic characteristics. The SNP rs9885413 was not associated with incident HF in 20,926 individuals from the general population in the CHARGE-HF study [20], or with cardiac endocrine function, as determined by plasma levels of atrial and B-type natriuretic peptides (all *P* > 0.05), in a GWA study of 5,453 individuals from the population-based Malmö Diet and Cancer study [21]. No association was observed with electrocardiographic measures of cardiac conduction (n = 39,222) [22] or repolarization (n = 74,149) [23], which confer risk of ventricular arrhythmia, or with sudden cardiac death in 4,496 sudden death cases and over 25,000 controls from the general population (described in **S1 Text**).

### Functional enhancer annotation

**Fig 1:**
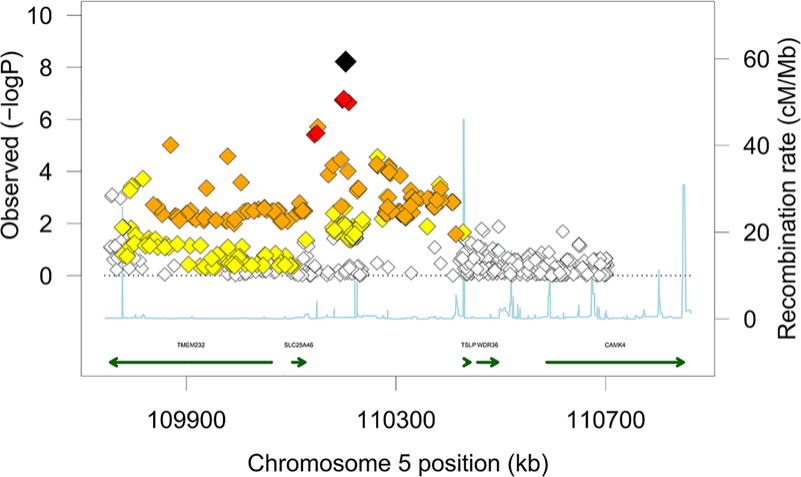
Regional association plot for HF mortality at the 5q22 locus. The plot covers the genomic region from 450 kb upstream of the SNP to 650 kb downstream. Diamonds represent SNPs. The large black diamond represents the SNP with the lowest *P*-value (rs9885413), with the *P*-value from the combined meta-analysis presented. Diamond color represents strength of pairwise correlation with the strongest SNP. Red represents r^2^ ≥ 0.8, orange represents r^2^ 0.5–0.8, yellow represents r^2^ 0.2–0.5, and white represents r^2^ < 0.2. Recombination rate is plotted in the background and known genes are represented in the bottom of the plot. Positions refer to NCBI build 36. SNP correlations and recombination rates were obtained from HapMap release 22.

The lead SNP rs9885413 on chromosome 5q22 that was associated with mortality is located in an intergenic region, 100 kb downstream of the gene *SLC25A46*, 114 kb upstream of *TMEM232*, and 230 kb upstream of *TSLP* as shown in **Fig 1**. The SNP is not in linkage disequilibrium with any known coding SNP in the 1000 Genomes Project database (no coding SNP with r^2^ > 0.01 to the sentinel SNP). We therefore sought to evaluate gene regulatory functions of this SNP.

**Fig 2:**
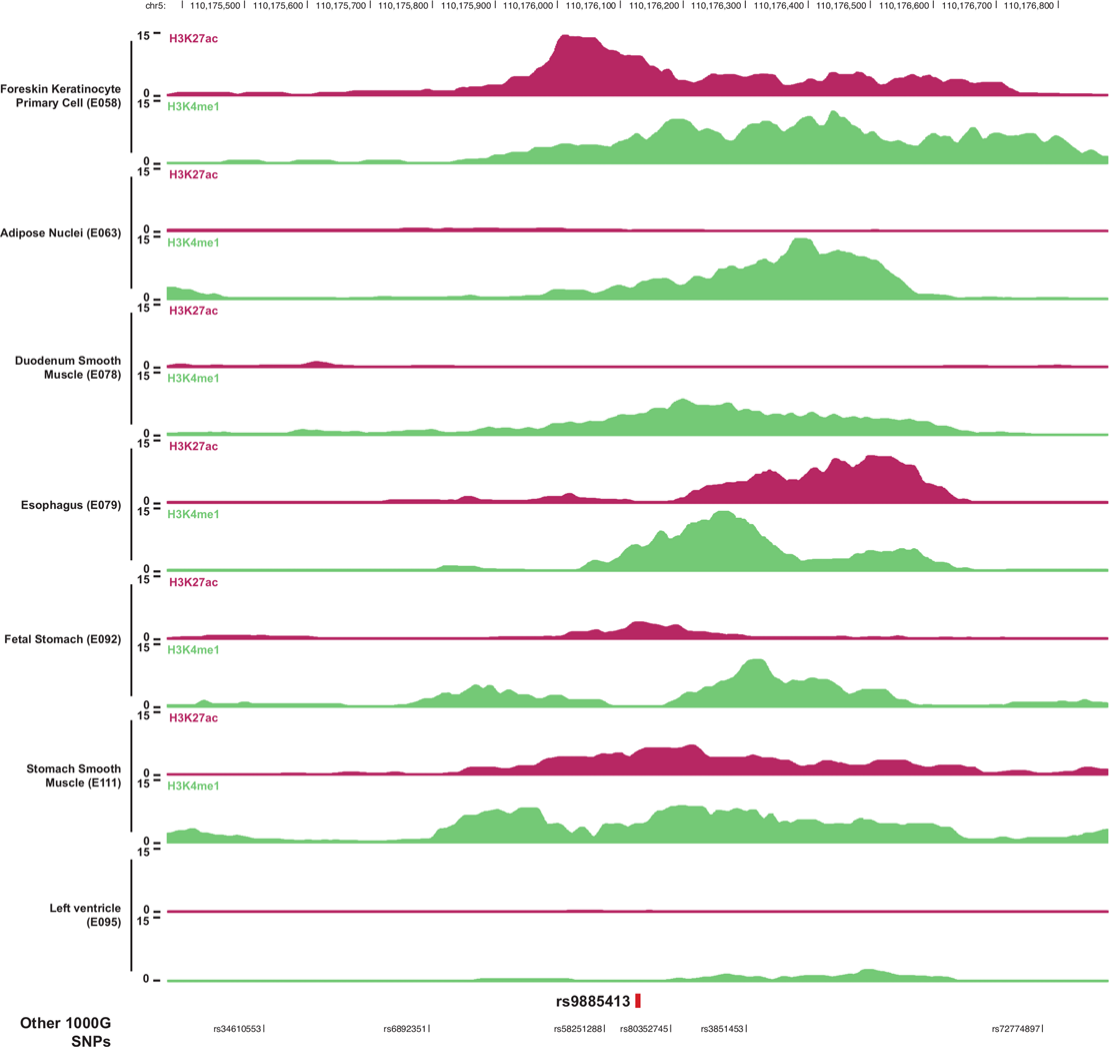
Epigenomic marks indicating enhancer activity at the 5q22 locus. Histone marks in samples from the ROADMAP Epigenomics Project at the enhancer overlapping the lead SNP rs9885413 in heart tissue (left ventricle) and in tissues with evidence of an active enhancer at the locus. Histone marks are monomethylation (H3K4Me1) of the fourth residue (lysine) and acetylation of the 27th residue (H3K27Ac) of histone H3. Positions refer to NCBI build 36.

In 129 human tissues from the ROADMAP Epigenomics project [24], we studied whether rs9885413 or strongly correlated SNPs (a total of 9 at r^2^ > 0.8) are located in regulatory regions, as determined by histone modification patterns. None of the 9 SNPs was located in an active regulatory region in cardiac tissues (**S7 Table**), but rs9885413 was located in a predicted enhancer in several epithelial or mesenchymal tissues, including keratinocytes, gastrointestinal cell types and adipose cells (**Fig 2** and **S7 Table**). Regulatory motif annotations in HaploReg indicate that the SNP causes a change in a regulatory motif predicted to bind the transcription factor NHLH1 as shown in **S8 Table**. Interestingly, NHLH1-null mice have been shown to be predisposed to premature, adult-onset unexpected death in the absence of signs of cardiac structural or conduction abnormalities, in particular when mice were exposed to stress [25]. Little is known about the function of NHLH1, but it is widely expressed in human tissues and has been shown to regulate expression of key inflammatory genes [26].

To experimentally test the effect of rs9885413 on enhancer activity, the 100 bp region flanking the SNP (50 bp on either side) was cloned into a reporter vector and transfected into HEK293 cells expressing NHLH1 (**S1 Text**). Luciferase activity measured after 24 hours was 4-fold higher with a construct corresponding to the risk allele as compared to the wild-type allele (**S4 Fig**, *P* < 0.001), indicating that the risk allele of rs9885413 substantially increases enhancer activity.

### Effects on DNA methylation

We next explored the association of rs9885413 with DNA methylation at the locus, providing functional evidence of epigenetic association and regulation of gene expression. DNA methylation was determined by a microarray assaying in total over 480 000 CpG methylation sites in whole blood samples from 2408 participants of the FHS. Of the 84 CpG methylation sites on the microarray within +/- 500 kb of the SNP, two were significantly associated with rs9885413: cg21070081 (beta 0.017 per T allele, *P* = 9.0x10^-69^) and cg02061660 (beta -0.015 per T allele, *P* = 4.5x10^-40^), thus constituting strong methylation quantitative trait loci (mQTLs) at the locus. Other, correlated SNPs at the locus were more strongly associated with each of these mQTLs as shown in **S5 Fig**: rs244431 for cg21070081 (*P* = 6.7x10^-369^) and rs72774805 for cg02061660 (*P* = 7.0x10^-85^). The SNP rs72774805 (perfect proxy SNP rs3844597 used) but not rs244431 was associated with heart failure mortality (*P* = 3.3x10^-3^ and 0.08, respectively), indicating that the methylation site cg02061660 is more strongly related to the underlying signal for heart failure mortality. The association of rs9885413 with lower probability of methylation at cg02061660 was replicated in 731 participants from the Rotterdam study (beta -0.029 per T allele, *P* = 1.7x10^-11^). Adjustment for cell types from direct measurement instead of estimates from methylation patterns did not abolish the association (beta -0.029 per T, *P* = 1.2x10^-6^). Interestingly, differential methylation at this CpG site was also correlated with a SNP at the locus previously associated with allergic sensitization [27] (rs10056340, *P* = 4.7x10^-29^ for mQTL), suggesting a link to inflammatory disease. This SNP was also modestly correlated with rs9885413 (r^2^ = 0.28) and associated with heart failure mortality (*P* = 0.01). The association of cg02061660 with rs9885413 (*P* = 0.52) and rs10056430 (*P* = 0.87) was abolished in analyses conditioning for rs72774805, for which the association was also markedly attenuated (*P* = 7.0x10^-33^ and 2.1x10^-46^, respectively) indicating that these correlated SNPs may reflect the same underlying signal.

### Effects on gene expression

We further assessed the association of rs9885413 with gene expression. No gene was significantly associated with rs9885413 in the diverse tissues from the Gene-Tissue Expression (GTEx) project [28] after correction for multiple tests (**S1 Text**, **S9 Table**), although conclusions were limited by a small sample size.

We next assessed association of the SNP with gene expression in two large datasets with each of the tissues most relevant for the phenotype under study: heart tissue and whole blood. We observed no convincing evidence of association (**S1 Text**, **S10 Table**) with gene expression in 247 left ventricular samples from patients with advanced heart failure (n = 116) undergoing transplantation and from unused donors (n = 131). Finally, we tested the association of rs9885413 with the expression of genes at the locus in whole blood from 5257 FHS participants [29], and with DNA methylation at cg02061660 among 2262 FHS participants. All five genes at the locus (**Fig 1**) except *TMEM232* were expressed in blood. We did not observe association of the SNP rs9885413 with any transcript, but expression of one gene (*TSLP*) was significantly associated with the methylation status of cg02061660 (*P* = 1.1x10^-4^).

The *TSLP* gene encodes a cytokine released from epithelial cells that induces release of T cell-attracting chemokines from monocytes, promotes T helper type 2 cell responses, enhances maturation of dendritic cells and activates mast cells. It has also been linked to angiogenesis and fibrosis. A monoclonal antibody targeting and inhibiting *TSLP* is currently in clinical phase III trials for asthma and allergic inflammation after a promising phase II trial [30–32]. In the myocardium, the *TSLP* gene has very low expression (**S10 Table**) but expression has been described in mature myocardial fibroblasts, which are abundant in the myocardium but of substantially smaller volume than cardiomyocytes and likely contribute little to the overall myocardial RNA pool [31,32].

To examine whether the transcription factor NHLH1 affects the expression of any of the five genes in the locus (**Fig 1**), we knocked down NHLH1 in HEK293 cells using siRNAs. A 50% decrease in NHLH1 mRNA levels was seen 48 hours after transfection, confirming efficient knock down (p<0.05, **S6A Fig**). TSLP was the only gene at the locus affected by NHLH1 knock down, showing a 30% decrease compared to cells transfected with negative control siRNA (p<0.05, **S6A Fig**). Moreover, we observed a dose-response relation between level of NHLH1 knockdown and expression of TSLP in HEK293 cells (r^2^ = 0.74, p<0.0001, **S6B Fig**).

### Population genetic analysis

Finally, distribution of the risk allele of rs9885413 in human populations was assessed using data from HapMap phase II. The derived (non-ancestral) T allele (risk allele for mortality) was highly differentiated among human populations (**S7 Fig**) having risen to an allele frequency of 0.59 in a Nigerian population (HapMap YRI sample) but only 0.06 in a European population (CEU sample). The fixation index (F_st_), a measure of population differentiation in allele frequencies, for comparison of YRI and CEU was 0.48 and more extreme F_st_ was observed in only 2.4% of SNPs in the HapMap phase 2 dataset. Consistent results were observed for another signature of recent positive selection, based on longer runs of haplotype homozygosity in carriers of the derived allele (standardized integrated haplotype score -0.766 in YRI, where negative score values indicate longer haplotypes on the background of the derived allele) [33]. These observations are consistent with positive selection in recent human history, with a selective sweep resulting in high frequency of the derived allele in western African populations. These findings are of particular interest as HF mortality is well known to be higher in populations of African ancestry, although the current study has not tested for the association with HF mortality in such populations [34].

## Discussion

We identified a SNP on chromosome 5q22 associated with increased mortality in subjects with HF. Although previous genome-wide association studies have described hundreds of loci associated with risk of disease onset, few have examined prognosis in subjects with manifest disease. This approach has the potential to generate targets for novel disease-modifying medications. Through a series of analyses *in silico* and *in vitro* we show that the SNP is located in an enhancer region, and confers increased activity of this enhancer.

Interestingly, mice deficient in the transcription factor NHLH1 predicted to bind a motif in this enhancer region have been reported to be predisposed to premature, adult-onset unexpected death in the absence of signs of cardiac structural or conduction abnormalities. NHLH1 has also been shown to regulate expression of key inflammatory cytokines such as interleukin-6 and tumor necrosis factor α. The SNP was not associated with any electrocardiographic, endocrine, or echocardiographic marker of increased risk in the general population, suggesting a mechanism specific to heart failure, an extracardiac pathway of importance in cardiac pathophysiology, or interaction with therapy for heart failure which we were unable to further test given the inception cohort design of this study. We also did not observe any robust eQTL associations for the SNP in heart. The SNP was however associated with a DNA methylation signature in whole blood that was also associated with a SNP previously associated with allergy, and with expression of the cytokine TSLP in blood. Knockdown of NHLH1 also resulted in lower expression of TSLP in HEK293 cells. This non-coding SNP may thus exert an influence on *TSLP* expression via altered NHLH1 enhancer function and DNA methylation at the methylation site cg02061660. Detailed characterization of causal variants and different association signals at the locus would however require finemapping and sequence data.

The TSLP cytokine is released from epithelial cells and fibroblasts and is considered important in initiation of inflammatory responses to tissue damage, particularly in the type 2 T-helper (Th2) pathways. Th2 pathways are central in the response to extracellular parasites but also play a key role in the pathophysiology of allergies and hypersensitivity reactions. A small subset of HF is known to be caused by Th2-mediated inflammation (eosinophilic cardiomyopathy), yet Th2 cells have received limited attention in HF pathophysiology. Recent experimental work implicates an important role of T-helper cells in HF progression for both systolic and diastolic heart failure, but has mainly focused on type 1 T-helper pathways [35,36]. It remains unclear if the mechanism for rs9885413 is through a specific etiology characterized by high mortality such as eosinophilic cardiomyopathy or a pathway involved in outcomes with manifest disease. The lack of association with HF incidence suggests that it may not act through incidence of a specific etiology, although firm conclusions are limited by sample size. We did not observe significant associations of the SNP with gene expression in any tissue. It is possible that adequately powered samples with a specific cell subtype in a specific context is needed to detect such associations, as illustrated by a recent study which only observed certain eQTLs with single-cell but not across averaged cells [37]. Indeed, baseline expression of *TSLP* was low in our samples, and is induced by tissue injury, microbes, viruses and proinflammatory cytokines [38].

Evidence of recent positive selection in individuals of African descent suggests that the HF risk allele may have been beneficial in some environments in recent human history. Inflammatory pathways are enriched for signals of recent positive selection, reflecting that infectious disease has been an important cause of mortality throughout recent evolution. Genes such as *HBB* and *APOL1* have also been reported to have been subject to recent positive selection in Africa by conferring protection against infectious diseases such as Malaria and Trypanosomiasis (sleeping sickness) [39], and *APOL1* alleles have also been linked to cardiovascular disease [40]. As cardiovascular disease and heart failure often presents after reproductive age, increased mortality in such patients would not be expected to exert purifying (negative) selective pressure. Whether SNPs at 5q22 contribute to higher mortality in subjects of African ancestry remains to be shown.

Thus, although additional work is needed to further clarify the tissues and pathways perturbed by this genetic variant and the mechanisms linking it to mortality in HF patients, the current findings implicate rs9885413 as a novel marker of increased risk among patients with HF. Complementary epigenomic evidence demonstrated candidate regions and genes, which may be mediators in cardiac pathophysiology and potential therapeutic targets to improve prognosis in patients with HF.

## Materials and Methods

### Genome-wide association study stage 1

A genome-wide association (GWA) study was performed in a total of 2,828 subjects of European ancestry with HF from seven samples collected within five large community-based prospective cohort studies including the Atherosclerosis Risk in Communities (ARIC and ARIC2) Study, the Cardiovascular Health Study (CHS), the Framingham Study (FHS), the Health ABC (Health ABC) study and the Rotterdam Study (RS and RS2). Sample characteristics, data collection and clinical definitions have been described previously and are summarized in **S1 Text**. [41–46] First diagnosis of heart failure (new-onset) was ascertained using a variety of methods based on international published criteria, as detailed in **S1 Table**. Mortality was ascertained from telephone contacts with relatives and from medical records, death certificates and/or municipal records (**S1 Text**).

Genotyping was performed using commercially available assays for genome-wide SNP detection. Imputation of non-genotyped SNPs was performed using CEU reference panels of SNP correlations from the HapMap project phase II (**S1 Text)**, to characterize a total of 2.5 million SNPs. Imputation quality was assessed for each SNP from the ratio of observed over expected variance of allele dosage.

All-cause mortality following initial HF diagnosis was examined for association with additive allele dosage of each genotyped or imputed SNP using Cox proportional hazards models, with censoring at the end of or loss to follow-up. Models were adjusted for age at diagnosis, sex, and recruitment site in multicenter cohorts. In the family-based FHS, Cox models were implemented with clustering on pedigree to account for relatedness. Genomic control was applied to results from each cohort. Cohort-specific GWA results were combined using fixed effects meta-analysis with inverse variance weights. SNPs were excluded from cohort-level analyses if exhibiting implausible beta coefficients (< -5 or > 5) and from the meta-analysis for low heterozygosity (sample size-weighted minor allele frequency ≤ 0.03, corresponding to < 100 minor allele carriers with an endpoint).

### Stage 2 genotyping in independent samples and combined analysis

SNPs passing a *P*-value threshold defined *a priori* as *P* < 5.0x10^-7^ in the genome-wide stage 1 were carried forward to the second stage with targeted genotyping in 1,870 HF patients from four independent cohorts. For 2.5 million tests, this threshold limits the expected number of genome-wide false positives to approximately 1, assuming statistical independence of tests. The second stage included four independent cohorts; the Malmö Diet and Cancer Study (MDCS), the Malmö Preventive Project (MPP), the Physicians’ Health Study (PHS) and the Prospective Study of Pravastatin in the Elderly at Risk (PROSPER) [47–50]. Heart failure ascertainment and time of death in these cohorts was similar to in stage 1 cohorts, as shown in **S1 Table** and **S1 Text**. Genotyping was performed as outlined in **S1 Text**. Association analyses and meta-analysis of results were performed as in the first stage. Meta-analysis of stage 1 and 2 was performed, and a combined *P*-value < 5.0x10^-8^ was considered significant. Heterogeneity was assessed across the combined stage 1 and 2 cohorts using Cochran’s Q test for heterogeneity, computed as the sum of the squared deviations of each study’s effect from the weighted mean over the study variance, and the I^2^ test, the percentage of total variation across studies that is due to heterogeneity rather than chance (I^2^ = [Q—df] / Q) [51, 52].

### In silico studies of effect on cardiac structure and function

The association of replicated SNPs with measures of cardiac structure and function was evaluated from summary results of the following GWA consortia: EchoGen [19], CHARGE-HF [20], CHARGE-QRS [22], natriuretic peptides in 5453 subjects from the Malmö Diet and Cancer study [21], QT-IGC [23], and the CHARGE Sudden Cardiac Death consortium (manuscript in preparation). Each of these consortia is described in **S1 Text**.

### Interrogation of functional motifs

The correlation of replicated SNPs with known coding SNPs was examined in the databases for the 1000 Genomes Project and phase III of the HapMap project, using SNAP [53]. The location of SNPs in relation to regulatory motifs was explored using histone methylation patterns generated as part of the ROADMAP Epigenomics project [24]. Enhancers were identified in each of the 129 ROADMAP tissues using the ChromHMM algorithm [54] from patterns of monomethylation (H3K4Me1) of the fourth residue (lysine) and acetylation of the 27th residue (H3K27Ac) of histone H3. The location of SNPs in relation to transcription factor binding sites was assessed in silico using HaploReg version 4.1 (http://www.broadinstitute.org/mammals/haploreg/haploreg.php) [55] and the UCSC Genome Browser (http://genome.ucsc.edu). In HaploReg, position weight matrices (PWMs; probabilistic representations of DNA sequence) were computed with p-values based on literature sources and ENCODE ChIP-Seq experiments as previously described [55], and only instances where a motif in the sequence passed a threshold of *P* < 4^−7^ were considered. The NHLH1-binding motif was retrieved into HaploReg from the manually curated TRANSFAC database.

### In vitro assessment of enhancer activity

Complementary DNA oligonucleotides corresponding to the 100 bp genomic region flanking rs9885413 (50 bp on either side of the SNP) were cloned into the luciferase reporter vector pGL3-Promoter (Promega, Madison, WI) using the MluI and BglII sites. Two different sets of oligos were cloned, one corresponding to the major allele of rs9885413 (pGL3P-G) and one to the minor allele (pGL3P-T). Oligonucleotide sequences were as following: major allele sense: CGCGTCCTGCCTCACATAATCTTTTTGTTTGTCCCCCTGAAATGGATTCTCAGCTGTTGCCCAAACATTTCATCTTAGCGTTCCAGGTTTGAACTCGCCCTCACGA, minor allele sense: CGCGTCCTGCCTCACATAATCTTTTTGTTTGTCCCCCTGAAATGTATTC TCAGCTGTTGCCCAAACATTTCATCTTAGCGTTCCAGGTTTGAACTCGCCCTCACGA, and the corresponding antisense sequences. The reporter vectors were co-transfected with the pRL-null vector at a ratio of 10:1 into HEK293 cells using Lipofectamine LTX (Life Technologies) according to the manufacturer’s instructions. 24 hours post-transfection, luciferase activity was assayed using the Dual-Luciferase Reporter Assay System (Promega) and Glomax 20/20 Luminometer (Promega). The signal from the reporter vector was normalized to the signal from the pRL-null vector.

### Gene expression in heart

Samples of left ventricular cardiac tissue from patients undergoing cardiac surgery were genotyped for the SNP rs9885413 and for all five transcripts within +/- 500 kb of the SNP. Samples of cardiac tissue were acquired from patients from the MAGNet consortium (http://www.med.upenn.edu/magnet/). Gene expression levels were determined using the Affymetrix ST1.1 gene expression array (Affymetrix, Santa Clara, CA, USA) in a cohort including 247 heart samples. Genotyping was performed using the Illumina OmniExpress array. Left ventricular free-wall tissue was harvested at time of cardiac surgery from subjects with heart failure undergoing transplantation or from unused transplant donors. In all cases, the heart was perfused with cold cardioplegia prior to cardiectomy to arrest contraction and prevent ischemic damage. Tissue specimens were then obtained and frozen in liquid nitrogen. Genomic DNA from left ventricle was extracted using the Gentra Puregene Tissue Kit (Qiagen) according to manufacturer’s instruction.

Total RNA was extracted from left ventricle using the miRNeasy Kit (Qiagen) including DNAse treatment on column. RNA concentration and quality was determined using the NanoVue Plus spectrophotometer (GE Healthcare) and the Agilent 2100 RNA Nano Chip (Agilent). For all samples, genome-wide SNP genotypes were generated using the Illumina OmniExpress Array. Caucasian Ancestry was verified using multi-dimensional scaling of genotypes. For Gene expression array experiments, the Affymetrix ST1.1 Gene array was used. Data were normalized using the Robust Multi-array Average algorithm and batch effects were adjusted for using ComBat. Transcript expression levels were considered significantly higher than background noise if expression values from robust multiarray analysis in at least 10% of either cases or controls exceeded of the 80% quantile of expression of genes on the Y-chromosome in female hearts (5.24). Associations of expression levels for expressed genes with SNP genotypes were tested using a likelihood ratio test. Specifically, we fit a linear regression model Y = β0 + β1*D + β2*g + β3*(g x D) where Y is the log_2_ transformed expression level of a given probe, g is the genotype (coded as 0, 1, and 2) of the test SNP, and D is heart failure disease status (D = 1 for heart failure cases and D = 0 for unused donor controls). Association between the probe and test SNP was assessed by testing H0: β2 = β3 = 0 using a likelihood ratio test. Significance of the test statistic was evaluated by comparing with a Chi-squared distribution with two degrees of freedom. All models were additionally adjusted for age, gender, and study site.

### DNA methylation and gene expression in whole blood

The association of the SNP rs9885413 with DNA methylation was examined in 2408 participants from the FHS Offspring cohort. Methylation at cytosine-guanine dinucleotides (CpG) at the 5q22 locus (+/-500 kb from rs9885413) were ascertained from a gene-centric DNA methylation array (Infinium HumaMethylation450 BeadChip, Illumina, San Diego, CA, USA) which allows interrogation of 485,512 methylation sites across the genome. The array has coverage of at least one methylation site near 99% of RefSeq genes and 96% of CpG islands. Briefly, bisulfite-treated genomic DNA (1 μg) from peripheral blood samples underwent whole-genome amplification, array hybridization and scanning according to manufacturer instructions. Genotyping of rs9885413 was performed as described in **S1 Text**. Association of rs9885413 and the methylation probe cg02061660 with expression of the five genes at the locus (+/-500 kb from rs9885413) was examined from microarray data (Affymetrix Human Exon Array ST 1.0) in 5257 participants from the FHS Offspring cohort and Third Generation cohort. Procedures for RNA extraction, processing and analysis have been described previously (28). Linear mixed effect (LME) models were fit accounting for familial correlation, cell count heterogeneity and technical covariates to account for batch effects using the pedigreemm package in R [56]. Specifically, the mQTL model utilized a two-step approach: first, the DNA methylation beta-value (ratio of methylated probe intensity to total probe intensity) was residualized with adjustment for age, sex, cell count proportions (imputed using the Houseman method for granulocytes, monocytes, B-lymphocytes, CD4+ T lymphocytes, CD8+ T lymphocytes and NK cells) [57], measured technical covariates (row, chip, column), and the family structure covariance matrix. Second, DNA methylation residuals were specified as dependent variable, SNP genotype dosage as independent variable with additional adjustment for 558 SVAs (surrogate variable analysis) [58] and ten principal components from eigenstrat [59] to account for unmeasured batch effects. The eQTL models similarly residualized gene expression with adjustment for age, sex, imputed cell count proportions (imputed in Offspring Cohort participants utilizing gene expression markers of cell count proportions developed from the Third Generation participants with both gene expression and measured complete blood counts), and family structure covariance matrix. The residual of gene expression was specified as dependent variable and SNP dosage as independent variable adjusted for 20 PEER (probabilistic estimation of expression residuals) factors [60] to account for unmeasured technical and batch effects in the gene expression data. The eQTM models specified gene expression residual as dependent variable and DNA methylation residual as independent variable adjusted for 20 methylation SVAs and 20 expression SVAs to account for unmeasured technical and batch effects.

Replication of the association of rs9885413 with cg02061660 including the same covariates in the model as in FHS was attempted in blood samples from 750 randomly selected participants of the Rotterdam study (RS3) not included in the GWA stage, where information from the same DNA methylation array as FHS was available. DNA was extracted, bisulfite-treated using the Zymo EZ-96 DNA-methylation kit (Zymo Research, Irvine, CA, USA) and hybridized to arrays according to manufacturer instructions. During quality control samples showing incomplete bisulfite treatment were excluded (n = 5) as were samples with a low detection rate (<99%) (n = 7), or gender swaps (n = 4). Probes with a detection *P*-value>0.01 in >1% samples, were filtered out. A total number of 474,528 probes passed the quality control and the filtered β values were normalized with DASEN implemented in the *wateRmelon* package in R statistical software. Genotyping was performed using the Illumina 610quad array. Cell counts were estimated using the same method as in FHS and also directly measured on a Coulter AcT Diff II Hematology Analyzer (Beckman Coulter, Brea, CA) for granulocytes, monocytes, lymphocytes). Models including both estimated and directly measured cell counts were explored.

### siRNA-mediated knock down of NHLH1

HEK293 cells were seeded at 100,000 cells/well in a 6-well plate the day before transfection. Cells were transfected using Lipofectamine and 50 nM of siRNA designed to target human NHLH1 or negative control siRNA (Life Technologies, Carlsbad, CA, USA) according to the manufacturer’s instructions. After 48 hours, cells were harvested and total RNA extracted using the miRNeasy Mini Kit (Qiagen, Hilden, Germany) according to the manufacturer’s instructions. cDNA was synthesized using the RevertAid H- First Strand cDNA Synthesis Kit (Thermo Fischer Scientific, Waltham, MA, USA) using random hexamer primers and qPCR was performed with TaqMan assays for NHLH1, TMEM232, SLC25A4, WDR36, TSLP, CAMK4 and GAPDH on a StepOne Plus Real-Time PCR System (Life Technologies). Gene expression was normalized to GAPDH and expressed relative to cells transfected with negative control siRNA according to the ΔΔCt-method [61].

### Population genetic analysis

The frequencies of ancestral and derived alleles of rs9885413 were examined in populations from the International HapMap Project (http://www.hapmap.org/) [62] and the Human Genome Diversity Project (HGDP, http://hagsc.org/hgdp/) [63]. The fixation index (F_st_) was estimated as described by Weir and Cockerham [64], based on allele frequencies in HapMap stage II as also previously described [65]. The integrated haplotype score (iHS) was calculated from HapMap stage II data as described by Voight et al (http://haplotter.uchicago.edu/) [33]. Allele frequency distributions in HGDP populations were visualized using the HGDP selection browser (http://hgdp.uchicago.edu/) [66].

### Ethics statement

Informed consent was obtained from all participants and all contributing studies were approved by the respective ethics committee as described in **S1 Text**.

## Supporting Information

S1 Text Supplementary Materials, Methods and Results. (DOCX)

S1 Fig Quantile-quantile plots for individual genome-wide association studies of HF mortality. Plotted on the x-axis are expected *P*-values under the null hypothesis and on the y-axis the observed *P*-values before genomic control has been applied.
(PDF)

S2 Fig Quantile-quantile plot for meta-analysis of genome-wide association studies of HF mortality. Plotted on the x-axis are expected *P*-values under the null hypothesis and on the y-axis the observed *P*-values.
(PDF)

S3 Fig HF mortality association results for 2.5 million SNPs across 22 autosomes in stage 1 cohorts. Each dot represents one SNP. The x-axis shows physical position by chromosome and the y-axis–log_10_ (*P*-value). The vertical dotted line indicates the a priori specified significance threshold for SNPs to be carried forward to stage 2 (*P* < 5.0x10^-7^).
(PDF)

S4 Fig Increased enhancer activity by the lead SNP. HEK293 cells were transfected with either empty vector (pGL3P) or vectors containing the major (pGL3P-G) or minor (pGL3P-T) allele of rs9885413 together with 100 bp flanking the SNP. Luciferase activity from the vectors was measured after 24 hours and normalized to that of a pRL-null vector. N = 3. ***P < 0.001.
(PDF)

S5 Fig Association of rs9885413 with DNA methylation. The plot covers the genomic region of +/-1 million bases from each CpG methylation. P-values refer to association of each SNP with two CpG methylation sites on chromosome 5q22: cg02061160 (top panel) and cg21070081 (bottom panel). Circles represent SNPs. The purple circle represents the SNP associated with heart failure mortality (rs9885413). Circle color represents strength of pairwise correlation with rs9885413, with r^2^ according to the inset. CpG sites are illustrated below the regional plot, as is the location of the SNP associated with allergic sensitization (rs10056340). Recombination rate is plotted in the background and known genes are represented in the bottom of the plot. Positions refer to NCBI build 36. SNP correlations and recombination rates were obtained from the 1000 Genomes pilot and HapMap release 22, respectively. The plot was created using LocusZoom (http://locuszoom.sph.umich.edu/locuszoom/).
(PDF)

S6 Fig siRNA-mediated knock down of NHLH1. HEK293 cells were transfected with siRNA targeted against NHLH1 and expression of the five genes surrounding rs9885413 were analyzed by qPCR. Expression levels were normalized to the housekeeping gene GAPDH and expressed relative to cells transfected with negative control (NC) siRNA. Results are from three separate experiments with triplicates in each sample group. *p<0.05 in two sample t-tests. A) Fold change for NHLH1 and individual genes at the locus. B) Dose-response relation of NHLH1 knockdown and TSLP expression.
(PDF)

S7 Fig Distribution of rs9885413 alleles in HGDP populations. Relative allele frequencies of the ancestral (G, blue) and derived (T, yellow) alleles of rs9885413 presented as pie slices across populations in the Human Genome Diversity Project (HGDP).
(PDF)

S1 Table Methods of heart failure ascertainment and diagnosis across cohorts. *Included non-hospitalized deaths but not morbid events. **European Society of Cardiology. ***In a validation sample as previously described [26]. ^†^Signs and symptoms included in Framingham criteria. ^‡^Cardiac structure and function including cardiomegaly, dilated ventricle, decreased systolic function, or segmental wall-motion abnormalities. ^§^Pharmaceutical treatments include the prescribing of diuretics, digoxin, or vasodilator.
(DOCX)

S2 Table Characteristics of stage 2 cohorts. Age, body mass index and follow-up time are presented as mean (standard deviation). Categorical variables are presented as percentages. Body mass index, diabetes, hypertension and smoking refer to the nearest study exam prior to heart failure diagnosis whereas age, follow-up time, mortality rate and all-cause death refer to the time of HF diagnosis. Mortality rate refers to the 1-year Kaplan-Meier estimate, with censoring at loss to follow-up. MI: Myocardial infarction.
(DOCX)

S3 Table Results for all SNPs with *P* < 1.0x10^-5^. Genome position (POS) refers to NCBI build 36. CHR, chromosome. STR, strand. CA, coded allele. A2, non-coded allele. N, sample size. BETA, beta estimate. SE, standard error of beta estimate. *P*, *P*-value.
(DOCX)

S4 Table Cohort-specific results for SNPs in combined stages 1 and 2. CHR, chromosome. CA, coded allele. A2, non-coded allele. Caf, coded allele frequency. N, sample size. Beta, beta estimate. Se, standard error of beta estimate. P, p-value.
(DOCX)

S5 Table Allele frequency distribution across major causes of mortality. (DOCX)

S6 Table In silico studies of associations of rs9885413 with cardiac structure and function. (DOCX)

S7 Table Enhancer annotations in the ROADMAP Epigenomics Project. Enhancer regions in the 129 tissues from the ROADMAP Epigenomics Project, as determined from the ChromHMM algorithm from patterns of monomethylation of the fourth residue (lysine) of histone H3 (H3K4Me1). Enhancers overlapping the lead SNP on chromosome 5q22 (rs9885413) and strongly correlated SNPs (r^2^ > 0.8), with enhancers overlapping the lead SNP shown in bold.
(DOCX)

S8 Table Location of rs9885413 in the predicted NHLH1 binding site. * The two alleles of the SNP rs9885413.
(DOCX)

S9 Table Association of rs9885413 with gene expression in the GTEx Project. Association of rs9885413 with expression of the five genes at the chromosomal locus in 13 tissues from the Gene-Tissue Expression Project with data available in > 60 samples. Empirical *P*-values were computed using a permutation approach as described in documentation for the GTEx project (http://www.gtexportal.org). N/A, no results available in GTEx. Effect direction is presented for associations with *P* ≤ 0.1.
(DOCX)

S10 Table Association of rs9885413 with gene expression in human heart. Association of rs9885413 with expression of the five genes at the locus on chromosome 5q22 in 247 human heart samples from 116 patients with advanced heart failure (HF) and 131 unused transplant donor hearts (controls). Transcript expression levels were considered significantly higher than background noise if expression values from robust multiarray analysis in at least 10% of either cases or controls exceeded of the 80% quantile of expression of genes on the Y-chromosome in female hearts (5.24). Positive direction indicates higher expression with the risk (minor) allele of rs9885413.
(DOCX)
