# Supplementary material for: Discovery of Genetic Variation on Chromosome 5q22 Associated with Mortality in Heart Failure
Source: PLoS Genet. 2016 May 5;12(5):e1006034. doi: 10.1371/journal.pgen.1006034 (PMC4858216; doi:10.1371/journal.pgen.1006034)
Supplement: S1 Text — (DOCX) [file pgen.1006034.s001.docx]

Discovery of genetic variation on chromosome 5q22 associated with mortality in heart failure

**S1 Text. Supplementary Materials, Methods and Results**

**1 Cohort descriptions**

The present study was initiated within the investigator-driven collaboration of the Cohorts for Heart and Aging in Genomic Epidemiology (CHARGE) consortium, including several population-based cohorts with genome-wide SNP data and comprehensive individual phenotyping [1,2].The discovery meta-analysis included participants from seven samples collected within five cohort studies. In a second stage, association was studied in individuals with new-onset heart failure from four independent cohorts. Participants were of European ancestry. The included cohorts are described in detail below. Definitions of incident HF employed in different studies are summarized in S1 Table.

**1.1 Stage 1 (discovery) cohorts**

**1.1.1 Atherosclerosis Risk in Communities study (ARIC and ARIC 2)** The ARIC Study is a prospective investigation of atherosclerosis and its clinical sequelae involving 15,792 individuals aged 45-64 years at recruitment (1986-1989). Subjects were selected by probability sampling from four communities: Forsyth County, NC; Jackson, MS (African-Americans only); Northwestern suburbs of Minneapolis, MN; and Washington County, MD. The initial clinical exams included a home interview to ascertain cardiovascular risk factors, socioeconomic factors, and family medical history, clinical examination and blood drawing for laboratory determinations. Medical events were identified by an annual phone interview, at a total of 4 three-year cycles of exams, and hospital and death certificate surveillance. A detailed description of the ARIC study design and methods has been published elsewhere [3].

For the purpose of this study, the cohort labeled ARIC represents the subset of individuals genotyped for genome-wide SNPs as previously described [2]. ARIC2 represents an independent subset of individuals that were genotyped for the same set of genome-wide markers and where not represented in the set of individuals described in Morrison et al, 2010. [1] Subjects of self-reported African American ancestry were excluded from the current study. For these analyses, measures of clinical and demographic characteristics were obtained from the clinical visit preceding HF onset. Deaths were defined as all-cause mortality. In the cohort labeled ARIC, deaths were ascertained through annual phone calls or through ongoing surveillance of health department death certificate files through December 31, 2004. In the cohort labeled ARIC2, deaths were ascertained through annual phone calls or through ongoing surveillance of health department death certificate files through December 31, 2005.

**1.1.2 Cardiovascular Health Study (CHS)** The CHS is a population-based cohort study of risk factors for CHD and stroke in adults ≥65 years conducted across four field centers [4]. The original predominantly Caucasian cohort of 5,201 persons was recruited in 1989-1990 from random samples of the Medicare eligibility lists; subsequently, an additional predominantly African-American cohort of 687 persons was enrolled subsequently for a total sample of 5,888. DNA was extracted from blood samples drawn on all participants at their baseline examination in 1989-90. In 2007-2008, genotyping was performed at the General Clinical Research Center's Phenotyping/Genotyping Laboratory at Cedars-Sinai using the Illumina 370CNV [BeadChip](http://wildebeest.pbworks.com/BeadChip) system on 3980 CHS participants who were free of CVD at baseline, consented to genetic testing, and had DNA available for genotyping. A total of 1908 persons were excluded from the genome-wide association (GWA) study sample due to the presence at study baseline of coronary heart disease, congestive heart failure, peripheral vascular disease, valvular heart disease, stroke or transient ischemic attack or lack of available DNA. Because the other cohorts were predominantly of European ancestry, the African ancestry participants were excluded from this analysis to reduce the possibility of confounding by population structure.

Incident HF events were identified during follow-up by annual or semi-annual self-report and from administrative data [5]. Events identified by self-report or administrative data were validated by physician review of medical records using published criteria. Participant mortality was identified at 6-month surveillance contacts, National Death Index searches, and from obituaries. Additional information about cause of death was collected from death certificates, proxy interviews, and medical records [5].

**1.1.3 Framingham Heart Study (FHS)** The methods of recruitment and data collection have been described previously for the original Framingham Heart Study cohort (5,209 participants ascertained systematically from two-thirds of the households in the town of Framingham, MA, beginning in 1948) [6], the Framingham Heart Study Offspring cohort (5,124 children of the original cohort, and spouses of those children, beginning in 1972) [7], and the Third Generation cohort (4,095 children of the Offspring cohort, beginning in 2002) [8]. In the mid-1990s DNA was collected from the Original and Offspring study participants with the purpose of conducting genetic studies and developing cell lines. At the time of DNA draw, there were 4270 participants free of CHF in these two cohorts. Of these 4270, 249 individuals developed incident CHF. The current study was conducted in these 249 participants of the Original and Offspring cohorts, who were free of CHF at the time of their blood draw for a DNA sample and then developed incident CHF. Of the 249 with incident CHF, 156 died subsequently over the subsequent 10 years of follow up. There is continuous follow up for all cardiovascular and death events. An endpoint Committee consisting of three physicians reviewed all suspected congestive heart failure events to confirm occurrence of the event, using previously agreed criteria. The same committee also reviews all deaths. CHF is defined as satisfying the previously published Framingham criteria* (presence of 2 major, or of one major plus two minor criteria in the absence of a competing explanation) and adjudicated by a panel of three experienced investigators. The major criteria are Paroxysmal nocturnal dyspnea or orthopnea, Distended neck veins, Hepatojugular reflux, Rales, S3 gallop, Enlarged heart by X-ray, Acute pulmonary edema on chest X-ray, Treatment induced weight loss >10lbs/5 days, Increased venous pressure > 16 cm water, Pulmonary edema and visceral congestion or cardiomegaly on autopsy. The minor criteria are Dyspnea on ordinary exertion, Night cough, Heart rate > 120/minute, Hepatomegaly, Ankle edema, Decrease in vital capacity by 1/3^rd^, Pleural effusion by X-ray and Pulmonary vascular engorgement by X-ray.

**1.1.4 Health, Aging and Body Composition (Health ABC)** The Health ABC Study is a population-based study of 3075 community-dwelling men and women aged 70 to 79 years at enrolment. To be eligible, participants had to report no difficulty in walking one-quarter mile or climbing 10 stairs without resting. Participants were identified from a random sample of white Medicare beneficiaries and all age-eligible black community residents in designated zip code areas surrounding Pittsburgh, Pennsylvania, and Memphis, Tennessee, USA. Exclusion criteria included difficulties with activities of daily living, obvious cognitive impairment, inability to communicate with the interviewer, intention of moving within 3 years, or participation in a trial involving a lifestyle intervention. In addition, subjects with self-report African American ancestry were excluded from the current study. All participants provided written informed consent, and the institutional review boards at both study sites approved the study protocol. Baseline data were collected from April 1997 to June 1998. Buffy coat samples for subsequent DNA analyses were drawn at baseline visit.

Cardiovascular disease status at baseline, including prevalent heart failure, was based on self-reported history, use of selected drugs, and *International Classification of Diseases, Ninth Revision, Clinical Modification* codes as reported by Medicare and Medicaid Services from 1995 through 1998. All participants were asked to report any hospitalizations, and every 6 months they were asked direct questions to elicit information about interim cardiovascular events. All admissions with an overnight stay were evaluated for cardiovascular events by reviewing the medical records at each site. All first admissions with an overnight stay confirmed to be related to heart failure were classified as incident heart failure. Local adjudicators classified events as heart failure based on symptoms, signs, chest radiograph results, and echocardiographic findings based on criteria similar to those used in the Cardiovascular Health Study [9]. The heart failure criteria required at least a heart failure diagnosis from a physician and treatment for heart failure (i.e., a current prescription for a diuretic agent and either digitalis or a vasodilator); these criteria have been used in previous investigations. Because heart failure was not allowed as a cause of death, there were no immediate deaths from incident heart failure. The Health ABC Study Diagnosis and Disease Ascertainment Committee reviewed all hospitalizations and deaths, and underlying causes of death were determined by central adjudication.

**1.1.5 Rotterdam Study (RS and RS2)** The Rotterdam Study is a prospective population-based cohort study to investigate the determinants of chronic diseases among participants aged 55 years and older, of which the methods have been described previously [10]. Briefly, all residents of Ommoord, a district of Rotterdam, the Netherlands, aged 55 years or older, were asked to participate in 1990. 7983 (78%) participated. The baseline examination, conducted in 1990-1993, consisted of a home interview and research center visit, during which blood samples were taken. After baseline examination, participants were invited to visit the research center for extensive testing every 3-4 years. In 2000, inhabitants who had turned 55 years or newly moved to the study district since the start of the study were invited to participate in the Rotterdam Study 2 (RS2). 3011 (67%) participated, who underwent the baseline examination (including blood sampling) and entered into the same follow-up program. During the baseline examination and follow-up visits, measurements are performed using standardized methods, including measurement of height, weight, systolic and diastolic blood pressure. The Rotterdam Study has been approved by the Medical Ethics Committee of the Erasmus MC and by the Dutch Ministry of Health, Welfare and Sport, implementing the “Wet Bevolkings Onderzoek: ERGO (Population Screening Act: Rotterdam Study). All participants provided written informed consent to participate in the study and to obtain information from their treating physicians.

Follow-up for heart failure was complete until October 2006. The presence of heart failure was assessed using a validated score based on the definition of heart failure of the European Society of Cardiology [11]. Information on prevalent heart failure at baseline was available for all participants and participants with prevalent heart failure were excluded. Cases of incident heart failure were obtained by continuously monitoring participants of the Rotterdam Study during follow-up. The date of incident heart failure was defined as the day of the first occurrence of symptoms suggestive of heart failure, or the day of receipt of a first prescription for a loop diuretic or an inhibitor of angiotensin-converting enzyme in a patient diagnosed with heart failure, whichever came first. The diagnosis of heart failure was classified as definite, probable, possible, or unlikely. In accordance with the criteria of the European Society of Cardiology, only definite and probable cases were included in the analysis.

Deaths were ascertained through general practitioner medical records and from municipal records through January 1, 2007. The mortality ascertainment rate was greater than 99%.

**1.2 Stage 2 cohorts**

**1.2.1 The Malmö cohorts (Malmö Diet and Cancer and the Malmö Preventive Project)** Participants with prevalent or incident heart failure were identified from these two prospective, population-based cohort studies from the city of Malmö in southern Sweden. Baseline characteristics and endpoint ascertainment have been described in detail previously for both cohorts [12,13]. Informed consent was obtained from all participants and both studies were approved by the ethics committee of Lund University, Sweden.

The Malmö Diet and Cancer study (MDCS) included 30,447 randomly selected men born between 1923 and 1945 and women born between 1923 and 1950. Participants underwent a baseline examination between 1991 and 1996, with sampling of peripheral venous blood, measurement of blood pressure and anthropometric measures, and filled out a questionnaire.

The Malmö Preventive Project (MPP) included 33,346 randomly selected men and women born between 1921 and 1949. Male participants (n = 22,444) underwent baseline examinations between 1974 and 1992 female participants (n = 10,902) underwent baseline examinations between 1980 and 1992. The present study was performed in the 17 284 participants who attended a reexamination between 2002 and 2006. At the reexamination, participants underwent sampling of peripheral venous blood, measurement of blood pressure and anthropometric measures, and filled out a questionnaire.

Prevalent and incident diagnoses of HF were identified by record linkage to national registers using personal identification numbers [14]; the Swedish Hospital Discharge Register (HDR) and the Swedish Cause of Death Register (CDR) [15]. Time of death was ascertained from the CDR. Both registers are administered by the Swedish National Board of Health and Welfare. Data collection in the HDR was started in the 1960s and includes dates of admission and discharge as well as primary and contributory diagnoses from all public hospitals in Sweden. Reporting to the HDR has been compulsory since 1987 but the only hospital in Malmö (Malmö University Hospital) has reported since 1969. The CDR includes diagnoses from death certificates since 1952, regardless if death occurred outside of Sweden. Diagnoses in the HDR are coded as primary or contributory and in the CDR as underlying or contributory cause of death, both using the International Classification of Disease (ICD). The 8^th^ edition (ICD-8) was used until the end of 1986, the 9^th^ edition (ICD-9) between 1987 and 1996 and the 10^th^ edition (ICD-10) from 1997 until present. Heart failure was defined as diagnosis codes 427.00, 427.10, 428.99 (ICD-8), 428 (ICD-9), I50 or I11.0 (ICD-10) as primary or contributory diagnosis. High validity of such diagnoses have previously been reported [16].

**1.2.2 Physicians’ Health Study (PHS)** The Physicians’ Health Study is a completed randomized, double-blind, placebo-controlled trial designed to study low-dose aspirin and beta-carotene for the primary prevention of cardiovascular disease and cancer. DNA was extracted from blood samples collected between 1997-2001. All incident cases of HF occurring after blood collection among participants of European ancestry were included in the current study. A previous validation study of incident heart failure self-report confirmed valid diagnoses in 91% of cases [17]. Mortality was ascertained by an End Point Committee. Deaths were identified through systematic searches of the National Death Index for the entire enrollment cohort, and death certificates were obtained from state agencies for all deaths that occurred before February 1, 1988. The deaths were classified by trained nosologists according to the International Classification of Diseases, Ninth Revision (ICD-9), as previously described [18].

**1.2.3 Prospective Study of Pravastatin in the Elderly at Risk (PROSPER)** A detailed description of the Prospective Study of Pravastatin in the Elderly at Risk (PROSPER) has been published elsewhere [19,20]. PROSPER was a prospective multicenter randomized placebo-controlled trial to assess whether treatment with pravastatin diminishes the risk of major vascular events in elderly. Between December 1997 and May 1999, we screened and enrolled subjects in Scotland (Glasgow), Ireland (Cork), and the Netherlands (Leiden). Men and women aged 70-82 years were recruited if they had pre-existing vascular disease or increased risk of such disease because of smoking, hypertension, or diabetes. A total number of 5804 subjects were randomly assigned to pravastatin or placebo. A large number of prospective tests were performed including Biobank tests and cognitive function measurements. At baseline, blood samples were drawn and DNA was extracted. A whole genome wide screening has been performed in the sequential PHArmacogenomics of Statins in the Elderly at risk study. Heart failure was one of the tertiary endpoints of the PROSPER study, all endpoints were adjudicated by an expert committee blinded to randomized study medication and using predefined criteria. Heart failure was defined based on a combination of symptoms (e.g. shortness of breath) and signs, including chest radiograph with fluid congestion or echocardiogram with severely diminished left ventricular function.

**2 Methods for genotyping and imputation across cohorts**

Genotyping, quality control, data cleaning and imputation was performed independently in each cohort using different genetic platforms and software as outlined below.

**2.1 Stage 1 (discovery) cohorts**

**2.1.1 Atherosclerosis Risk in Communities study (ARIC and ARIC2)** Genotyping used DNA collected from phlebotomy. Genome-wide assays of SNPs were conducted using the Affymetrix 6.0 platform. Quality control and data cleaning and imputation by MACH 1.0 with genome build NCBI35 was conducted in two phases. For the purpose of this study, the cohort labeled ARIC represents the subset of individuals described by Morrison et al [1] who were evaluated in the first phase of genotype quality control, data cleaning and imputation. ARIC2 represents an independent set of individuals that were evaluated in the second phase of genotype quality control, data cleaning and imputation. The methods for genotyping and imputation were identical for ARIC and ARIC2. The influence of population stratification was assessed using principal component analysis.

**2.1.2 Cardiovascular Health Study (CHS)** Genotyping was performed at the General Clinical Research Center's Phenotyping/Genotyping Laboratory at Cedars-Sinai using the Illumina 370CNV [BeadChip](http://wildebeest.pbworks.com/BeadChip) system. Genotypes were called using the Illumina [BeadStudio](http://wildebeest.pbworks.com/BeadStudio) software. The following exclusions were applied to identify a final set of 306,655 autosomal SNPs: call rate < 97%, HWE P < 10^-5^, > 2 duplicate errors or Mendelian inconsistencies (for reference CEPH trios), heterozygote frequency = 0, SNP not found in HapMap. Imputation was performed using BIMBAM v0.99 with reference to [HapMap](http://wildebeest.pbworks.com/HapMap) CEU using release 22, build 36 using one round of imputations and the default expectation-maximization warm-ups and runs. The influence of population stratification was assessed using principal component analysis.

**2.1.3 Framingham Heart Study (FHS)** Genotyping was conducted for the SNP Health Association Resource (SHARe) project (http://www.ncbi.nlm.nih.gov/projects/gap/cgi-bin/study.cgi?study_id=phs000007.v10.p5) using the Affymetrix 500K mapping array (250K Nsp and 250K Sty arrays) and the Affymetrix 50K supplemental gene focused array on a total of 9,274 individuals from all three cohorts. Genotyping resulted in 503,551 SNPs with successful call rate >95% and HWE P>1.0x10^-6^ in 8,481 individuals with call rate >97%. Imputation of 2,543,887 autosomal SNPs in HapMap release 22, build 36, CEU sample was conducted using the algorithm implemented in MACH (version 1.0.15). From a total of 534,982 genotyped autosomal SNPs in Framingham, 378,163 SNPs were used in imputation after filtering out 15,586 SNPs (HWE P < 1.0x10^-6^), 64,511 SNPs (missingness > 0.03), 45,361 SNPs (mishap *P* < 1.0x10^-9^), 4,857 SNPs (> 100 Mendelian errors), 67,269 SNPs (frequency < 0.01), 2 SNPs (due to strand issues upon merging data with HapMap), and a further 13,394 SNPs that were not present on HapMap. We used 200 biologically unrelated participants to estimate the parameters of the imputation model and subsequently applied the estimated parameters to obtain imputed SNPs for all 8,481 participants. To evaluate population substratification, we conducted principal component analyses using EIGENSTRAT [21] on the genotypes from 882 unrelated participants. We estimated the first 10 principal components and applied the loadings of these components to all genotyped participants. Finally, we evaluated whether any of these principal components were associated with CHF. All analyses of individual SNPs adjusted for sex, age, generation (Original or Offspring Cohort), and principal components 1 and 3 using Cox proportional hazards models with robust standard errors calculated by clustering on families to account for familial correlations. The Framingham Heart Study was approved by the institutional review boards of Boston University and the National Institutes of Health. All participants provided written informed consent.

**2.1.4 Health, Aging and Body Composition (Health ABC)** Genotyping was performed using the Illumina 1M chip array. Genotypes were called using the Illumina BeadStudio software. The following exclusions were applied to identify a final set of 1,007,948 SNPs: call rate < 97%, MAF < 1% and HWE P < 10^-6^. Imputation was performed using MACH v1.16 with reference to HapMap CEU using release 22. In genetic association analyses, adjustment was performed for principal components.

**2.1.5 Rotterdam Study (RS and RS2)** Genotyping was done using the version 3 Illumina Infinium II HumanHap550 SNP chip array. Genotyping procedures were followed according to manufacturer’s protocol (Illumina, San Diego, CA, USA). Any samples with a call rate below 97.5%, excess autosomal heterozygosity, mismatch between called and phenotypic gender, or outliers identified by the IBS clustering analysis with > 3 standard deviations from population mean or IBS probabilities >97% were excluded from the analysis; in total, 5,974 samples were analyzed in RS and 2,157 in RS2. SNPs with minor allele frequency ≤ 1%, SNP call rate < 98% for Hardy-Weinberg deviations with p< 1x10^-6^ were excluded. Comparison of genotype accuracy was performed against 22 Taqman SNPs and demonstrated less than 0.3% discrepancy across genotyping methods. In addition, intensity cluster plots of replicated SNPs at genome-wide significant level were visually inspected for erroneous genotype assignment.

The data was examined for potential population stratification after excluding outliers detected by the IBS clustering analysis. There was no evidence for significant population stratification affecting the results.

Imputation was conducted using the algorithm implemented in MACH. To get imputed data more restrictive SNP filters including a minor allele frequency > 0.01, SNP Call Rate > 0.98, and HWE *P*-value > 1x10^-6^ were applied. In total 2,543,887 SNPs were imputed using phased haplotypes of HapMap CEU trios.

**2.2 Stage 2 cohorts**

**2.2.1 The Malmö cohorts (Malmö Diet and Cancer and the Malmö Preventive Project)** DNA extracted from peripheral blood cells was assigned to batches without regard to heart failure status, survival or personal identity. Batches were genotyped with the same set of reagents using the Sequenom iPlex assay on a MALDI-TOF mass spectrometer (MassArray, Sequenom, San Diego, CA, USA). Sequenom reagents and protocols were used with 10 ng DNA as PCR template, according to the manufacturer’s instructions. Genotype calls were obtained using MassArray Typer 4.0 software (Sequenom, San Diego, CA, USA) and fluorescence intensity plots were manually inspected and curated.

**2.2.2 Physicians’ Health Study (PHS)** DNA extracted from peripheral blood cells was assigned to batches without regard to heart failure status, survival or personal identity. Batches were genotyped with the same set of reagents using the Sequenom iPlex assay on a MALDI-TOF mass spectrometer (MassArray, Sequenom, San Diego, CA, USA). Sequenom reagents and protocols were used with 10 ng DNA as PCR template, according to the manufacturer’s instructions. Genotype calls were obtained using MassArray Typer 4.0 software (Sequenom, San Diego, CA, USA) and fluorescence intensity plots were manually inspected and curated.

**2.2.3 Prospective Study of Pravastatin in the Elderly at Risk (PROSPER)** The genome wide association study was conducted using the Illumina 660K-Quad beadchips following manufacturer’s instructions. After a stringent quality control 557,192 SNPs in 5,244 subjects were available for analysis. To maximize the availability of genetic data and coverage of the genome, imputation up to 2.5 million autosomal CEPH HapMap SNPs was performed with MACH imputation software based on the Hapmap built II release 23.

**3 In silico studies of effect on cardiac structure and function**

The association of the replicated SNPs with measures of cardiac structure and function was evaluated by lookup in summary results from the following GWA studies: EchoGen, CHARGE-HF, CHARGE-QRS, QT-IGC, and the sudden cardiac death genetics consortium.

**3.1 EchoGen**

The EchoGen study examined the association of 2.5 M genome-wide SNPs with echocardiographic phenotypes in 12,612 individuals of European ancestry from 5 community-based cohorts (Cardiovascular Health Study, Framingham Heart Study, Rotterdam Study, Gutenberg Health Study, Monica-Kora) and has been published previously [22]. Phenotypes under study include left ventricular fractional shortening, dichotomous systolic function (defined as fractional shortening < 0.29 or ejection fraction < 0.50) and cardiac dimensions (left ventricular mass, left ventricular wall thickness defined as the sum of the posterior wall thickness and the interventricular septum, left ventricular internal diastolic dimension, left atrial size, and aortic root size). The association of each SNP with each trait was analyzed in additive models adjusted for age, sex, height and weight. Effect estimates from individual studies were combined using inverse variance-weighted meta-analyses.

**3.2 CHARGE-HF**

The CHARGE-HF study examined the association of 2.5 M genome-wide SNPs with incident heart failure in 20,926 individuals from four community-based cohorts also included in the present study: ARIC, CHS, FHS and the Rotterdam Study. SNPs were in each cohort related to incident HF (n = 2,526) during a mean follow-up of 11.5 years, using Cox proportional hazards models adjusted for age and sex. Results were combined using inverse variance-weighted meta-analysis. These results from CHARGE-HF have been published previously [23].

**3.3 CHARGE-QRS**

The CHARGE-QRS study tested the association of electrocardiographic QRS duration with genome-wide SNPs in 14 cohorts, comprising 40,407 European-ancestry subjects in total. A total of ~2.5 million Hapmap II imputed SNPs were tested using a linear regression model with adjustment for age, gender, height and body mass index (and study site as appropriate). Genomic control was applied on a per-study basis. The regression results were meta-analyzed using inverse variance weighted fixed-effect models and again adjusted for the inflation factor. Details of the study populations and analytic methods have been published previously (24).

**3.4 Natriuretic Peptides in the Malmö Diet and Cancer Study**

Genotyping of genome-wide SNP data was performed in the cardiovascular cohort of the Malmö Diet and Cancer Study described above, using the Illumina Human Omni Express Bead Chip. The cardiovascular cohort has been described previously [16] and includes ~5,500 random subjects from the general population included in the study between 1991 and 1994 with blood samples available. Atrial and B-type natriuretic peptides were measured using standard assays as previously described (16). A genome-wide association study of these two biomarkers was performed (manuscript in preparation), regressing each natriuretic peptide on genome-wide SNP data adjusting for age and sex using PLINK. Additive genetic models were used. Minimal inflation of test statistics was observed, and genomic control was applied.

**3.5 QT-IGC**

The QT-IGC study tested the association of electrocardiographic QT duration with genome-wide SNPs in 31 cohorts, including a total of 76,061 subjects of European ancestry. A total of ~2.5 million SNPs imputed to HapMap II were tested using a linear regression model with adjustment for age, gender, RR interval (inverse heart rate), and principal components of genetic ancestry using an additive genetic model. Individual study results were combined using meta-analysis with inverse variance weighted fixed-effect models [25].

**3.6 CHARGE-SCD consortium**

The CHARGE-SCD consortium carried out a genome-wide association study for sudden cardiac death with 4,496 cases and over 25,000 controls of European descent from 10 cohorts: Atherosclerosis Risk in Communities study (ARIC), FinGesture, Oregon Sudden Unexpected Death Study (Oregon-SUDS), Cardiac Arrest Blood Study (CABS), CARTaGENE study, Helsinki Sudden Death Study (HSDS), Rotterdam Study, Cardiovascular Health Study (CHS), Framingham Heart Study (FHS) and Harvard cohorts (SCD cases and matched controls from the Physicians Health Study (PHS I and II), the Nurses’ Health Study (NHS), the Health Professionals Follow-up Study (HPFS), and the Women’s Antioxidant Cardiovascular Study (WACS)). The data was analyzed using a Cox proportional hazards model for prospective studies and logistic regression for case-control studies adjusting for age, sex and additional study-appropriate covariates.  Subsequent meta-analysis was performed using standard inverse-variance fixed-effects models after adjusting for appropriate genomic control factor for each study. (manuscript in preparation)

**4 Results of in vitro assessment of enhancer activity**

To experimentally test the effect of rs9885413 on enhancer activity, the 100 bp region flanking the SNP (50 bp on either side) was cloned into a reporter vector. This region includes the entire predicted NHLH1-binding motif. One construct was designed to correspond to the major allele of rs9885413 (pGL3P-G) and one to the minor allele (pGL3P-T). NHLH1 is widely expressed in human tissues, and expression was confirmed in the human embryonic kidney cell line HEK293 cells from GeoProfiles (http://www.ncbi.nlm.nih.gov/geoprofiles/) and by qPCR. The reporter vectors were transfected into HEK293 and luciferase activity was measured after 24 hours. As seen in **S4 Fig**, the signal from the pGL3P-T construct was increased 4-fold compared with pGL3P-G (*P* < 0.001), indicating that the minor allele of rs9885413 substantially increases enhancer activity.

**5 Effects of rs9885413 on gene expression**

To test effects of rs9885413 on gene expression, we first studied gene expression *in silico* in the diverse tissues from the Gene-Tissue Expression (GTEx) project [28]. No gene was significantly associated with rs9885413 after correction for multiple tests (**S8 Table**). However, these analyses were limited by a relatively small sample size, with up to 168 samples (for blood) and only 87 samples for the left ventricle.

We next assessed association of the SNP with gene expression in two large datasets with each of the tissues most relevant for the phenotype under study: heart tissue and whole blood. First, gene expression in 247 left ventricular samples from patients with advanced heart failure (n=116) undergoing transplantation and from unused donors (n=131) were profiled using Affymetrix gene expression arrays. Two of the five genes at the locus (**Fig 1**) had lower expression than background noise in both subjects with and without heart failure (*TSLP* and *CAMK4*) as shown in **S9 Table**. Of the three expressed genes, only *TMEM232* was significantly associated with the SNP rs9885413 (*P* = 2.2x10^-6^). However, another SNP at the locus (rs244412) was more strongly associated with *TMEM232* expression (*P* = 3.6x10^-19^), and the association with rs9885413 was abolished in analyses conditioning for this SNP (*P* = 0.11). The SNP rs244412 was not significantly associated with HF mortality, indicating that the *TMEM232* eQTL signal likely reflects a separate signal at the locus. Cardiac expression of *TMEM232* was also very low (**S9 Table**). Next, we tested the association of rs9885413 with the expression of genes at the locus (+/- 500 kb) in whole blood from 5257 FHS participants [29], and with DNA methylation at cg02061660 among 2262 FHS participants. All five genes at the locus except *TMEM232* were expressed in blood. Expression of one gene (*TSLP*) was significantly associated with the methylation status of cg02061660 (*P* = 1.1x10^-4^). We did not observe association of the SNP rs9885413 with any transcript.

**6 References**

1. Morrison AC, Felix JF, Cupples LA, Glazer NL, Loehr LR, Dehghan A, et al. Genomic variation associated with mortality among adults of European and African ancestry with heart failure: the cohorts for heart and aging research in genomic epidemiology consortium. Circ Cardiovasc Genet 2010; 3: 248-255.

2. Psaty BM, O'Donnell CJ, Gudnason V, Lunetta KL, Folsom AR, Rotter JI, et al. Cohorts for Heart and Aging Research in Genomic Epidemiology (CHARGE) Consortium: Design of prospective meta-analyses of genome-wide association studies from 5 cohorts. Circ Cardiovasc Genet 2009; 2: 73-80.

3. ARIC Investigators. The Atherosclerosis Risk in Communities (ARIC) Study: design

and objectives. Am J Epidemiol 1989; 129: 687-702.

4. Fried LP, Borhani NO, Enright P, Furberg CD, Gardin JM, Kronmal RA, et al. The Cardiovascular Health Study: design and rationale. Ann Epidemiol 1991; 1: 263-276.

5. Ives DG, Fitzpatrick AL, Bild DE, Psaty BM, Kuller LH, Crowley PM, et al. Surveillance and ascertainment of cardiovascular events. The Cardiovascular Health Study. Ann Epidemiol 1995; 5: 278-285.

6. Dawber T, Meadors GF, Moore FE, Epidemiologic approaches to heart disease: the Framingham study. American Journal of Public Health 1951; 41: 279-286.

7. Feinleib M, Kannel WB, Garrison RJ, The Framingham Offspring Study. Design and preliminary data. Preventive Medicine 1975; 4: 518-525.

8. Splansky GL, Corey D, Yang Q, Atwood LD, Cupples LA, Benjamin EJ, et al. The Third Generation Cohort of the National Heart, Lung, and Blood Institute's Framingham Heart Study: design, recruitment, and initial examination. American Journal of Epidemiology 2007; 165: 1328-1335.

9. Kalogeropoulos A, Georgiopoulou V, Kritchevsky SB, Psaty BM, Smith NL, Newman AB, et al. Epidemiology of incident heart failure in a contemporary elderly cohort: the health, aging, and body composition study. Arch Intern Med 2009; 169: 708-715.

10. Hofman A, Darwish Murad S, van Duijn CM, Franco OH, Goedegebure A, Ikram MA, et al. The Rotterdam Study: 2014 objectives and design update. Eur J Epidemiol 2013; 28: 889-926.

11. Remme WJ, Swedberg K. Guidelines for the diagnosis and treatment of chronic heart failure. Eur Heart J 2001; 22: 1527-1560.

12. Smith JG, Platonov PG, Hedblad B, Engstrom G, Melander O. Atrial fibrillation in the Malmo Diet and Cancer study: a study of occurrence, risk factors and diagnostic validity. Eur J Epidemiol 2010; 25: 95-102.

13. Lyssenko V, Jonsson A, Almgren P, Pulizzi N, Isomaa B, Tuomi T, et al. Clinical risk factors, DNA variants, and the development of type 2 diabetes. N Engl J Med 2008; 359: 2220-2232.

14. Ludvigsson JF, Otterblad-Olausson P, Pettersson BU, Ekbom A. The Swedish personal identity number: possibilities and pitfalls in healthcare and medical research. Eur J Epidemiol 2009; 24: 659-667.

15. Ludvigsson JF, Andersson E, Ekbom A, Feychting M, Kim JL, Reuterwall C, et al. External review and validation of the Swedish national inpatient register. BMC Public Health 2011; 11: 450.

16. Smith JG, Newton-Cheh C, Almgren P, Struck J, Morgenthaler NG, Bergmann A, et al. Assessment of conventional cardiovascular risk factors and multiple biomarkers for the prediction of incident heart failure and atrial fibrillation. J Am Coll Cardiol 2010; 56: 1712-1719.

17. Djousse L, Driver JA, Gaziano JM. Relation between modifiable lifestyle factors and lifetime risk of heart failure. JAMA 2009; 302: 394-400.

18. Lotufo PA, Gaziano JM, Chae CU, Ajani UA, Moreno-John G, Buring JE, et al. Diabetes and all-cause and coronary heart disease mortality among US male physicians. Arch Intern Med 2001; 161: 242-247.

19. Shepherd J, Blauw GJ, Murphy MB, Bollen EL, Buckley BM, Cobbe SM, et al. Pravastatin in elderly individuals at risk of vascular disease (PROSPER): a randomised controlled trial. Lancet 2002; 360: 1623-1630.

20. Shepherd J, Blauw GJ, Murphy MB, Cobbe SM, Bollen EL, Buckley BM, et al. The design of a prospective study of Pravastatin in the Elderly at Risk (PROSPER). PROSPER Study Group. PROspective Study of Pravastatin in the Elderly at Risk. Am J Cardiol 1999; 84: 1192-1197.

21. Price AL, Patterson NJ, Plenge RM, Weinblatt ME, Shadick NA, Reich D. Principal components analysis corrects for stratification in genome-wide association studies. Nat Genet 2006; 38: 904-909.

22. Vasan RS, Glazer NL, Felix JF, Lieb W, Wild PS, Felix SB, et al. Genetic variants associated with cardiac structure and function: a meta-analysis and replication of genome-wide association data. JAMA 2009; 302: 168-178.

23. Smith NL, Felix JF, Morrison AC, Demissie S, Glazer NL, Loehr LR, et al. Association of genome-wide variation with the risk of incident heart failure in adults of European and African ancestry: a prospective meta-analysis from the cohorts for heart and aging research in genomic epidemiology (CHARGE) consortium. Circ Cardiovasc Genet 2010; 3: 256-266.

24. Sotoodehnia N, Isaacs A, de Bakker PI, Dorr M, Newton-Cheh C, Nolte IM, et al. Common variants in 22 loci are associated with QRS duration and cardiac ventricular conduction. Nat Genet 2010; 42: 1068-1076.

25. Arking DE, Pulit SL, Crotti L, van der Harst P, Munroe PB, Koopmann TT, et al. Genetic association study of QT interval highlights role for calcium signaling pathways in myocardial repolarization. Nat Genet 2014; 46: 826-836.

26. Kannel WB, Feinleib M, McNamara PM, Garrison RJ, Castelli WP. An investigation of coronary heart disease in families. The Framingham offspring study. Am J Epidemiol 1979; 110: 281-90.

**7 Acknowledgements**

The authors would like to gratefully acknowledge all study subjects and the following consortia who contributed summary results for the SNP on chromosome 5q22 from genome-wide association datasets of phenotypes reflecting cardiac structure and function. The authors also wish to acknowledge Gulum Kosova for assistance with population genetic analyses.

**The CHARGE-QRS consortium**

The following individuals contributed to the CHARGE-QRS consortium:

Nona Sotoodehnia1,2,78, Aaron Isaacs3,4,78, Paul I W de Bakker5–8,78, Marcus Dörr9,78, Christopher Newton-Cheh10–12,78, Ilja M Nolte13,78, Pim van der Harst14,78, Martina Müller15–17,78, Mark Eijgelsheim18,78, Alvaro Alonso19,78, Andrew A Hicks20,78, Sandosh Padmanabhan21,78, Caroline Hayward22,78, Albert Vernon Smith23,24,78, Ozren Polasek25,78, Steven Giovannone26,78, Jingyuan Fu13,27,78, Jared W Magnani12,28, Kristin D Marciante2, Arne Pfeufer20,29,30, Sina A Gharib31, Alexander Teumer32, Man Li33, Joshua C Bis2, Fernando Rivadeneira18,34, Thor Aspelund23,24, Anna Köttgen35, Toby Johnson36,37, Kenneth Rice38, Mark P S Sie3, Ying A Wang12,39, Norman Klopp17,

Christian Fuchsberger20, Sarah H Wild40, Irene Mateo Leach14, Karol Estrada34, Uwe Völker32, Alan F Wright22, Folkert W Asselbergs13,14,41, Jiaxiang Qu26, Aravinda Chakravarti42, Moritz F Sinner16, Jan A Kors43, Astrid Petersmann44, Tamara B Harris45, Elsayed Z Soliman46, Patricia B Munroe36,37, Bruce M Psaty2,47–49, Ben A Oostra4,50, L Adrienne Cupples12,39, Siegfried Perz51, Rudolf A de Boer14, André G Uitterlinden18,34,52, Henry Völzke53, Timothy D Spector54, Fang-Yu Liu26, Eric Boerwinkle55,56, Anna F Dominiczak21, Jerome I Rotter57, Gé van Herpen43, Daniel Levy12,58, H-Erich Wichmann15,17,59, Wiek H van Gilst14, Jacqueline C M Witteman18,52, Heyo K Kroemer60, W H Linda Kao33, Susan R Heckbert2,47,49, Thomas Meitinger29,30, Albert Hofman18,52, Harry Campbell40, Aaron R Folsom19, Dirk J van Veldhuisen14, Christine Schwienbacher20,61, Christopher J O’Donnell12,58, Claudia Beu Volpato20, Mark J Caulfield36,37, John M Connell62, Lenore Launer45, Xiaowen Lu13, Lude Franke27,63, Rudolf S N Fehrmann27, Gerard te Meerman27, Harry J M Groen64, Rinse K Weersma65, Leonard H van den Berg66, Cisca Wijmenga27, Roel A Ophoff 67,68, Gerjan Navis69, Igor Rudan40,70,71,78, Harold Snieder13,54,78, James F Wilson40,78, Peter P Pramstaller20,72,73,78, David S Siscovick2,47,78, Thomas J Wang11,12,78, Vilmundur Gudnason23,24,78, Cornelia M van Duijn3,4,52,78, Stephan B Felix9,78, Glenn I Fishman26,78, Yalda Jamshidi54,74,78, Bruno H Ch Stricker18,34,43,52,75,78, Nilesh J Samani76–78, Stefan Kääb16,78 & Dan E Arking42,78

Affiliations

1 Division of Cardiology, Department of Medicine, University of Washington, Seattle, Washington, USA.

2 Cardiovascular Health Research Unit, Department of Medicine, University of Washington, Seattle, Washington, USA.

3 Genetic Epidemiology Unit, Department of Epidemiology, Erasmus Medical Center (MC), Rotterdam, The Netherlands.

4 Centre for Medical Systems Biology, Leiden, The Netherlands.

5 Division of Genetics, Department of Medicine, Brigham and Women’s Hospital, Harvard Medical School, Boston, Massachusetts, USA.

6 Program in Medical and Population Genetics, Broad Institute, Cambridge, Massachusetts, USA.

7 Department of Medical Genetics, University Medical Center, Utrecht, The Netherlands. 8Julius Center for Health Sciences and Primary Care, University Medical Center, Utrecht, The Netherlands.

9 Department of Internal Medicine B, Ernst Moritz Arndt University Greifswald, Greifswald, Germany.

10 Center for Human Genetic Research, Massachusetts General Hospital, Boston, Massachusetts, USA.

11 Cardiology Division, Massachusetts General Hospital, Boston, Massachusetts, USA.

12 National Heart, Lung, and Blood Institute’s (NHLBI) Framingham Heart Study, Framingham, Massachusetts, USA.

13 Unit of Genetic Epidemiology and Bioinformatics, Department of Epidemiology, University Medical Center Groningen, University of Groningen, Groningen, The Netherlands.

14 Department of Cardiology, University Medical Center Groningen, University of Groningen, The Netherlands.

15 Institute of Medical Informatics, Biometry and Epidemiology, Chair of Epidemiology, Ludwig-Maximilians-Universität, Munich, Germany.

16 Department of Medicine I, University Hospital Grosshadern, Ludwig-Maximilians-Universität, Munich, Germany.

17 Institute of Epidemiology, Helmholtz Zentrum München-German Research Center for Environmental Health, Neuherberg, Germany.

18 Department of Epidemiology, Erasmus MC, Rotterdam, The Netherlands.

19 Division of Epidemiology and Community Health, School of Public Health, University of Minnesota, Minneapolis, Minnesota, USA.

20 Institute of Genetic Medicine, European Academy Bozen-Bolzano (EURAC), Bolzano, Italy, affiliated institute of the University of Lübeck, Germany.

21 Institute of Cardiovascular and Medical Sciences, College of Medical, Veterinary and Life Sciences, University of Glasgow, University Place, Glasgow, UK.

22 Medical Research Council (MRC) Human Genetics Unit, Institute of Genetics and Molecular Medicine, Edinburgh, UK.

23 Icelandic Heart Association, Kopavogur, Iceland.

24 University of Iceland, Reykjavik, Iceland.

25 Andrija Stampar School of Public Health, Medical School, University of Zagreb, Zagreb, Croatia.

26 Leon H. Charney Division of Cardiology, New York University School of Medicine, New York, New York, USA.

27 Department of Genetics, University Medical Center Groningen, University of Groningen, The Netherlands.

28 Section of Cardiovascular Medicine, Boston University School of Medicine, Boston, Massachusetts, USA.

29 Institute of Human Genetics, Helmholtz Zentrum München-German Research Center for Environmental Health, Neuherberg, Germany. 30 Institute of Human Genetics, Klinikum Rechts der Isar, Technische Universität München, Munich, Germany.

31 Center for Lung Biology, Department of Medicine, University of Washington, Seattle, Washington, USA.

32 Interfaculty Institute for Genetics and Functional Genomics, Ernst Moritz Arndt University Greifswald, Greifswald, Germany. 33 Department of Epidemiology and the Welch Center for Prevention, Epidemiology and Clinical Research, Johns Hopkins University, Baltimore, Maryland, USA.

34 Department of Internal Medicine, Erasmus MC, Rotterdam, The Netherlands.

35 Department of Epidemiology, Johns Hopkins University, Baltimore, Maryland, USA.

36 Clinical Pharmacology and Barts and the London Genome Centre, William Harvey Research Institute, Barts and the London School of Medicine, Queen Mary University of London, London, UK.

37 Barts and the London National Institute of Health Research Cardiovascular Biomedical Research Unit, London, UK.

38 Department of Biostatistics, University of Washington, Seattle, Washington, USA.

39 Department of Biostatistics, Boston University School of Public Health, Boston, Massachusetts, USA.

40 Centre for Population Health Sciences, University of Edinburgh, Edinburgh, Scotland.

41 Department of Cardiology, Division of Heart and Lungs, University Medical Center Utrecht, Utrecht, The Netherlands.

42 McKusick-Nathans Institute of Genetic Medicine, Johns Hopkins University School of Medicine, Baltimore, Maryland, USA.

43 Department of Medical Informatics, Erasmus MC, Rotterdam, The Netherlands.

44 Institute of Clinical Chemistry and Laboratory Medicine, Ernst Moritz Arndt University Greifswald, Greifswald, Germany.

45 Laboratory of Epidemiology, Demography and Biometry, National Institute on Aging, National Institutes of Health, Bethesda, Maryland, USA.

46 Epidemiological Cardiology Research Center (EPICARE), Wake Forest University School of Medicine, Winston Salem, North Carolina, USA.

47 Department of Epidemiology, University of Washington, Seattle, Washington, USA.

48 Department of Health Services, University of Washington, Seattle, Washington, USA.

49 Group Health Research Institute, Group Health Cooperative, Seattle, Washington, USA.

50 Department of Clinical Genetics, Erasmus MC, Rotterdam, The Netherlands.

51 Institute for Biological and Medical Imaging, Helmholtz Zentrum München-German Research Center for Environmental Health, Neuherberg, Germany.

52 Netherlands Genomics Initiative (NGI)-sponsored Netherlands Consortium for Healthy Aging (NCHA), Rotterdam, The Netherlands.

53 Institute for Community Medicine, Ernst Moritz Arndt University Greifswald, Greifswald, Germany.

54 Department of Twin Research and Genetic Epidemiology Unit, St. Thomas’ Campus, King’s College London, St. Thomas’ Hospital, London, UK.

55 Human Genetics Center, University of Texas Health Science Center at Houston, Houston, Texas, USA.

56 Institute for Molecular Medicine, University of Texas Health Science Center at Houston, Houston, Texas, USA.

57 Medical Genetics Institute, Cedars-Sinai Medical Center, Los Angeles, California, USA.

58 National Heart, Lung, and Blood Institute, Bethesda, Maryland, USA.

59 Klinikum Grosshadern, Munich, Germany.

60 Department of Pharmacology, Center for Pharmacology and Experimental Therapeutics, Ernst Moritz Arndt University Greifswald, Greifswald, Germany.

61 Department of Experimental and Diagnostic Medicine, University of Ferrara, Ferrara, Italy.

62 University of Dundee, Ninewells Hospital and Medical School, Dundee, UK.

63 Blizard Institute of Cell and Molecular Science, Barts and The London School of Medicine and Dentistry, Queen Mary University of London, London, UK.

64 Department of Pulmonology, University Medical Center Groningen, University of Groningen, Groningen, The Netherlands.

65 Department of Gastroenterology and Hepatology, University Medical Center Groningen, University of Groningen, Groningen, The Netherlands.

66 Department of Neurology, Rudolf Magnus Institute, University Medical Center Utrecht, University of Utrecht, Utrecht, The Netherlands. 67 Department of Medical Genetics and Rudolf Magnus Institute, University Medical Center Utrecht, Utrecht, The Netherlands.

68 Center for Neurobehavioral Genetics, University of California, Los Angeles, California, USA.

69 Department of Internal Medicine, University Medical Center Groningen, University of Groningen, Groningen, The Netherlands.

70 Centre for Global Health, Medical School, University of Split, Split, Croatia.

71 Gen-info Ltd. Zagreb, Croatia.

72 Department of Neurology, General Central Hospital, Bolzano, Italy.

73 Department of Neurology, University of Lübeck, Lübeck, Germany.

74 Division of Clinical Developmental Sciences, St. George’s University of London, London, UK.

75 Inspectorate of Health Care, The Hague, The Netherlands.

76 Department of Cardiovascular Sciences, University of Leicester, Leicester, UK.

77 Leicester NIHR Biomedical Research Unit in Cardiovascular Disease Glenfield Hospital, Leicester, UK.

78 These authors contributed equally to this work.

**The CHARGE-SCD consortium**

The following studies and individuals contributed to the CHARGE-SCD consortium:

*Atherosclerosis Risk in Communities study (ARIC)*: Dan Arking, Foram Ashar, Aravinda Charkravarti (Department of Genetic Medicine, Johns Hopkins), Eric Boerwinkle (Department of Epidemiology, University of Texas)

*FINGESTURE*: Jean-Claude Tardif (Montreal Heart Institute, Université de Montréal, Montreal CA),John D. Rioux, Phillipe Goyette (Université de Montréal & Montreal Heart Institute, Montreal CA), Juhani Jinttila, Heikki Huikuri (Dept. of Internal Medicine, University of Oulu and University Central Hospital, Oulu, Finland), Marja-Leena Kortelainen (Department of Forensic Medicine, University of Oulu, Oulu Finland)

*OregonSUDS*: Adriana Huertas-Vazquez, Kyndaron Reinier, Carmen Teodorescu, Audrey Uy-Evanado, Sumeet S. Chugh (The Heart Institute, Cedars-Sinai Medical Center, Los Angeles CA)

*Cardiac Arrest Blood Study (CABS)*: Rozenn Lemaitre, Catherine Johnson (Cardiovascular Health Research Unit, Department of Medicine, University of Washington, Seattle WA), David Siscovick (Cardiovascular Health Research Unit, Departments of Medicine and Epidemiology, University of Washington, Seattle WA), Angel Mak, Stephanie E. Hesselson, Pui-Yan Kwok (Department of Dermatology, Cardiovascular Research Institute, and Institute for Human Genetics, University of California, San Francisco, CA)

*CARTAGENE*: Martina Muller-Nurasyid (Department of Medicine I, University Hospital Grosshadern, Ludwig-Maximilians-Universität, Munich, Germany. 2. Institute of Medical Informatics, Biometry and Epidemiology, Chair of Genetic Epidemiology, Ludwig-Maximilians-Universität, Munich, Germany. 3. Institute of Genetic Epidemiology, Helmholtz Zentrum München – German Research Center for Environmental Health, Neuherberg, Germany), Stefan Kaab (Department of Medicine I, University Hospital Grosshadern, Ludwig-Maximilians-Universität, Munich, Germany. 2. DZHK (German Centre for Cardiovascular Research), partner site Munich Heart Alliance, Munich, Germany), Xavier Jouven (Departments of cardiology and epidemiology, University Paris Descartes, Paris , France)

*Helsinki Sudden Death Study (HSDS)*: Leo-Pekka Lyytikäinen, Terho Lehtimäki (Department of Clinical Chemistry, Fimlab Laboratories and University of Tampere School of Medicine, Tampere, Finland), Pekka J. Karhunen (Department of Forensic Medicine and Clinical Pathology, Fimlab laboratories,; University of Tampere School of Medicine, and University of Eastern Finland, Tampere, Finland)

*Rotterdam Study (RS)*: Bouwe Krijthe, Oscar Franco, Albert Hofman (Department of Epidemiology, Erasmus MC, Rotterdam, The Netherlands), Mark Eigelsheim, Andre G Uitterlinden, Bruno Stricker (Dept Epidemiology and Dept Internal Medicine, Erasmus Medical Center Rotterdam, The Netherlands)

*Cardiovascular Health Study (CHS)*: Nona Sotoodehnia (Cardiovascular Health Research Unit, Division of Cardiology, University of Washington, Seattle WA), Jennifer Brody, Colleen Sitlani (Cardiovascular Health Research Unit, Department of Medicine, University of Washington, Seattle WA), Bruce Psaty (Cardiovascular Health Research Unit, Departments of Medicine, Epidemiology and Health Services, University of Washington, and Group Health Research Institute, Group Health Cooperative, Seattle, WA), Jerome Rotter (Departments of Pediatrics, Medicine, and Human Genetics, UCLA), Barbara McKnight (Cardiovascular Health Research Unit, Department of Biostatistics, University of Washington, Seattle WA)

*Framingham Heart Study (FHS)*: Christopher Newton-Cheh (Center for Human Genetic Research and Cardiology Division, Massachusetts General Hospital, Boston, MA and Framingham Heart Study, National Heart, Lung, and Blood Institute, National Institutes of Health, Framingham, MA, USA;), L. Adrienne Cupples (Department of Biostatistics, Boston University School of Public Health, Boston MA)

*Harvard Cohorts Study of Sudden Death*: Christine Albert (Department of Medicine, Brigham and Women's Hospital, Boston, MA), Sara L. Pulit (Program in Medical and Population Genetics, Broad Institute of MIT and Harvard, Cambridge, MA), Christopher Newton-Cheh (Center for Human Genetic Research and Cardiology Division, Massachusetts General Hospital, Boston, MA and Framingham Heart Study, National Heart, Lung, and Blood Institute, National Institutes of Health, Framingham, MA, USA;)

**The EchoGen consortium**

The following studies and individuals contributed to the EchoGen consortium:

*Framingham Heart Study*: National Heart, Lung, and Blood Institute’s Framingham Heart Study, Framingham (Drs Ramachandran S. Vasan, Martin G. Larson, Sekar Kathiresan, Jayashri Aragam, Daniel Levy, Christopher J. O’Donnell, Gary F. Mitchell, Thomas J. Wang, and Emelia J. Benjamin); Departments of Medicine, Preventive Medicine and Cardiology Sections, Boston University School of Medicine (Drs Ramachandran S. Vasan and Emelia J. Benjamin) and Department of Mathematics and Statistics, Boston University (Dr Martin G. Larson), Boston, Massachusetts; and National Heart, Lung, and Blood Institute (Drs Christopher J. O’Donnell and Daniel Levy), Bethesda, Maryland.

*The Cardiovascular Health Study*: Cardiovascular Health Research Unit and Department of Medicine (Drs Nicole L. Glazer, Joshua C. Bis, and Bruce M. Psaty), Departments of Biostatistics (Drs Thomas Lumley and Kenneth Rice), Epidemiology (Drs Susan R. Heckbert, Nicholas L. Smith, and Bruce M. Psaty), and Health Services (Dr Bruce M. Psaty), University of Washington, Seattle; Seattle Epidemiologic Research and Information Center of the Department of Veterans Affairs Office of Research and Development (Dr Nicholas L. Smith) and Center for Health Studies, Group Health (Dr Bruce M. Psaty), Seattle; Department of Epidemiology, University of Alabama at Birmingham (Dr Donna K. Arnett); Division of Cardiology, University of Maryland Hospital, Baltimore (Dr John S. Gottdiener); and Medical Genetics Institute, Cedars-Sinai Medical Center, West Los Angeles, California (Drs Talin Haritunians and Jerome I. Rotter).

*Rotterdam Study*: Departments of Epidemiology (Drs Janine F. Felix, Abbas Dehghan, Yurii S. Aulchenko, Maksim Struchalin, Bruno H. Stricker, Albert Hofman, Cornelia M. van Duijn, and Jacqueline C. Witteman), Internal Medicine (Drs Fernando Rivadeneira and Andre G. Uitterlinden), Cardiology (Dr Jaap W. Deckers), Erasmus MC, University Medical Center Rotterdam, Rotterdam, the Netherlands; Netherlands Consortium on Healthy Aging (Drs Janine F. Felix, Andre G. Uitterlinden and Jacqueline C. Witteman).

*MONICA/KORA*: Medical Clinic 2 (Drs Wolfgang Lieb, Anika Großhennig, Jeanette Erdmann, Jan Stritzke, and Heribert Schunkert) and Institute of Medical Biometry and Statistics (Drs Anika Großhennig, Inke R. König), University of Lübeck, Lübeck; Institutes of Epidemiology (Dr H-Erich Wichmann) and Human Genetics (Dr Thomas Meitinger), Helmholtz Zentrum München, München; German Research Center for Environmental Health, Neuherberg and Ludwig Maximilians University (Dr H-Erich Wichmann) and German Research Center for Environmental Health, Neuherberg, Technische Universität München (Dr Thomas Meitinger), Munich, Germany.

*Gutenberg Heart Study:* Departments of Medicine II (Drs Philipp S. Wild, Tanja Zeller, Renate B. Schnabel, Thomas F. Münzel, and Stefan Blankenberg), Clinical Chemistry and Laboratory Medicine (Dr Karl J. Lackner), Institute of Medical Biometry, Epidemiology, and Informatics (Dr Maria Blettner), Johannes Gutenberg-University, Mainz, and Institute forMedical Biometry and Statistics (Drs Arne Schillert and Andreas Ziegler), University Lübeck, Germany.

*Study of Health in Pomerania:* Department of Internal Medicine B (Drs Stephan B. Felix, Marcus Dörr, and Thorsten Reffelmann), Interfaculty Institute for Genetics and Functional Genomics (Drs Alexander Teumer, Georg Homuth, and Uwe Völker), Institute of Pharmacology (Dr Heyo K. Kroemer), and Institute for Community Medicine (Drs Nele Friedrich and Henry Völzke), Ernst-Moritz-Arndt-Universität, Greifswald, Germany (Drs Stephan B. Felix, Georg Homuth, Marcus Dörr, Uwe Völker, Thorsten Reffelmann, Nele Friedrich, Heyo K. Kroemer, and Henry Völzke and Alexander Teumer).

*Austrian Stroke Prevention Study:* Department of Internal Medicine, Division of Cardiology (Drs Norbert Watzinger and Robert Zweiker), Department of Neurology (Dr Reinhold Schmidt), and Institute for Molecular Biology and Biochemistry (Dr Helena Schmidt), Medical University Graz, Graz, Austria.

*PIVUS Study:* Department of Medical Sciences, Uppsala University, Uppsala (Dr Lars Lind) and Department of Medical Epidemiology and Biostatistics, Karolinska Institute, Stockholm (Dr Erik Ingelsson). Division of Cardiovascular Diseases, Mayo Clinic, Rochester, Minnesota (Drs Richard J. Rodeheffer, Iftikhar Kullo, and Margaret M. Redfield).

*CARLA Study*: Institute of Medical Epidemiology, Biostatistics and Informatics (Drs Karin Halina Greiser and Johannes Haerting), Germany; and Center for Population Studies, National Heart, Lung, and Blood Institute, Bethesda, Maryland (Dr Daniel Levy).

**The QT-IGC consortium**

The following individuals contributed to the QT-IGC consortium:

Dan E. Arking^1^*, Sara Pulit^2-4^*, Lia Crotti^5-7^, Pim van der Harst^8,9^, Patricia B. Munroe^10^, Tamara T. Koopmann^11^, Nona Sotoodehnia^12,13^, Elizabeth J. Rossin^4,14,15^, Andrew D. Johnson^16^, Alicia Lundby^4,17,18^, Daníel F. Gudbjartsson^19^, Peter A. Noseworthy^2-4^, Mark Eijgelsheim^20^, Yuki Bradford^21^, Kirill V. Tarasov^22^, Marcus Dörr^23^, Martina Mueller-Nurasyid^24-26^, Annukka M. Lahtinen^27^, Ilja M. Nolte^28^, Albert Vernon Smith^29,30^, Joshua C. Bis^12^, Aaron Isaacs^31^, Stephen J. Newhouse^10^, Daniel S. Evans^32^, Wendy S. Post^33,34^, Daryl Waggot^35^, Leo-Pekka Lyytikäinen^36^, Andrew A. Hicks^37^, Lewin Eisele^38^, David Ellinghaus^39^, Caroline Hayward^40^, Pau Navarro^40^, Sheila Ulivi^41^, Toshiko Tanaka^42^, David J. Tester^43^, Stéphanie Chatel^44,45^, Stefan Gustafsson^46^, Meena Kumari^47^, Richard W. Morris^48^, Åsa T. Naluai^49^, Sandosh Padmanabhan^50^, Alexander Kluttig^51^, Bernhard Strohmer^52^, Andrie G. Panayiotou^53,54^, Maria Torres^55^, Michael Knoflach^56^, Jaroslav A. Hubacek^57^, Kamil Slowikowski^58,59^, Soumya Raychaudhuri^4,58,60-62^, Tamara B. Harris^63^, Lenore J. Launer^63^, Alan Shuldiner^64-66^, Alvaro Alonso^67^, Joel S. Bader^68^, Georg Ehret^1^, Hailiang Huang^68^, W.H. Linda Kao^34^, James B. Strait^22,42^, Peter W. MacFarlane^69^, Morris Brown^70^, Mark J. Caulfield^10^, Nilesh J. Samani^71^, Johann Willeit^56^, CARe Consortium, COGENT Consortium, J. Gustav Smith^2,3,72^, Karin H. Greiser^51,73^, Henriette Meyer zu Schwabedissen^74^, Karl Werdan^75^, Massimo Carella^76^, Leopoldo Zelante^76^, Susan R. Heckbert^12^, Bruce Psaty^12,77,78^, Jerome I. Rotter^79^, Ivana Kolcic^80^, Ozren Polašek^80^, Alan F. Wright^40^, Maura Griffin^81^, Mark J. Daly^4,14^, DCCT/EDIC, David O. Arnar^82^, Hilma Hólm^19^, Unnur Thorsteinsdottir^19^, eMERGE consortium, Joshua C. Denny^83^, Dan M. Roden^84^, Rebecca L. Zuvich^21^, Valur Emilsson^29^, Andrew S. Plump^85^, Martin G. Larson^16,86,87^, Christopher J. O'Donnell^16,88^, Xiaoyan Yin^16,86^, Marco Bobbo^89^, Adamo P. D'Adamo^41,90^, Annamaria Iorio^89^, Gianfranco Sinagra^89^, Angel Carracedo^55,91^, Steven R. Cummings^32^, Michael A. Nalls^92^, Antti Jula^93^, Kimmo Kontula^94^, Annukka M. Marjamaa^27^, Lasse Oikarinen^95^, Markus Perola^96-98^, Kimmo Porthan^95^, Raimund Erbel^99^, Per Hoffmann^100,101^, Karl-Heinz Jöckel^38^, Hagen Kälsch^99^, Markus Nöthen^100,101^, HRGEN consortium, Marcel den Hoed^102^, Ruth J.F. Loos^102,103^, Dag S. Thelle^104^, Christian Gieger^26^, Thomas Meitinger^105-107^, Siegfried Perz^108^, Annette Peters^109,110^, Hanna Prucha^105,111,112^, Moritz F. Sinner^3,24^, Melanie Waldenberger^106,113^, Rudolf A. de Boer^9^, Lude Franke^9^, Pieter A. van der Vleuten^8,9^, Britt Maria Beckmann^24^, Eimo Martens^24^, Abdennasser Bardai^11^, Nynke Hofman^114^, Arthur A.M. Wilde^11^, Elijah R. Behr^115^, Chrysoula Dalageorgou^116^, John R. Giudicessi^43^, Argelia Medeiros-Domingo^43^, Julien Barc^45^, Florence Kyndt^44,45^, Vincent Probst^44,45^, Alice Ghidoni^5,6^, Roberto Insolia^5,6^, Robert M. Hamilton^117^, Stephen W. Scherer^118^, Fabiola Del Greco M.^37^, Christian Fuchsberger^119^, Jeffrey R. O'Connell^64,65^, Wai K. Lee^50^, Graham C.M. Watt^120^, Harry Campbell^121^, Sarah H. Wild^121^, Nour E. El Mokhtari^122^, Norbert Frey^123^, Folkert W. Asselbergs^8^, Irene Mateo Leach^8^, Gerjan Navis^124^, Maarten P. van den Berg^8^, Dirk J. van Veldhuisen^8^, Bouwe P. Krijthe^20,125^, Oscar H. Franco^20,125^, Albert Hofman^20,125^, Jan A. Kors^126^, André G. Uitterlinden^20,125,127^, Jacqueline C.M. Witteman^20,125^, Ludmilla Kedenko^128^, Ben A. Oostra^20^, Gonçalo R. Abecasis^119^, Edward G. Lakatta^22^, Antonella Mullas^129^, Marco Orrú^129^, David Schlessinger^130^, Manuela Uda^129^, Marcello R.P. Markus^131^, Uwe Völker^132^, Harold Snieder^28^, Timothy D. Spector^133^, Johan Ärnlöv^134^, Lars Lind^135^, Johan Sundström^135^, Ann-Christine Syvänen^136^, Mika Kivimaki^47^, Mika Kähönen^137^, Nina Mononen^36^, Olli T. Raitakari^138^, Jorma Viikari^139^, Vera Adamkova^57^, Stefan Kiechl^56^, Maria Brion^55,140^, Andrew N. Nicolaides^54,81^, Bernhard Paulweber^128^, Johannes Haerting^51^, Anna F. Dominiczak^50^, Fredrik Nyberg^141,142^, Peter H. Whincup^143^, Aroon Hingorani^47^, Jean-Jacques Schott^45^, Connie R. Bezzina^11^, Erik Ingelsson^46^, Luigi Ferrucci^42^, Paolo Gasparini^41,90^, James F. Wilson^121^, Igor Rudan^121^, Andre Franke^39^, Thomas W. Mühleisen^100,101^, Peter P. Pramstaller^37,144,145^, Terho J. Lehtimäki^36^, Andrew D. Paterson^146^, Afshin Parsa^64,65^, Yongmei Liu^147^, Cornelia van Duijn^20^, David S. Siscovick^12,77,148^, Vilmundur Gudnason^29,30^, Yalda Jamshidi^149^, Veikko Salomaa^93^, Stephan B. Felix^23^, Serena Sanna^129^, Marylyn D. Ritchie^150^, Bruno H. Stricker^20,125-127,151^, Kari Stefansson^19,30^, Kasper Lage^4,14,17,152,153^, Jesper V. Olsen^17^, Peter J. Schwartz^5,6,154-156^, Stefan Kääb^24,110^, Aravinda Chakravarti^1^, Michael J. Ackerman^43^*, Arne Pfeufer^37,105,157^*, Paul I.W. de Bakker^4,15,58^*, Christopher Newton-Cheh^2-4,15^*

Affiliations

1 Center for Complex Disease Genomics, McKusick-Nathans Institute of Genetic Medicine, Johns Hopkins University School of Medicine,

Baltimore, MD 21205, USA.

2 Center for Human Genetic Research, Massachusetts General Hospital, Boston, MA 02114, USA.

3 Cardiovascular Research Center, Massachusetts General Hospital, Boston, MA, USA 02114.

4 Program in Medical and Population Genetics, Broad Institute of Harvard and MIT, Cambridge, MA 02139, USA.

5 Department of Molecular Medicine, Section of Cardiology, University of Pavia, Pavia, Italy.

6 Department of Cardiology, Fondazione IRCCS Policlinico S. Matteo, Pavia, Italy.

7 Institute of Human Genetics, Helmholtz Center Munich, Germany.

8 Department of Cardiology, University of Groningen, University Medical Center Groningen, Groningen, The Netherlands.

9 Department of Genetics, University of Groningen, University Medical Center Groningen, 9700 RB Groningen, The Netherlands.

10 Clinical Pharmacology and Barts and the London Genome,Centre, William Harvey Research Institute, Barts and the London School of Medicine, Queen Mary University of London, London EC1M 6BQ, UK.

11 Heart Failure Research Center, Department of Clinical and Experimental Cardiology, Academic Medical Center, Amsterdam, The Netherlands.

12 Cardiovascular Health Research Unit, Department of Medicine, University of Washington, Seattle, USA.

13 Cardiology Division, University of Washington, Seattle, WA, USA.

14 Analytic and Translational Genetics Unit, Massachusetts General Hospital, Boston, MA, 02114, USA.

15 Harvard Medical School, Boston, MA 02115, USA.

16 NHLBI's Framingham Heart Study, Framingham, MA, 01702, USA.

17 Novo Nordisk Foundation Center for Protein Research, Faculty of Health Sciences, University of Copenhagen, Blegdamsvej 3b, Copenhagen, Denmark.

18 The Danish National Research Foundation Centre for Cardiac Arrhythmia, University of Copenhagen, Blegdamsvej 3b, DK-2200 Copenhagen, Denmark.

19 deCODE genetics, Sturlugata 8, 101 Reykjavik, Iceland.

20 Department of Epidemiology, Erasmus Medical Center, P.O. Box 2040, 3000 CA, Rotterdam, The Netherlands.

21 Center for Human Genetics Research , Vanderbilt University School of Medicine, Nashville, TN, 37232, USA.

22 Laboratory of Cardiovascular Sciences, Human Cardiovascular Studies Unit, National Institute on Aging, National Institutes of Health, Baltimore, Maryland, 21224.

23 Department of Internal Medicine B, University Medicine Greifswald, 17475 Greifswald, Germany.

24 Department of Medicine I, University Hospital Grosshadern, Ludwig-Maximilians University, Munich, Germany.

25 Institute of Medical Informatics, Biometry and Epidemiology, Chair of Epidemiology and Chair of Genetic Epidemiology, Ludwig-Maximilians-Universität, Munich, Germany.

26 Institute of Genetic Epidemiology, Helmholtz Zentrum München Germany.

27 Research Programs Unit, Molecular Medicine, University of Helsinki, PO Box 63FI-00014, Helsinki, Finland.

28 Unit of Genetic Epidemiology & Bioinformatics, Department of Epidemiology, University Medical Center Groningen, University of Groningen, 9700 RB Groningen, the Netherlands.

29 Icelandic Heart Association, Holtasmari 1, IS-201 Kopavogur, Iceland.

30 Faculty of Medicine, University of Iceland, 101 Reykjavik, Iceland.

31 Genetic Epidemiology Unit, Dept. of Epidemiology, Erasmus University Medical Center, 300CA Rotterdam, the Netherlands.

32 California Pacific Medical Center Research Institute, San Francisco, CA 94107, USA.

33 Division of Cardiology, Johns Hopkins University School of Medicine, Baltimore, MD, 21205, USA.

34 Department of Epidemiology, Johns Hopkins University, Bloomberg School of Public Health, Baltimore, MD, 21205, USA.

35 Informatics and Biocomputing Platform, Ontario Institute for Cancer Research, Toronto, Canada, M5G 0A3.

36 Department of Clinical Chemistry, Fimlab Laboratories, University of Tampere, Tampere University Hospital, PO Box 2000, 33521 Tampere, Finland.

37 Center for Biomedicine, European Academy Bozen/Bolzano (EURAC), Bolzano, Italy - Affiliated Institute of the University of Lübeck, Lübeck, Germany.

38 Institute for Medical Informatics, Biometry and Epidemiology, University Hospital of Essen, University Duisburg-Essen, Essen, Germany.

39 Institute of Clinical Molecular Biology, Christian-Albrechts-University Campus Kiel, Niemannsweg 11, House 1, D-24105 Kiel, Germany.

40 MRC Human Genetics Unit, Institute of Genetics and Molecular Medicine,Western General Hospital, Edinburgh, EH4 2XU, United Kingdom.

41 Institute for Maternal and Child Health - IRCCS “Burlo Garofolo” – Trieste, Trieste, Italy, 34137.

42 Clinical Research Branch, National Institute on Aging, Baltimore, MD, 21225, USA.

43 Departments of Medicine, Pediatrics, and Molecular Pharmacology & Experimental Therapeuticis; Divisions of Cardiovascular Diseases and Pediatric Cardiology; Windland Smith Rice Sudden Death Genomics Laboratory; Mayo Clinic, Rochester, MN, USA  55905.

44 l'institut du thorax, CHU de Nantes, Université de Nantes, Nantes, France, F-44000.

45 l'institut du thorax, INSERM UMR1087, CNRS UMR 6291, Université de Nantes, Nantes, France, F-44000.

46 Department of Medical Epidemiology and Biostatistics, Karolinska Institutet, Box 281, SE-171 77 Stockholm, Sweden.

47 Department of Epidemiology and Public Health, University College London, 1-19 Torrington Place, London, WC1E 6BT.

48 University College London, Dept of Primary Care & Population Health, Royal Free Campus, London NW3 2PF, UK.

49 Department of Medical and Clinical Genetics, University of Gothenburg, 413 45 Gothenburg, Sweden.

50 BHF Glasgow Cardiovascular Research Centre, Institute of Cardiovascular and Medical Sciences, College of Medical, Veterinary and Life Sciences, University of Glasgow, G12 8TA, UK.

51 Institute of Medical Epidemiology, Biostatistics, and Informatics, Martin-Luther-University Halle-Wittenberg, 06112 Halle (Saale), Germany.

52 Second Department of Internal Medicine, Salzburger Landeskliniken, 5020 Salzburg, Austria.

53 Cyprus International Institute for Environmental and Public Health in association with the Harvard School of Public.

54 Cyprus Cardiovascular and Educational Research trust, Nicosia, 2368, Cyprus.

55 Grupo de Medicina Xenómica, Centro Nacional de Genotipado (CEGEN-ISCIII), Universidade de Santiago de Compostela. RECAVA.

56 Department of Neurology, Innsbruck Medical University, Anichstraße 35, A-6020 Innsbruck, Austria.

57 Institute for Clinical and Experimental medicine, Videnska 1958/9, Prague 4, 14021, Czech Republic.

58 Division of Genetics, Department of Medicine, Brigham and Women's Hospital, Harvard Medical School, Boston, MA 02115, USA.

59 Harvard Bioinformatics and Integrative Genomics, Boston, Massachusetts, 02115 USA.

60 Partners HealthCare Center for Personalized Genetic Medicine, Boston, Massachusetts, USA.

61 Division of Rheumatology, Immunology, and Allergy, Brigham and Women's Hospital, Boston, Massachusetts, USA.

62 Faculty of Medical and Human Sciences, University of Manchester, Manchester, UK.

63 Laboratory of Epidemiology, Demography and Biometry, National Institute on Aging, Bethesda, MD 20892, USA.

64 Department of Medicine, University of Maryland School of Medicine, Baltimore, Maryland, 21201, USA.

65 Program in Personalized and Genomic Medicine, University of Maryland, Baltimore, Maryland, 21201, USA.

66 Geriatric Research and Education Clinical Center, Veterans Administration Medical Center, Baltimore, Maryland, USA.

67 Division of Epidemiology and Community Health, School of Public Health, University of Minnesota, Minneapolis, Minnesota, United States of America.

68 Department of Biomedical Engineering, Johns Hopkins University, Baltimore, MD 21218, USA.

69 Electrocardiology, ECG Core Lab, University of Glasgow, Glasgow G31 2ER.

70 Clinical Pharmacology and the Cambridge Institute of Medical Research, University of Cambridge, Addenbrooke’s Hospital, Cambridge, UK.

71 Dept of Cardiovascular Science, University of Leicester, Glenfield Hospital, Groby Road, Leicester, LE3 9QP, UK.

72 Department of Cardiology, Lund University, Lund, Sweden.

73 German Cancer Research Centre, Division of Cancer Epidemiology, 69120 Heidelberg, Germany.

74 Department of Pharmacology, Ernst-Moritz-Arndt-University of Greifswald, 17487 Greifswald, Germany.

75 Department of Medicine III, Medical Faculty, Martin-Luther-University Halle-Wittenberg, 06112 Halle (Saale), Germany.

76 Medical Genetics Unit, Casa Sollievo della Sofferenza, San Giovanni Rotondo, Italy.

77 Department of Epidemiology, University of Washington, Seattle, WA, USA.

78 Department of Health Services, University of Washington, Seattle, WA, USA.

79 Medical Genetics Institute, Cedars-Sinai Medical Center, Los Angeles, Calif.

80 Department of Public Health, Faculty of Medicine, University of Split, Soltanska 2, 21000 Split, Croatia.

81 Vascular Screening and Diagnostic Centre, London, WB1 7BZ, UK.

82 Division of Cardiology, Department of Medicine, Landspitali University Hospital, 101 Reykjavik, Iceland.

83 Departments of Biomedical Informatics and Medicine, Vanderbilt University School of Medicine, Nashville, TN, 37232, USA.

84 Departments of Medicine and Pharmacology and the Office of Personalized Medicine, Vanderbilt University, Nashville, TN, 37232, USA.

85 Merck Research Laboratory, Cardiovascular Disease Franchise, Rahway, NJ, 07065, USA.

86 Department of Biostatistics, Boston University School of Public Health, Boston, MA, 02118, USA.

87 Department of Mathematics and Statistics, Boston University, Boston, MA, 02115, USA.

88 Cardiology Division, Massachusetts General Hospital, MA, 02114, USA.

89 Cardiovascular Department, Ospedali Riuniti and University of Trieste, Trieste, Italy, 34149.

90 University of Trieste, Trieste, Italy, 34137.

91 Fundación Pública Galega de Medicina Xenómica (SERGAS), CIBERER, Instituto de Ciencias Forenses (Universidade de Santiago de Compostela).

92 Laboratory of Neurogenetics, National Institute on Aging, National Institutes of Health, Bethesda, MD, 20892, USA.

93 Chronic Disease Epidemiology and Prevention Unit, National Institute for Health and Welfare, PO Box 30, FI-00271 Helsinki, Finland.

94 Department of Medicine, University of Helsinki, FI-00290 Helsinki, Finland.

95 Division of Cardiology, Department of Medicine, Helsinki University Central Hospital, FI-00029 HUS, Helsinki, Finland.

96 Public Health Genomics Unit, National Institute for Health and Welfare, PO Box 30, FI-00271 Helsinki, Finland.

97 Institute for Molecular Medicine Finland FIMM, PO Box 20, FI-00014 University of Helsinki, Helsinki, Finland.

98 Estonian Genome Center, University of Tartu, Tartu, Estonia.

99 Department of Cardiology, University Hospital of Essen, University Duisburg-Essen, Essen, Germany.

100 Department of Genomics, Life & Brain Center, University of Bonn, Bonn, Germany.

101 Institute of Human Genetics, University of Bonn, Bonn, Germany.

102 MRC Epidemiology Unit, Institute of Metabolic Science, Addenbrooke’s Hospital, Cambridge, CB2 0QQ, UK.

103 Mount Sinai School of Medicine, New York, NY, 10029, USA.

104 Department of Biostatistics, Institute of Basic Medical Sciences, University of Oslo, 0317 Oslo, Norway.

105 Institute of Human Genetics, Technische Universität München, Munich 81675, Germany.

106 German Research Center for Environmental Health, Neuherberg, Germany.

107 Institute of Human Genetics, TU München, Klinikum Rechts der Isar, Munich, Germany.

108 Institute for Biological and Medical Imaging, Helmholtz Zentrum München.

109 Institute of Epidemiology II, Helmholtz Zentrum München.

110 Munich Heart Alliance, Munich, Germany.

111 CK-CARE: Christine Kühne - Center for Allergy Research and Education.

112 Department of Dermatology and Allergy, Technische Universität München, Munich, Germany.

113 Research Unit of Molecular Epidemiology, Helmholtz Zentrum München, Germany.

114 Department of Clinical Genetics, Academic Medical Center, Amsterdam, The Netherlands.

115 Clinical Sciences, St George's University of London, London, SW17 0RE, UK.

116 Biomedical Sciences, St Georges's University of London, Cranmer Terrace, London, SW17 0RE, UK.

117 The Labatt Family Heart Centre and Department of Pediatrics, The Hospital for Sick Children, Toronto, Ontario Canada M5G 1X8.

118 The Centre for Applied Genomics, The Hospital for Sick Children, Toronto, Ontario Canada M5G 1X8.

119 Center for Statistical Genetics, Department of Biostatistics, University of Michigan, Ann Arbor, MI, USA.

120 General Practice and Primary Care, University of Glasgow, 1 Horselethill Road, Glasgow G12 9LX, UK.

121 Centre for Population Health Sciences, University of Edinburgh, Teviot Place, Edinburgh, EH8 9AG, Edinburgh, Scotland.

122 Biobank PopGen, Institute of Experimental Medicine, Christian-Albrechts-University of Kiel, 24105 Kiel, Germany.

123 Department of Internal Medicine III, University Medical Center Schleswig-Holstein, Campus Kiel, 24105 Kiel, Germany.

124 Department of Internal Medicine, University of Groningen, University Medical Center Groningen, Groningen, The Netherlands.

125 Netherlands Consortium for Healthy Aging (NCHA), The Netherlands.

126 Department of Medical Informatics, Erasmus Medical Center, P.O. Box 2040, 3000CA, Rotterdam, The Netherlands.

127 Department of Internal Medicine, Erasmus Medical Center, P.O. Box 2040, 3000CA, Rotterdam, The Netherlands.

128 First Department of Internal Medicine, Salzburger Landeskliniken, 5020 Salzburg, Austria.

129 Istituto di Ricerca Genetica e Biomedica, CNR, Monserrato, 09042 Cagliari, Italy.

130 Laboratory of Genetics, Intramural Research Program, National Institute on Aging, National Institutes of Health, Baltimore, MD 21224, USA.

131 Institute for Community Medicine, University Medicine Greifswald, 17487 Greifswald, Germany.

132 Interfaculty Institute for Genetics and Functional Genomics, Ernst-Moritz-Arndt-University Greifswald, 17487 Greifswald, Germany.

133 Department of Twin Research and Genetic Epidemiology, King's College London, London, United Kingdom.

134 Department of Public Health and Caring Sciences, Uppsala Science Park, SE-751 85 Uppsala, Sweden.

135 Department of Medical Sciences, Uppsala University, Akademiska sjukhuset, SE-751 85 Uppsala, Sweden.

136 Dept of Medical Sciences, Molecular Medicine and Science for Life Laboratory, Uppsala University, SE-751 85 Uppsala, Sweden.

137 Department of Clinical Physiology, University of Tampere, Tampere University Hospital, PO Box 2000, 33521 Tampere, Finland.

138 Department of Clinical Physiology and Nuclear Medicine, University of Turku, Turku University Hospital PO Box 52,20521 Turku, Finland.

139 Department of Medicine, University of Turku, Turku University Hospital PO Box 52,20521 Turku, Finland.

140 Xenética de enfermedades cardiovasculares e oftalmolóxicas. Instituto de investigación sanitaria de Santiago, SERGAS.

141 Occupational and Environmental Medicine, Department of Public Health and Community Medicine, Institute of Medicine, Sahlgrenska Academy, University of Gothenburg, 40530 Gothenburg, Sweden.

142 Global Epidemiology, AstraZeneca R&D, 431 83 Mölndal, Sweden.

143 Division of Population Health Sciences & Education, St George’s University of London, London SW17 0RE, UK.

144 Department of Neurology, University of Lübeck, 23538 Lübeck, Germany.

145 Department of Neurology, General Central Hospital, 39100 Bolzano, Italy.

146 Genetics and Genome Biology Program,The Hospital for Sick Children Research Institute, Toronto, Ontario, M5G 1X8 , Canada

147 Department of Epidemiology and Prevention, Division of Public Health Sciences, Wake Forest University, Medical Center Boulevard, Winston-Salem, NC 27157, USA.

148 Department of Medicine, University of Washington, Seattle, USA.

149 Human Genetics Research Centre, St. George’s University of London, London, SW17 0RE, United Kingdom

150 Center for Systems Genomics, Pennsylvania State University, University Park, PA, 16802, USA.

151 Inspectorate of Health Care, P.O. BOX 16119, 2500 BC , The Hague, The Netherlands.

152 Center for Biological Sequence Analysis, Department of Systems Biology, Technical University of Denmark, 2800 Lyngby, Denmark.

153 Pediatric Surgical Research Laboratories, MassGeneral Hospital for Children, Massachusetts General Hospital, 02114 Boston, MA, USA.

154 Cardiovascular Genetics Laboratory, Hatter Institute for Cardiovascular Research in Africa, Department of Medicine, University of Cape Town, South Africa.

155 Department of Medicine, University of Stellenbosch, South Africa.

156 Chair of Sudden Death, Department of Family and Community Medicine, College of Medicine, King Saud University, Riyadh, Saudi Arabia.

157 Institute for Bioinformatics and Systems Biology, Helmholtz Zentrum München Germany.

*These authors contributed equally
