# Supplementary figures and images for: Discovery of Genetic Variation on Chromosome 5q22 Associated with Mortality in Heart Failure
Source: PLoS Genet. 2016 May 5;12(5):e1006034. doi: 10.1371/journal.pgen.1006034 (PMC4858216; doi:10.1371/journal.pgen.1006034)

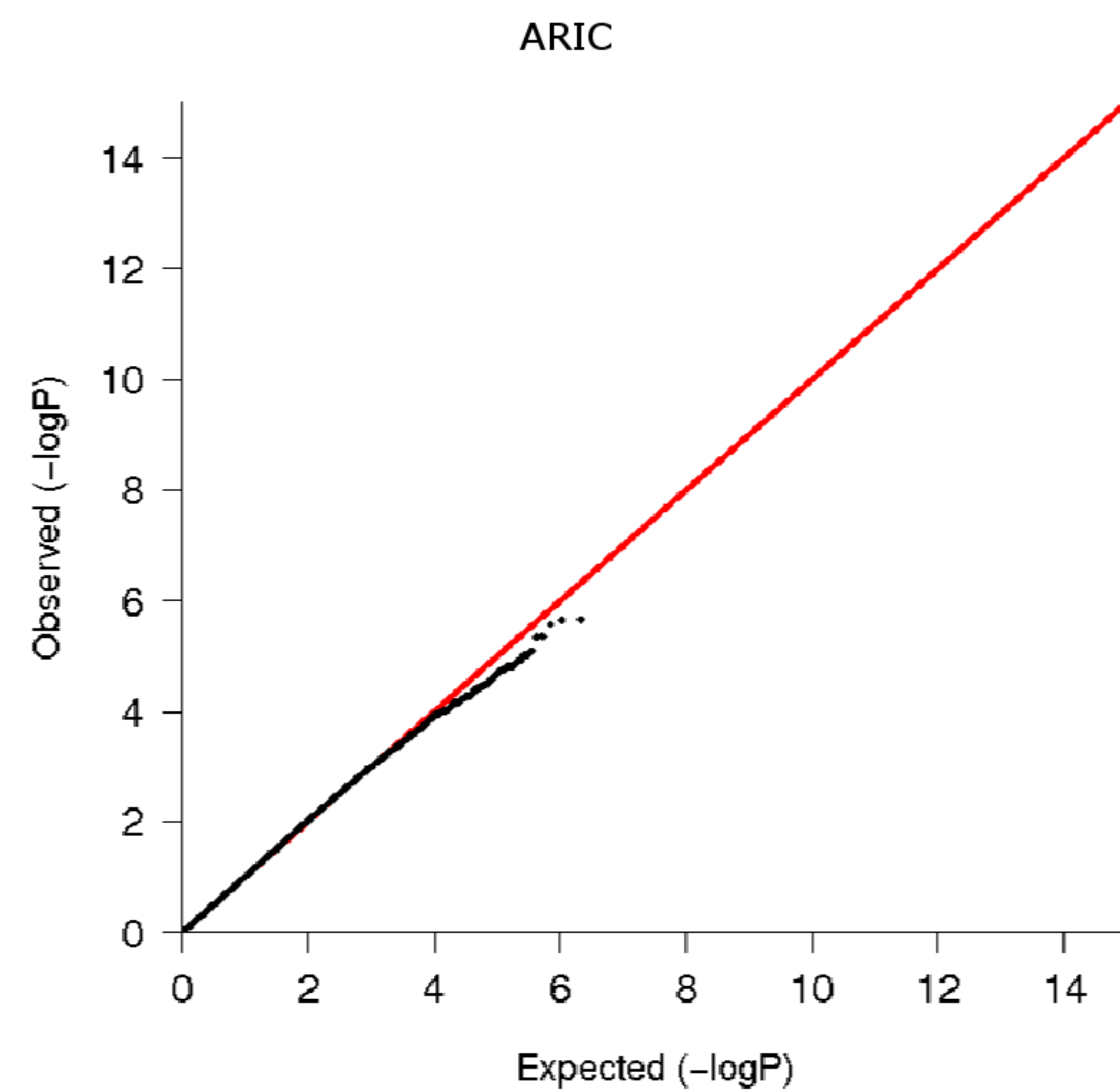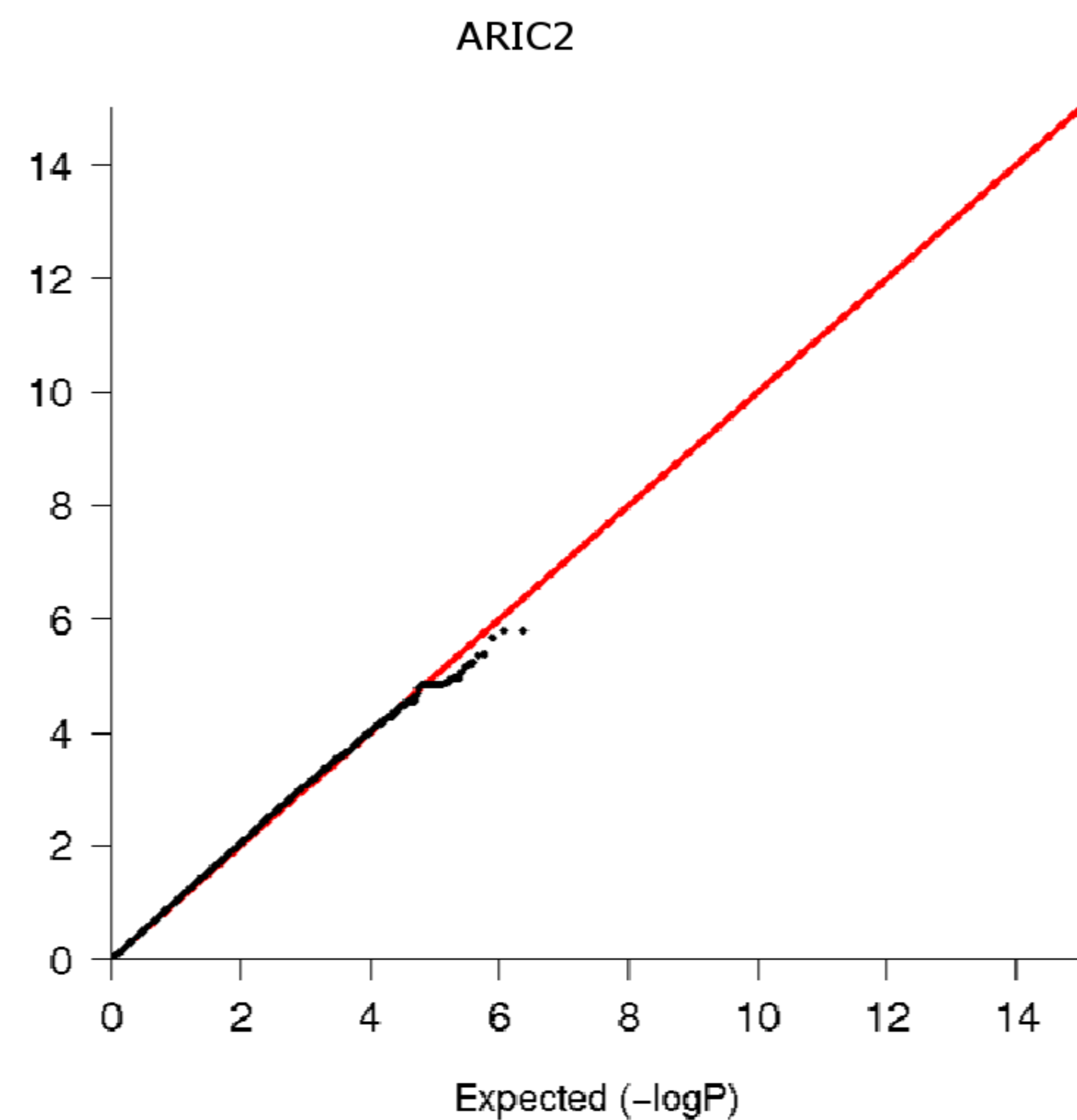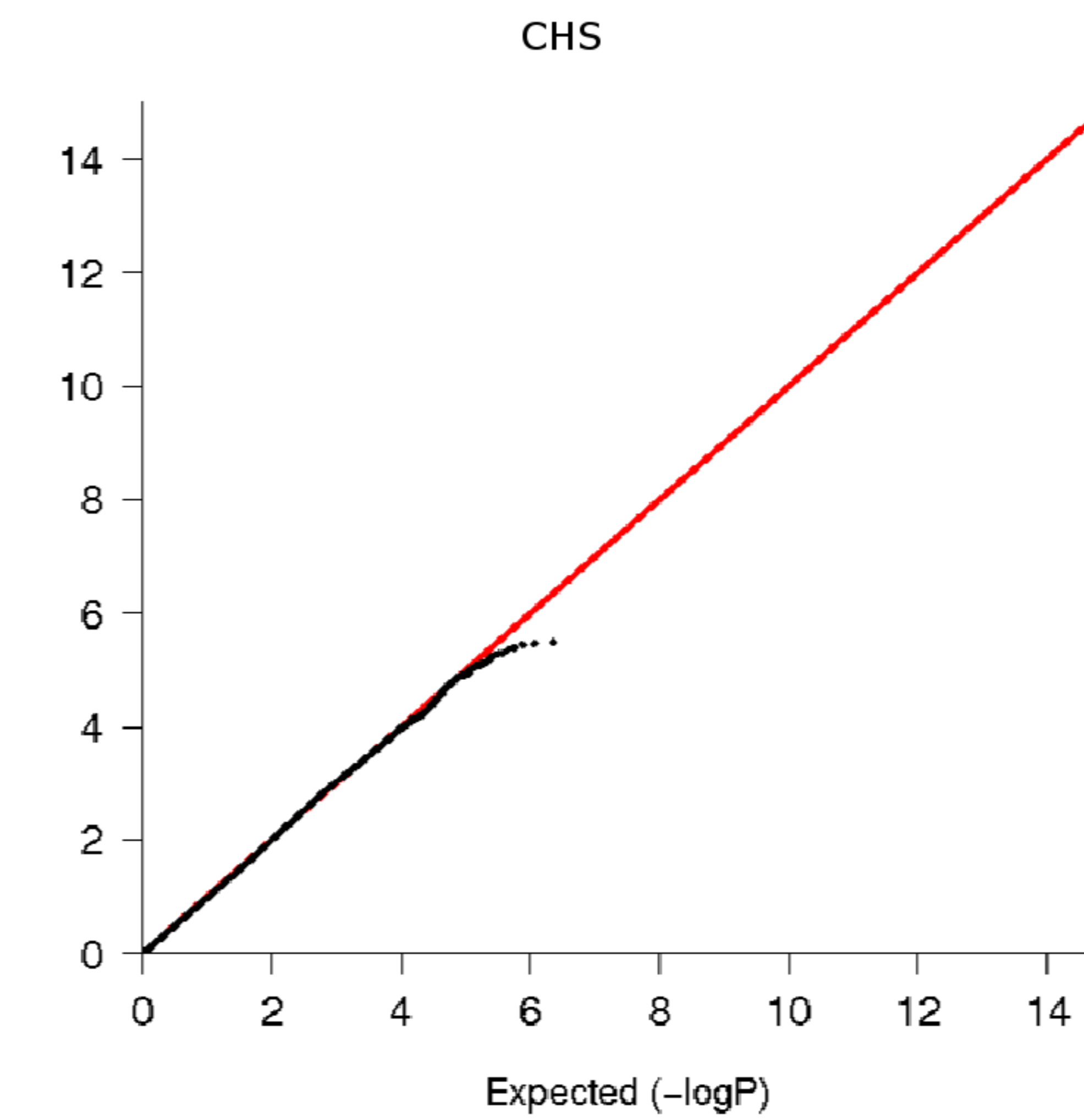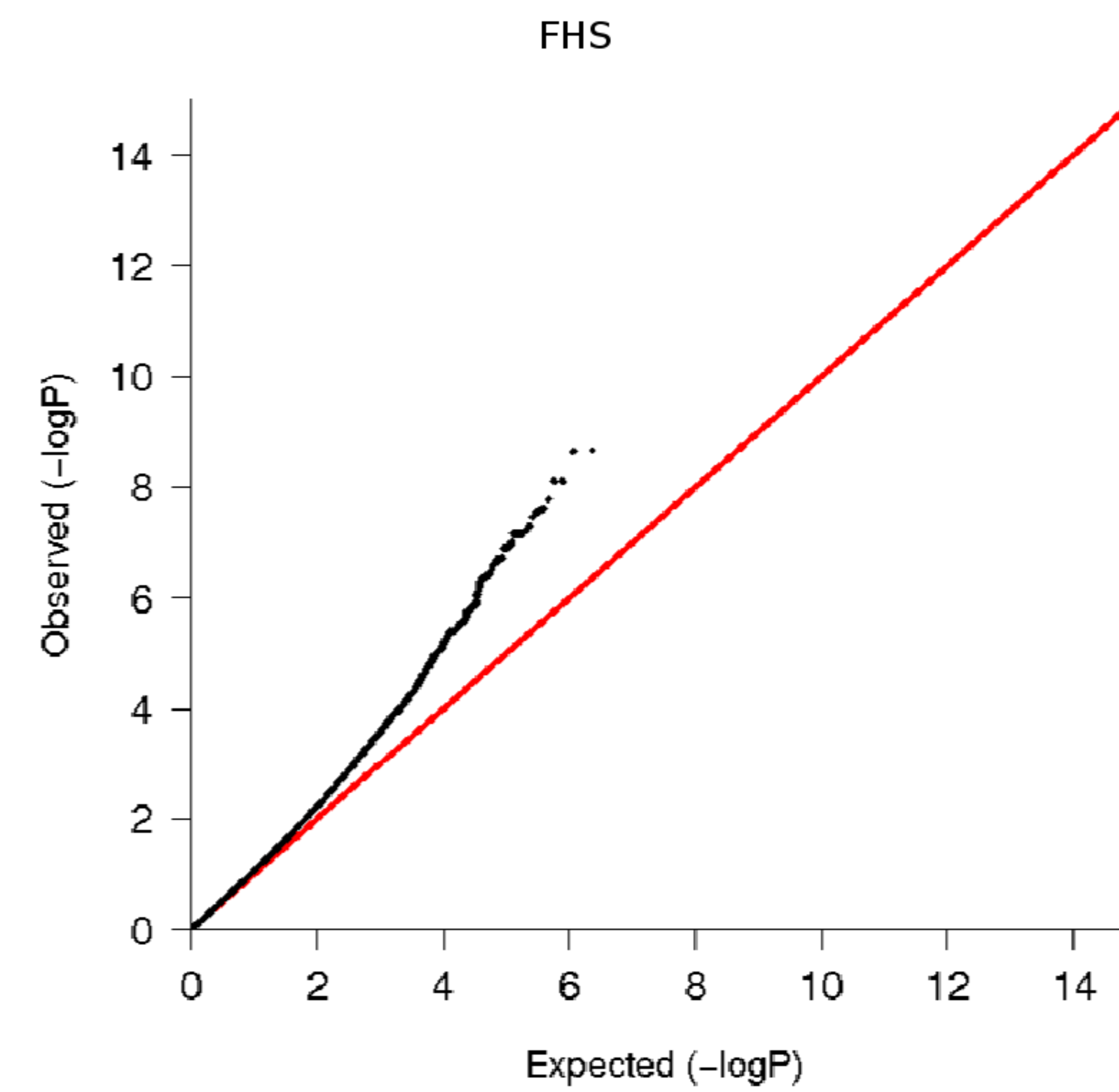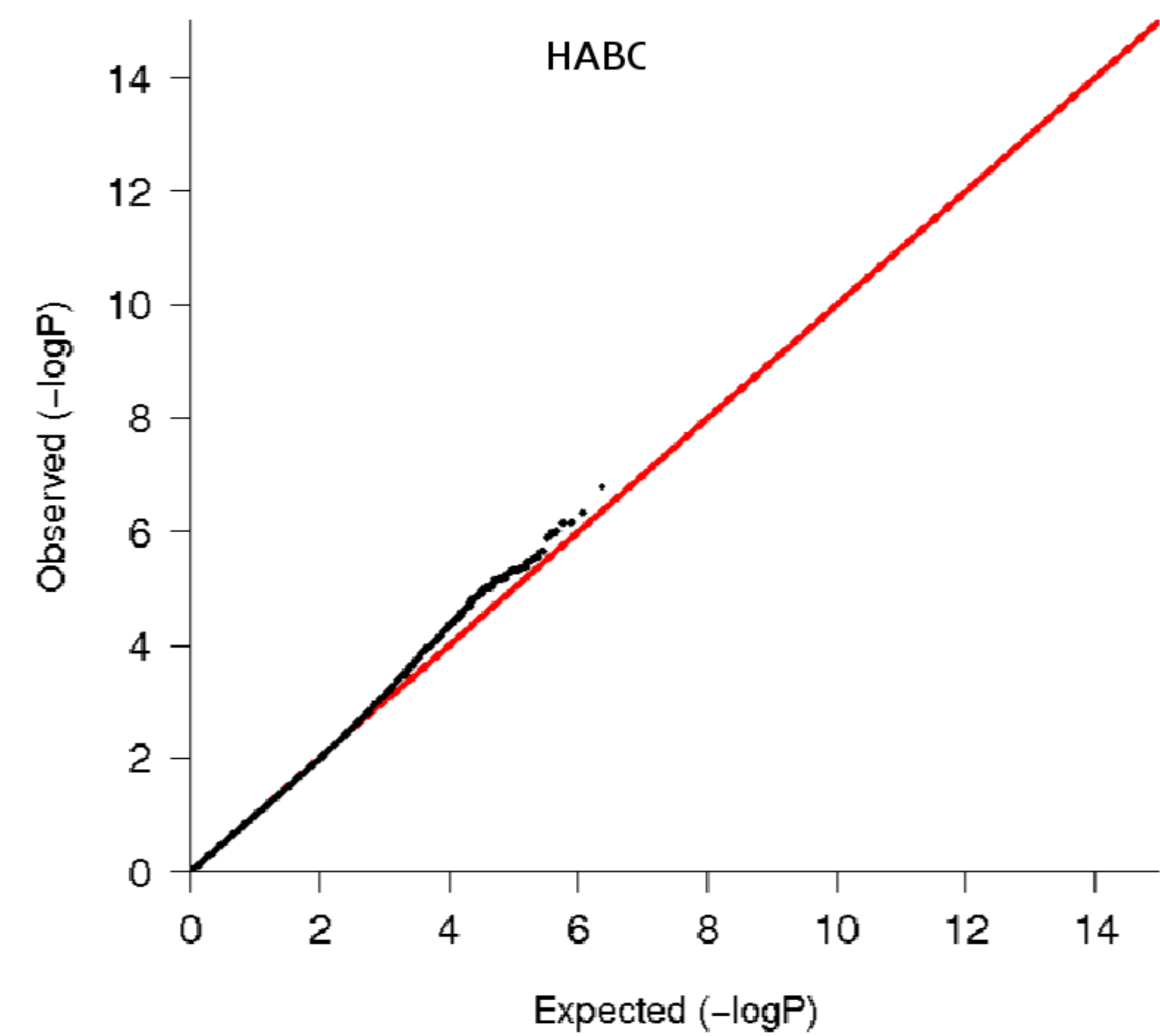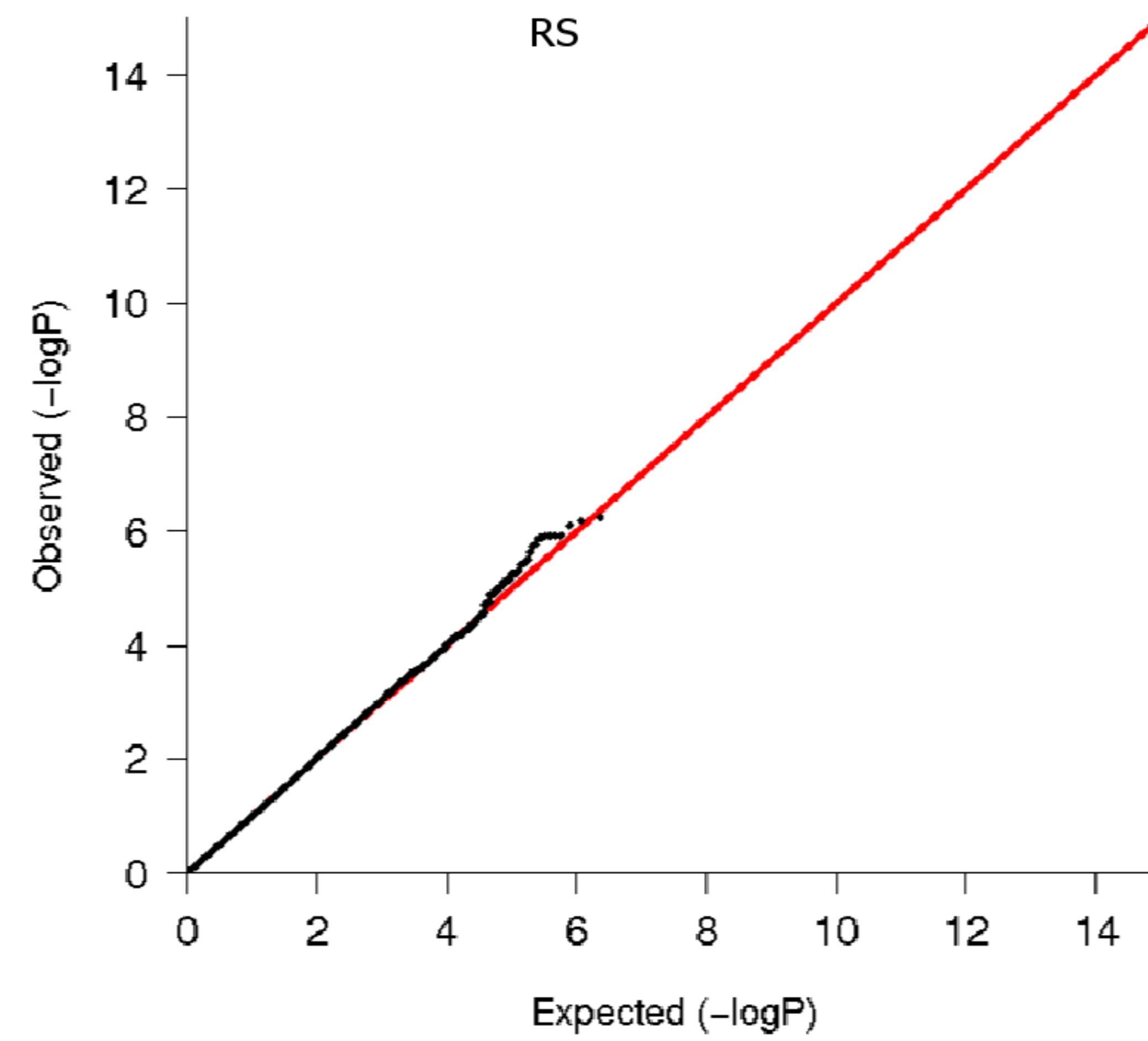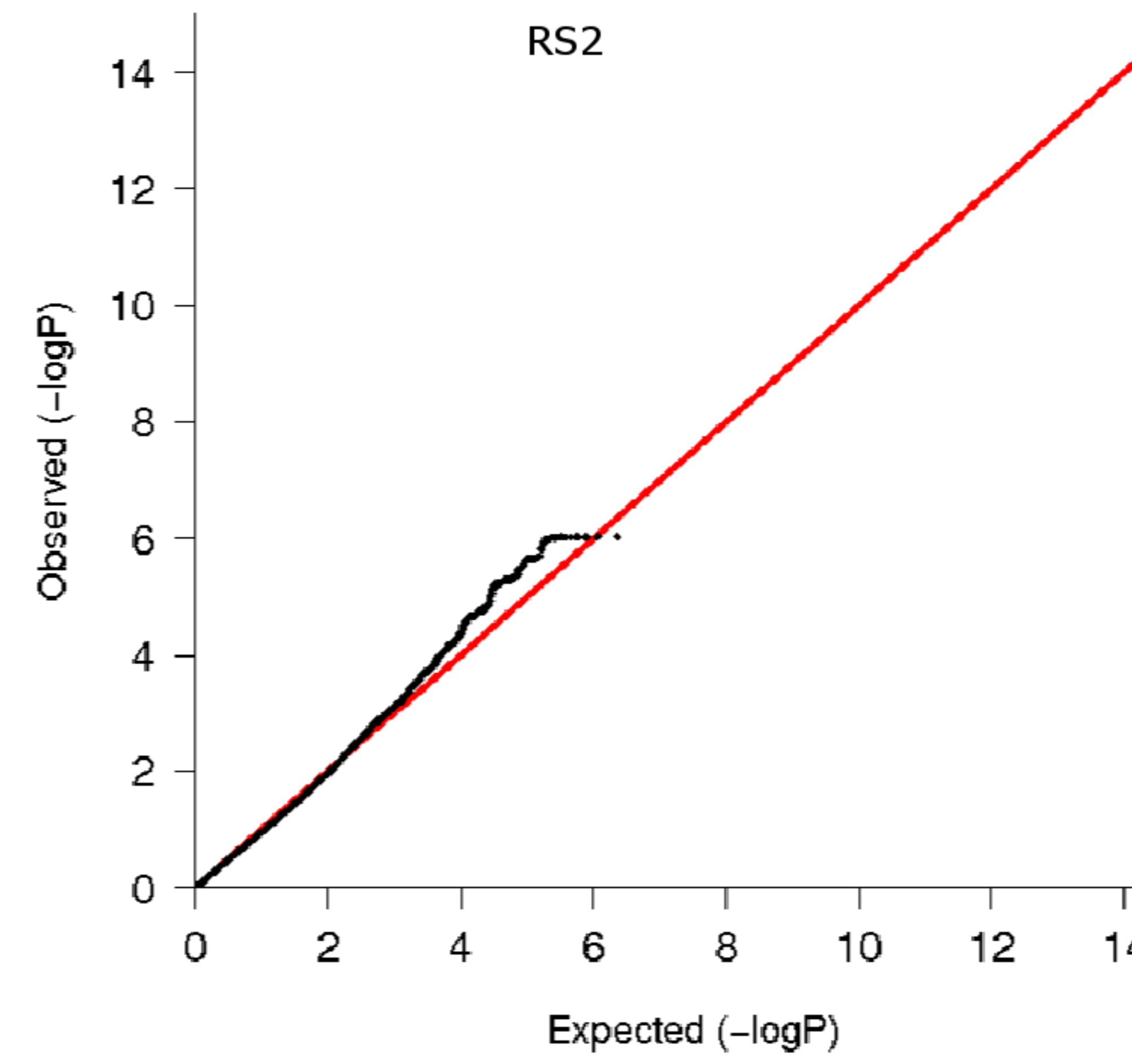

Supplement: S1 Fig — Plotted on the x-axis are expected P-values under the null hypothesis and on the y-axis the observed P-values before genomic control has been applied. (PDF) [file pgen.1006034.s002.pdf]

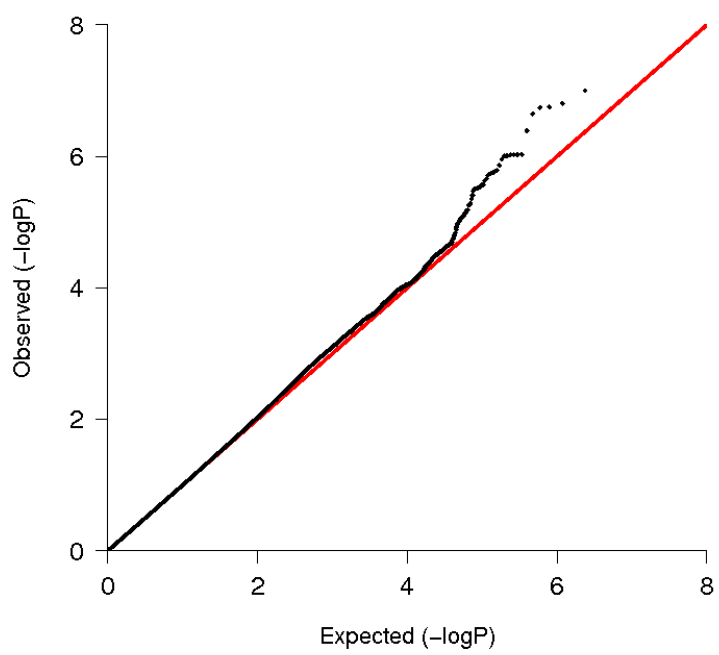

Supplement: S2 Fig — Plotted on the x-axis are expected P-values under the null hypothesis and on the y-axis the observed P-values. (PDF) [file pgen.1006034.s003.pdf]

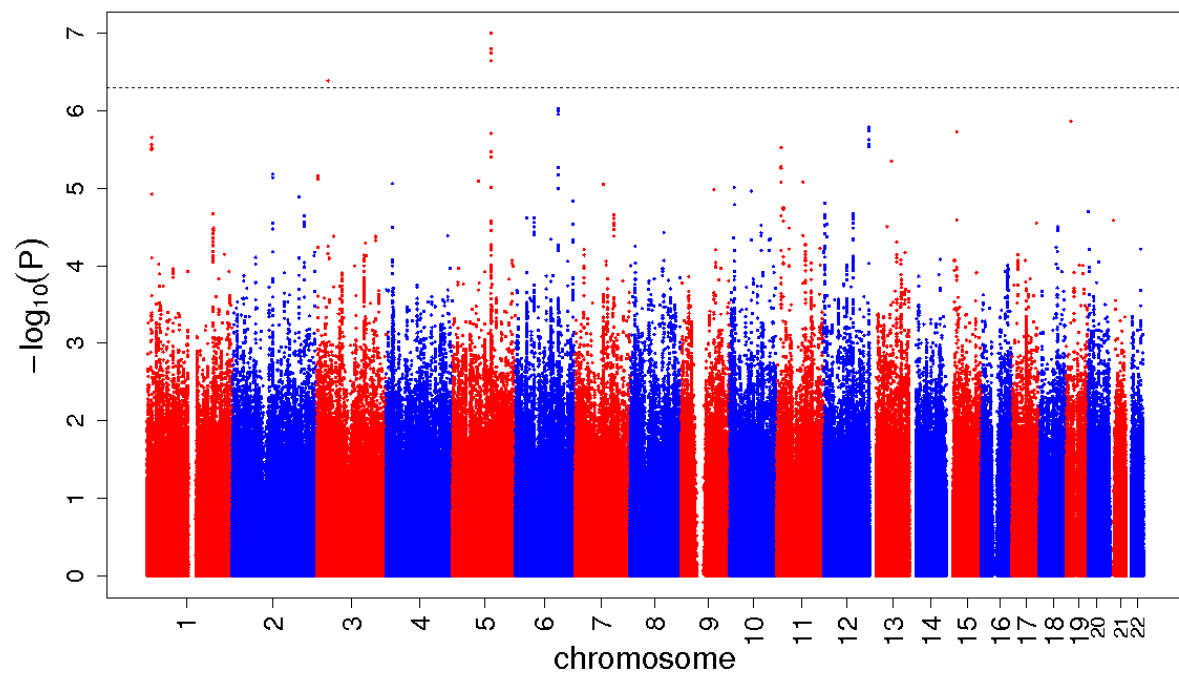

Supplement: S3 Fig — Each dot represents one SNP. The x-axis shows physical position by chromosome and the y-axis–log10 (P-value). The vertical dotted line indicates the a priori specified significance threshold for SNPs to be carried forward to stage 2 (P < 5.0x10^-7^). (PDF) [file pgen.1006034.s004.pdf]

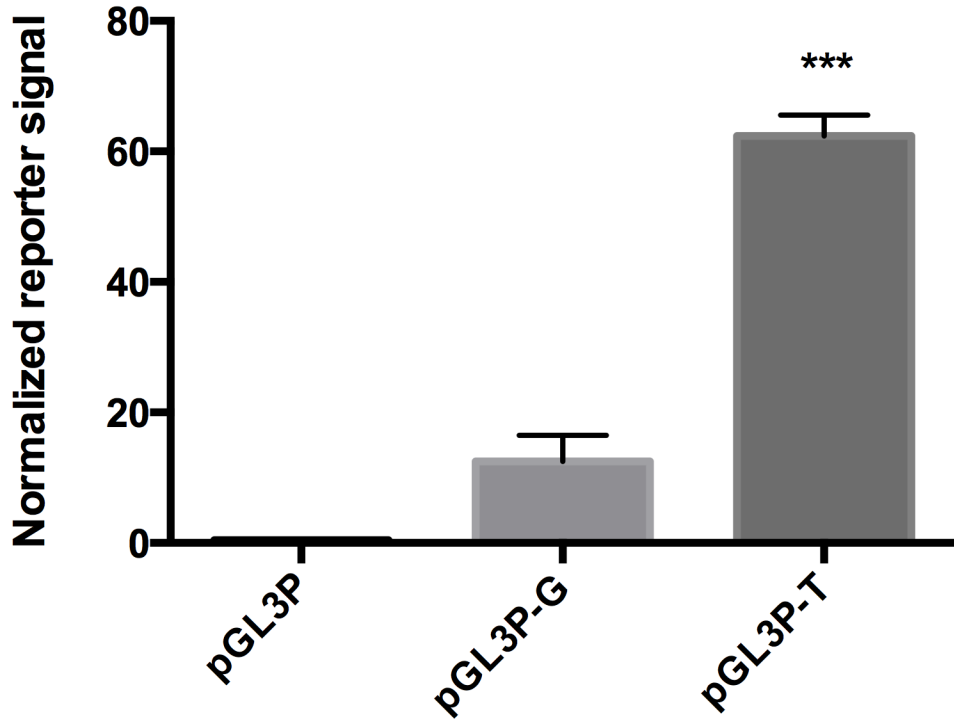

Supplement: S4 Fig — HEK293 cells were transfected with either empty vector (pGL3P) or vectors containing the major (pGL3P-G) or minor (pGL3P-T) allele of rs9885413 together with 100 bp flanking the SNP. Luciferase activity from the vectors was measured after 24 hours and normalized to that of a pRL-null vector. N = 3. ***P < 0.001. (PDF) [file pgen.1006034.s005.pdf]

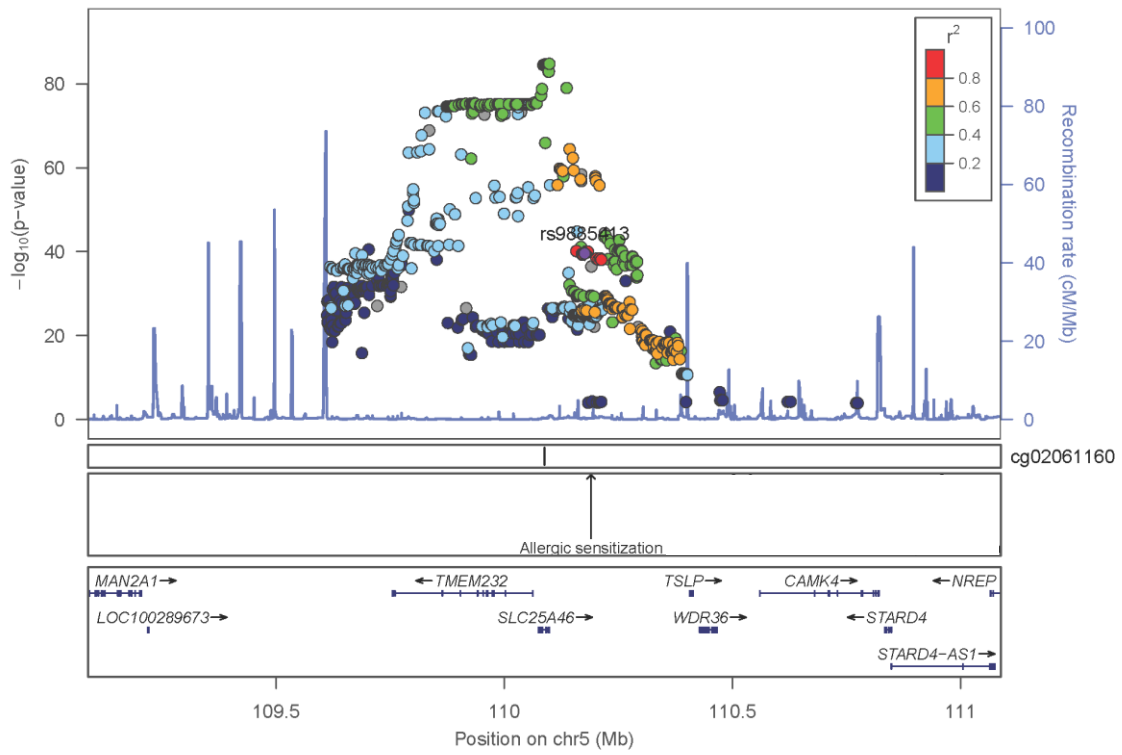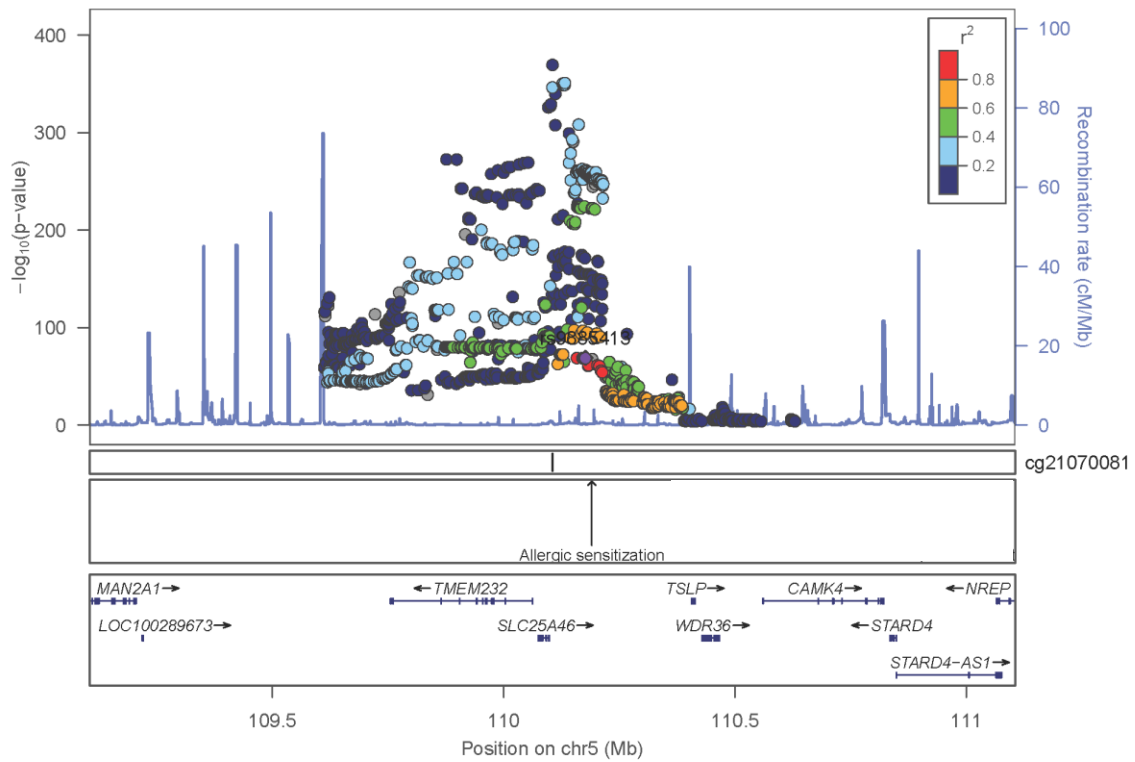

Supplement: S5 Fig — The plot covers the genomic region of +/-1 million bases from each CpG methylation. P-values refer to association of each SNP with two CpG methylation sites on chromosome 5q22: cg02061160 (top panel) and cg21070081 (bottom panel). Circles represent SNPs. The purple circle represents the SNP associated with heart failure mortality (rs9885413). Circle color represents strength of pairwise correlation with rs9885413, with r2 according to the inset. CpG sites are illustrated below the regional plot, as is the location of the SNP associated with allergic sensitization (rs10056340). Recombination rate is plotted in the background and known genes are represented in the bottom of the plot. Positions refer to NCBI build 36. SNP correlations and recombination rates were obtained from the 1000 Genomes pilot and HapMap release 22, respectively. The plot was created using LocusZoom (http://locuszoom.sph.umich.edu/locuszoom/). (PDF) [file pgen.1006034.s006.pdf]

A

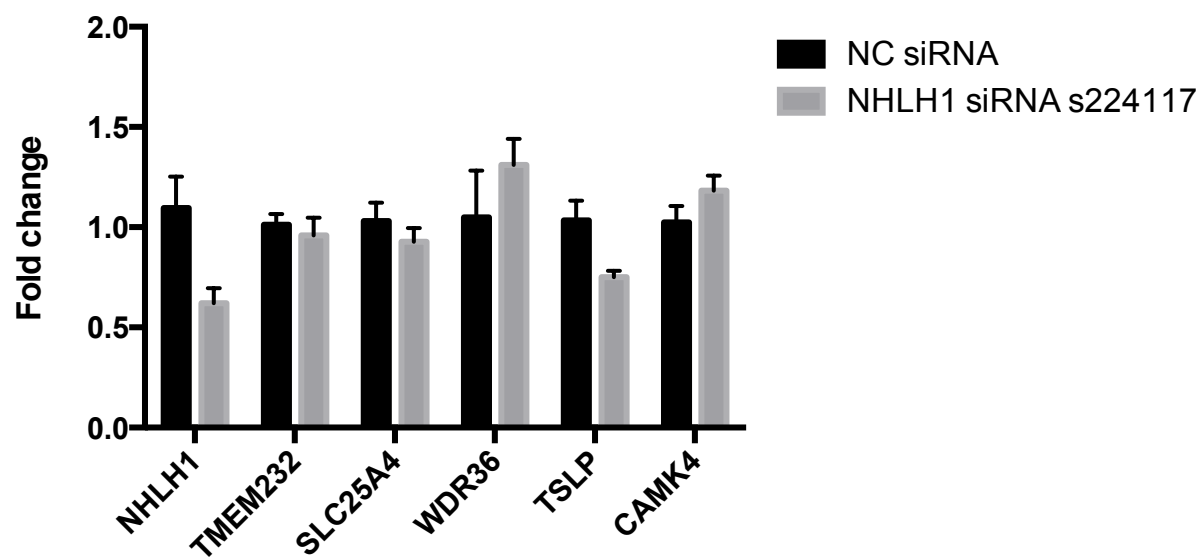

B

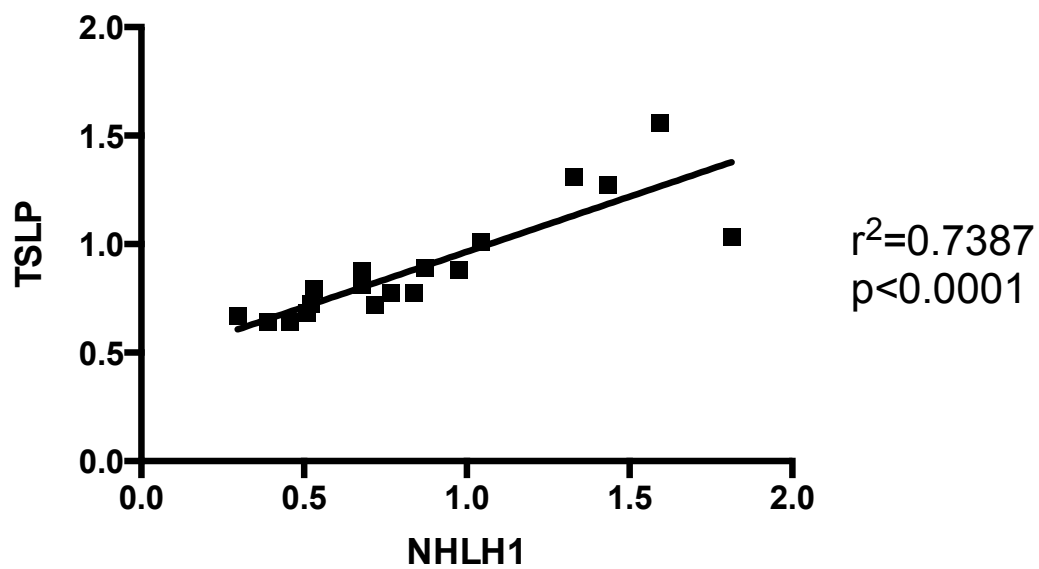

Supplement: S6 Fig — HEK293 cells were transfected with siRNA targeted against NHLH1 and expression of the five genes surrounding rs9885413 were analyzed by qPCR. Expression levels were normalized to the housekeeping gene GAPDH and expressed relative to cells transfected with negative control (NC) siRNA. Results are from three separate experiments with triplicates in each sample group. *p<0.05 in two sample t-tests. A) Fold change for NHLH1 and individual genes at the locus. B) Dose-response relation of NHLH1 knockdown and TSLP expression. (PDF) [file pgen.1006034.s007.pdf]

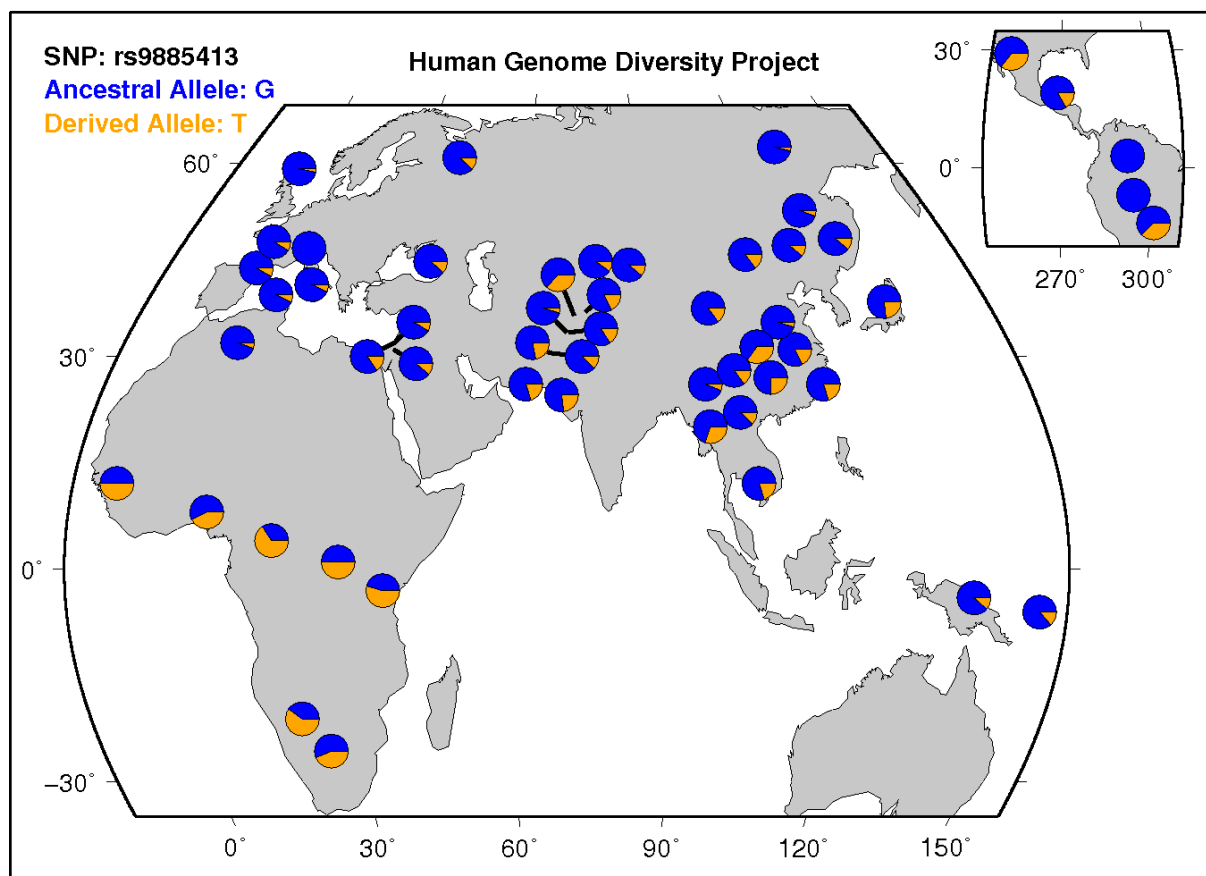

Supplement: S7 Fig — Relative allele frequencies of the ancestral (G, blue) and derived (T, yellow) alleles of rs9885413 presented as pie slices across populations in the Human Genome Diversity Project (HGDP). (PDF) [file pgen.1006034.s008.pdf]
