# Supplementary material for: Discovery of Genetic Variation on Chromosome 5q22 Associated with Mortality in Heart Failure
Source: PLoS Genet. 2016 May 5;12(5):e1006034. doi: 10.1371/journal.pgen.1006034 (PMC4858216; doi:10.1371/journal.pgen.1006034)
Supplement: S1 Table — *Included non-hospitalized deaths but not morbid events. **European Society of Cardiology. ***In a validation sample as previously described [26]. †Signs and symptoms included in Framingham criteria. ‡Cardiac structure and function including cardiomegaly, dilated ventricle, decreased systolic function, or segmental wall-motion abnormalities. §Pharmaceutical treatments include the prescribing of diuretics, digoxin, or vasodilator. (DOCX) [file pgen.1006034.s009.docx]

**S1 Table. Methods of heart failure ascertainment and diagnosis across cohorts**

|  | **Stage 1** | | | | | **Stage 2** | | |
| --- | --- | --- | --- | --- | --- | --- | --- | --- |
|  | *ARIC/ARIC2* | *CHS* | *FHS* | *RS/RS2* | *Health ABC* | *MDCS/MPP* | *PHS* | *PROSPER* |
| Source of information |  |  |  |  |  |  |  |  |
| Self-report, care-giver report | No | Yes | Yes | No | Yes | No | Yes | Yes |
| Administrative records | Yes | Yes | Yes | Yes | Yes | Yes | No | Yes |
| Inclusion of non-hospitalized events | Yes* | Yes | Yes | Yes | No | No | Yes | Yes |
| Review criteria applied | None | CHS | Framingham | ESC** | Health ABC | None | Framingham*** | ESC |
| Event characteristics considered |  |  |  |  |  |  |  |  |
| ICD 9/10 code | Yes | No | No | Yes | No | Yes | No | Yes |
| Signs and symptoms^†^ | No | Yes | Yes | Yes | Yes | No | Yes*** | Yes |
| Cardiac structure and function^‡^ | No | Yes | Yes | Yes | Yes | No | Yes*** | Yes |
| Physician diagnosis | No | Yes | Yes | Yes | Yes | Yes | Yes | Yes |
| Pharmaceutical treatment^§^ | No | Yes | Yes | Yes | Yes | No | Yes*** | Yes |

*Included non-hospitalized deaths but not morbid events.

**European Society of Cardiology.

***In a validation sample as previously described [26].

^†^Signs and symptoms included in Framingham criteria.

^‡^Cardiac structure and function including cardiomegaly, dilated ventricle, decreased systolic function, or segmental wall-motion abnormalities.

^§^Pharmaceutical treatments include the prescribing of diuretics, digoxin, or vasodilator.
