# Supplementary material for: Discovery of Genetic Variation on Chromosome 5q22 Associated with Mortality in Heart Failure
Source: PLoS Genet. 2016 May 5;12(5):e1006034. doi: 10.1371/journal.pgen.1006034 (PMC4858216; doi:10.1371/journal.pgen.1006034)
Supplement: S2 Table — Age, body mass index and follow-up time are presented as mean (standard deviation). Categorical variables are presented as percentages. Body mass index, diabetes, hypertension and smoking refer to the nearest study exam prior to heart failure diagnosis whereas age, follow-up time, mortality rate and all-cause death refer to the time of HF diagnosis. Mortality rate refers to the 1-year Kaplan-Meier estimate, with censoring at loss to follow-up. MI: Myocardial infarction. (DOCX) [file pgen.1006034.s010.docx]

**S2 Table. Characteristics of stage 2 cohorts**

|  | **MDCS** | **MPP** | **PHS** | **PROSPER** |
| --- | --- | --- | --- | --- |
| **HF sample size** | 856 | 203 | 600 | 211 |
| **Age at HF** (years) | 70.9 (7.9) | 69.9 (8.7) | 78.8 (8.0) | 78.1 (3.7) |
| **Male sex** (%) | 59.8 | 81.3 | 100 | 58.3 |
| **Body mass index** (kg/m^2^) | 28.0 (4.8) | 28.6 (5.3) | 26.2 (3.9) | 27.4 (4.5) |
| **Diabetes** (%) | 12.1 | 25.4 | 19.5 | 14.2 |
| **Hypertension** (%) | 86.8 | 94.1 | 70.5 | 57.8 |
| **Current smoking (%)** | 30.7 | 25.6 | 3.9 | 26.1 |
| **History of MI** (%) | 29.4 | 21.2 | 17.7 | 28.0 |
| **Follow-up time** (years) | 4.6 (4.4) | 6.0 (4.9) | 4.1 (3.1) | 1.8 (0.9) |
| **Mortality rate, 1 year** | 0.19 | 0.09 | 0.12 | 0.23 |
| **All-cause death** (n, %) | 456 (53.0) | 69 (34.0) | 275 (45.8) | 89 (42.2) |

Age, body mass index and follow-up time are presented as mean (standard deviation). Categorical variables are presented as percentages. Body mass index, diabetes, hypertension and smoking refer to the nearest study exam prior to heart failure diagnosis whereas age, follow-up time, mortality rate and all-cause death refer to the time of HF diagnosis. Mortality rate refers to the 1-year Kaplan-Meier estimate, with censoring at loss to follow-up. MI: Myocardial infarction.
