# Supplementary material for: Discovery of Genetic Variation on Chromosome 5q22 Associated with Mortality in Heart Failure
Source: PLoS Genet. 2016 May 5;12(5):e1006034. doi: 10.1371/journal.pgen.1006034 (PMC4858216; doi:10.1371/journal.pgen.1006034)
Supplement: S3 Table — Genome position (POS) refers to NCBI build 36. CHR, chromosome. STR, strand. CA, coded allele. A2, non-coded allele. N, sample size. BETA, beta estimate. SE, standard error of beta estimate. P, P-value. (DOCX) [file pgen.1006034.s011.docx]

**S3 Table. Results for all SNPs with p<10^-5^.**

| **SNP** | **CHR** | **POS** | **STR** | **CA** | **A2** | **N** | **BETA** | **SE** | **P** |
| --- | --- | --- | --- | --- | --- | --- | --- | --- | --- |
| rs9885413 | 5 | 110204027 | + | G | T | 2828 | -0.333 | 0.062 | 9.97x10^-8^ |
| rs12658193 | 5 | 110200417 | + | G | T | 2828 | -0.333 | 0.063 | 1.57x10^-7^ |
| rs11956079 | 5 | 110198295 | + | C | T | 2828 | -0.332 | 0.064 | 1.77x10^-7^ |
| rs10069077 | 5 | 110197925 | + | C | T | 2828 | -0.332 | 0.064 | 1.81x10^-7^ |
| rs10068260 | 5 | 110209995 | + | T | A | 2828 | 0.350 | 0.068 | 2.25x10^-7^ |
| rs12638540 | 3 | 32463538 | + | G | A | 2828 | 0.405 | 0.080 | 4.09x10^-7^ |
| rs2315841 | 6 | 122319575 | + | C | A | 2828 | 0.268 | 0.055 | 9.35x10^-7^ |
| rs9490362 | 6 | 122315940 | + | C | G | 2828 | 0.268 | 0.055 | 9.41x10^-7^ |
| rs9490363 | 6 | 122315962 | + | C | T | 2828 | -0.268 | 0.055 | 9.42x10^-7^ |
| rs9375078 | 6 | 122307104 | + | C | A | 2828 | 0.268 | 0.055 | 9.50x10^-7^ |
| rs1402532 | 6 | 122304954 | + | G | A | 2828 | -0.268 | 0.055 | 9.79x10^-7^ |
| rs1607934 | 6 | 122302456 | + | G | T | 2828 | -0.268 | 0.055 | 9.94x10^-7^ |
| rs9375081 | 6 | 122329048 | + | G | A | 2828 | -0.264 | 0.054 | 1.11x10^-6^ |
| rs4528684 | 19 | 14212574 | + | C | T | 2783 | -0.331 | 0.068 | 1.36x10^-6^ |
| rs7965445 | 12 | 130428856 | + | G | A | 2828 | -0.255 | 0.053 | 1.62x10^-6^ |
| rs12422521 | 12 | 130426315 | + | C | T | 2828 | 0.254 | 0.053 | 1,70x10^-6^ |
| rs7299374 | 12 | 130428360 | + | G | T | 2828 | -0.254 | 0.053 | 1,77x10^-6^ |
| rs12422973 | 12 | 130430537 | + | C | G | 2828 | 0.253 | 0.053 | 1,81x10^-6^ |
| rs2125623 | 15 | 29616999 | + | C | T | 2828 | 0.200 | 0.042 | 1,85x10^-6^ |
| rs9326808 | 5 | 110150873 | + | C | T | 2828 | 0.393 | 0.083 | 1,95x10^-6^ |
| rs10927603 | 1 | 15168271 | + | C | T | 2828 | 0.186 | 0.039 | 2,21x10^-6^ |
| rs7977251 | 12 | 130436978 | + | C | T | 2828 | -0.255 | 0.054 | 2,35x10^-6^ |
| rs7976504 | 12 | 130437027 | + | G | A | 2828 | -0.255 | 0.054 | 2,69x10^-6^ |
| rs10754875 | 1 | 15166618 | + | G | A | 2828 | 0.185 | 0.039 | 2,70x10^-6^ |
| rs6486658 | 12 | 130432243 | + | C | T | 2828 | -0.247 | 0.053 | 2,91x10^-6^ |
| rs7120489 | 11 | 12425973 | + | G | A | 2828 | -0.301 | 0.064 | 2,95x10^-6^ |
| rs2175773 | 1 | 15172259 | + | G | A | 2828 | -0.182 | 0.039 | 3,00x10^-6^ |
| rs4575078 | 1 | 15168869 | + | G | T | 2828 | -0.184 | 0.039 | 3,07x10^-6^ |
| rs7554572 | 1 | 15173226 | + | C | T | 2828 | -0.184 | 0.039 | 3,10x10^-6^ |
| rs880923 | 1 | 15168493 | + | C | G | 2828 | -0.184 | 0.039 | 3,13x10^-6^ |
| rs7724652 | 5 | 110149062 | + | C | A | 2828 | -0.340 | 0.073 | 3,35x10^-6^ |
| rs17447390 | 5 | 110144549 | + | G | A | 2828 | 0.340 | 0.074 | 3,91x10^-6^ |
| rs656316 | 13 | 62874290 | + | G | A | 1990 | -0.551 | 0.120 | 4,45x10^-6^ |
| rs933934 | 11 | 12411657 | + | G | T | 2828 | 0.281 | 0.062 | 5,25x10^-6^ |
| rs6569238 | 6 | 122330405 | + | C | T | 2828 | 0.252 | 0.055 | 5,37x10^-6^ |
| rs11022357 | 11 | 12418898 | + | C | T | 2828 | -0.281 | 0.062 | 5,47x10^-6^ |
| rs10496499 | 2 | 116090407 | + | C | A | 2828 | 0.220 | 0.049 | 6,56x10^-6^ |
| rs9372670 | 6 | 122331481 | + | C | T | 2828 | -0.255 | 0.057 | 6,71x10^-6^ |
| rs13075438 | 3 | 3316646 | + | C | A | 2828 | 0.236 | 0.053 | 6,94x10^-6^ |
| rs2901337 | 2 | 116070814 | + | G | A | 2828 | 0.219 | 0.049 | 7,27x10^-6^ |
| rs6773870 | 3 | 3314720 | + | G | T | 2828 | -0.235 | 0.052 | 7,66x10^-6^ |
| rs17670147 | 5 | 73940490 | + | G | A | 2828 | -0.321 | 0.072 | 8,03x10^-6^ |
| rs7129111 | 11 | 75543144 | + | C | T | 2828 | -0.271 | 0.061 | 8,26x10^-6^ |
| rs10741590 | 11 | 12419894 | + | C | T | 2828 | -0.270 | 0.061 | 8,31x10^-6^ |
| rs7683977 | 4 | 19096126 | + | C | T | 2828 | -0.194 | 0.044 | 8,70x10^-6^ |
| rs2190523 | 7 | 81748107 | + | C | T | 1990 | 0.639 | 0.144 | 8,82x10^-6^ |
| rs10044330 | 5 | 109869920 | + | G | A | 2828 | -0.379 | 0.086 | 9,67x10^-6^ |
| rs10795987 | 10 | 12934587 | + | G | A | 2828 | -0.152 | 0.034 | 9,71x10^-6^ |

Genome position (POS) refers to NCBI build 36. CHR, chromosome. STR, strand. CA, coded allele. A2, non-coded allele. N, sample size. BETA, beta estimate. SE, standard error of beta estimate. P, p-value.
