# Supplementary material for: Discovery of Genetic Variation on Chromosome 5q22 Associated with Mortality in Heart Failure
Source: PLoS Genet. 2016 May 5;12(5):e1006034. doi: 10.1371/journal.pgen.1006034 (PMC4858216; doi:10.1371/journal.pgen.1006034)
Supplement: S4 Table — CHR, chromosome. CA, coded allele. A2, non-coded allele. Caf, coded allele frequency. N, sample size. Beta, beta estimate. Se, standard error of beta estimate. P, p-value. (DOCX) [file pgen.1006034.s012.docx]

**S4 Table. Cohort-specific results for SNPs in combined stages 1 and 2.**

|  | **rs9885413** | | | | | | | | **rs12638540** | | | | | | | |
| --- | --- | --- | --- | --- | --- | --- | --- | --- | --- | --- | --- | --- | --- | --- | --- | --- |
| **Cohort** | **Strand** | **CA** | **A2** | **Caf** | **N** | **Beta** | **Se** | **P** | **Strand** | **Ca** | **A2** | **Caf** | **N** | **Beta** | **SE** | **P** |
| ARIC1 | + | T | G | 0.07 | 691 | 0.24 | 0.14 | 0.091 | + | G | A | 0.05 | 691 | 0.42 | 0.16 | 0.01 |
| ARIC2 | + | T | G | 0.07 | 84 | 0.41 | 0.42 | 0.35 | + | G | A | 0.04 | 84 | 0.53 | 0.75 | 0.51 |
| CHS | + | T | G | 0.07 | 838 | 0.40 | 0.11 | 1.7x10^-4^ | + | G | A | 0.04 | 838 | 0.34 | 0.14 | 0.01 |
| FHS | + | T | G | 0.05 | 249 | 0.36 | 0.19 | 0.05 | + | G | A | 0.03 | 249 | -0.05 | 0.52 | 0.93 |
| HABC | + | T | G | 0.07 | 173 | 0.78 | 0.23 | 8x10^-4^ | + | G | A | 0.04 | 173 | 0.14 | 0.39 | 0.72 |
| Malmo | + | T | G | 0.06 | 1013 | 0.36 | 0.12 | 0.004 | + | G | A | 0.05 | 1013 | 0.12 | 0.15 | 0.41 |
| PHS | + | T | G | 0.08 | 591 | 0.03 | 0.14 | 0.83 | + | G | A | 0.04 | 581 | 0.11 | 0.17 | 0.52 |
| PROSPER | + | T | G | 0.07 | 211 | 0.29 | 0.28 | 0.31 | + | G | A | 0.03 | 211 | -0.57 | 0.72 | 0.42 |
| RS1 | + | T | G | 0.06 | 748 | 0.15 | 0.13 | 0.24 | + | G | A | 0.04 | 748 | 0.54 | 0.14 | 3x10^-4^ |
| RS2 | + | T | G | 0.07 | 45 | 0.51 | 0.55 | 0.38 | + | G | A | 0.06 | 45 | -0.23 | 0.80 | 0.77 |
| **Combined** | + | T | G | 0.07 | 3895 | 0.31 | 0.05 | 2.7x10^-9^ | + | G | A | 0.04 | 3885 | 0.30 | 0.07 | 3x10^-6^ |

CHR, chromosome. CA, coded allele. A2, non-coded allele. Caf, coded allele frequency. N, sample size. Beta, effect estimate. Se, standard error of beta estimate. P, p-value.
