# Supplementary material for: Discovery of Genetic Variation on Chromosome 5q22 Associated with Mortality in Heart Failure
Source: PLoS Genet. 2016 May 5;12(5):e1006034. doi: 10.1371/journal.pgen.1006034 (PMC4858216; doi:10.1371/journal.pgen.1006034)
Supplement: S5 Table — (DOCX) [file pgen.1006034.s013.docx]

**S5 Table. Allele frequency distribution across major causes of mortality.**

| **Cause of death** | **Proportion of deaths** | **Frequency of minor (risk) homozygotes or heterozygotes** |
| --- | --- | --- |
| Neoplasms | 0.19 | 0.13 |
| Cardiovascular  Ischemic heart disease  Cerebrovascular | 0.51  0.36  0.07 | 0.14  0.15  0.14 |
| Infectious | 0.01 | 0.00 |
| Injuries | 0.01 | 0.09 |
| Psychiatric | 0.02 | 0.15 |
| Respiratory | 0.10 | 0.15 |
| Renal | 0.02 | 0.16 |
| Neurological | 0.03 | 0.13 |
| Endocrine | 0.03 | 0.19 |
| Blood diseases | 0.001 | 0.00 |
| Digestive | 0.02 | 0.15 |
| Other | 0.05 | 0.12 |
| Total | 1.0 | 0.14 |

Based on International Classification of Disease codes from death certificates for 2420 deaths in heart failure cases from five cohorts: Atherosclerosis Risk in Communities (ARIC), Cardiovascular Health Study (CHS), Malmö Diet and Cancer Study (MDCS), Malmö Preventive Project (MPP), and Rotterdam Study (RS).
