# Supplementary material for: Discovery of Genetic Variation on Chromosome 5q22 Associated with Mortality in Heart Failure
Source: PLoS Genet. 2016 May 5;12(5):e1006034. doi: 10.1371/journal.pgen.1006034 (PMC4858216; doi:10.1371/journal.pgen.1006034)
Supplement: S6 Table — (DOCX) [file pgen.1006034.s014.docx]

**S6 Table. In silico studies of associations of rs9885413 with cardiac structure and function**

|  | ***P*-value** |
| --- | --- |
| **Echocardiography** |  |
| Systolic dysfunction | 0.67 |
| Fractional shortening | 0.46 |
| Left ventricular mass | 0.96 |
| Left ventricular wall thickness | 0.72 |
| Left ventricular internal diastolic dimension | 0.34 |
| Left atrial size | 0.58 |
| **Electrocardiography** |  |
| QRS interval duration | 0.37 |
| QT interval duration | 0.23 |
| **Natriuretic peptides** |  |
| Atrial natriuretic peptide | 0.77 |
| B-type natriuretic peptide | 0.31 |
| **Heart failure, incident** | 0.30 |
| **Sudden cardiac death** | 0.77 |
