# Supplementary material for: Discovery of Genetic Variation on Chromosome 5q22 Associated with Mortality in Heart Failure
Source: PLoS Genet. 2016 May 5;12(5):e1006034. doi: 10.1371/journal.pgen.1006034 (PMC4858216; doi:10.1371/journal.pgen.1006034)
Supplement: S7 Table — Enhancer regions in the 129 tissues from the ROADMAP Epigenomics Project, as determined from the ChromHMM algorithm from patterns of monomethylation of the fourth residue (lysine) of histone H3 (H3K4Me1). Enhancers overlapping the lead SNP on chromosome 5q22 (rs9885413) and strongly correlated SNPs (r2 > 0.8), with enhancers overlapping the lead SNP shown in bold. (DOCX) [file pgen.1006034.s015.docx]

**S7 Table. Enhancer annotations in the ROADMAP Epigenomics Project.**

| **Tissue** | **Chr** | **Start pos** | **End pos** | **SNP** | **ROADMAP tissue** |
| --- | --- | --- | --- | --- | --- |
| **E021** | **5** | **110176000** | **110176200** | **rs9885413** | **iPS DF 6.9 Cell Line** |
| **E058** | **5** | **110176000** | **110177200** | **rs9885413** | **Foreskin Keratinocyte Primary Cells skin03** |
| **E063** | **5** | **110176000** | **110176600** | **rs9885413** | **Adipose Nuclei** |
| **E078** | **5** | **110176000** | **110176400** | **rs9885413** | **Duodenum Smooth Muscle** |
| **E079** | **5** | **110176000** | **110176600** | **rs9885413** | **Esophagus** |
| **E092** | **5** | **110175800** | **110176600** | **rs9885413** | **Fetal Stomach** |
| **E111** | **5** | **110174600** | **110177000** | **rs9885413** | **Stomach Smooth Muscle** |
| **E127** | **5** | **110175800** | **110177400** | **rs9885413** | **NHEK-Epidermal Keratinocyte Primary Cells** |
| E076 | 5 | 110172400 | 110173600 | rs12658193 | Colon Smooth Muscle |
| E078 | 5 | 110172200 | 110172800 | rs12658193 | Duodenum Smooth Muscle |
| E092 | 5 | 110172000 | 110174400 | rs12658193 | Fetal Stomach |
| E103 | 5 | 110172000 | 110172600 | rs12658193 | Rectal Smooth Muscle |
| E115 | 5 | 110170000 | 110171000 | rs10069077 | Dnd41 TCell Leukemia Cell Line |
| E023 | 5 | 110219800 | 110220600 | rs12110223 | Mesenchymal Stem Cell Derived Adipocyte Cultured Cells |
| E025 | 5 | 110219200 | 110220600 | rs12110223 | Adipose Derived Mesenchymal Stem Cell Cultured  Cells |
| E052 | 5 | 110219000 | 110220600 | rs12110223 | Muscle Satellite Cultured Cells |
| E023 | 5 | 110232200 | 110233000 | rs1423154 | Mesenchymal Stem Cell Derived Adipocyte Cultured Cells |
| E025 | 5 | 110232200 | 110233200 | rs1423154 | Adipose Derived Mesenchymal Stem Cell Cultured  Cells |
| E052 | 5 | 110231800 | 110233600 | rs1423154 | Muscle Satellite Cultured Cells |
| E125 | 5 | 110232200 | 110233000 | rs1423154 | NH-A Astrocytes Primary Cells |
| E126 | 5 | 110232200 | 110233400 | rs1423154 | NHDF-Ad Adult Dermal Fibroblast Primary Cells |
| E128 | 5 | 110232200 | 110233600 | rs1423154 | NHLF Lung Fibroblast Primary Cells |
| E003 | 5 | 110235400 | 110235600 | rs7703957 | H1 Cell Line |
| E010 | 5 | 110235200 | 110235600 | rs7703957 | H9 Derived Neuron Cultured Cells |
| E016 | 5 | 110234800 | 110236200 | rs7703957 | HUES64 Cell Line |
| E017 | 5 | 110235200 | 110235600 | rs7703957 | IMR90 fetal lung fibroblasts Cell Line |
| E018 | 5 | 110234800 | 110237800 | rs7703957 | iPS-15b Cell Line |
| E019 | 5 | 110235400 | 110237600 | rs7703957 | iPS-18 Cell Line |
| E023 | 5 | 110235400 | 110237200 | rs7703957 | Mesenchymal Stem Cell Derived Adipocyte  Cultured Cells |
| E024 | 5 | 110235200 | 110236600 | rs7703957 | ES-UCSF4 Cell Line |
| E025 | 5 | 110235200 | 110236000 | rs7703957 | Adipose Derived Mesenchymal Stem Cell  Cultured Cells |
| E026 | 5 | 110235400 | 110237600 | rs7703957 | Bone Marrow Derived Cultured Mesenchymal Stem  Cells |
| E028 | 5 | 110234800 | 110236600 | rs7703957 | Breast variant Human Mammary Epithelial Cells (vHMEC) |
| E055 | 5 | 110232800 | 110235600 | rs7703957 | Foreskin Fibroblast Primary Cells skin01 |
| E057 | 5 | 110234800 | 110236400 | rs7703957 | Foreskin Keratinocyte Primary Cells skin02 |
| E058 | 5 | 110234800 | 110236200 | rs7703957 | Foreskin Keratinocyte Primary Cells skin03 |
| E116 | 5 | 110235200 | 110237000 | rs7703957 | GM12878 Lymphoblastoid Cell Line |
| E119 | 5 | 110235000 | 110237800 | rs7703957 | HMEC Mammary Epithelial Primary Cells |
| E126 | 5 | 110235400 | 110236800 | rs7703957 | NHDF-Ad Adult Dermal Fibroblast Primary Cells |
| E127 | 5 | 110235000 | 110237400 | rs7703957 | NHEK-Epidermal Keratinocyte Primary Cells |
| E128 | 5 | 110235400 | 110238400 | rs7703957 | NHLF Lung Fibroblast Primary Cells |
| E129 | 5 | 110235200 | 110237800 | rs7703957 | Osteoblast Primary Cells |
| E001 | 5 | 110235600 | 110238000 | rs1862532 | ES-I3 Cell Line |
| E002 | 5 | 110235600 | 110236000 | rs1862532 | ES-WA7 Cell Line |
| E014 | 5 | 110235800 | 110238800 | rs1862532 | HUES48 Cell Line |
| E015 | 5 | 110235600 | 110237000 | rs1862532 | HUES6 Cell Line |
| E016 | 5 | 110234800 | 110236200 | rs1862532 | HUES64 Cell Line |
| E018 | 5 | 110234800 | 110237800 | rs1862532 | iPS-15b Cell Line |
| E019 | 5 | 110235400 | 110237600 | rs1862532 | iPS-18 Cell Line |
| E020 | 5 | 110235800 | 110237000 | rs1862532 | iPS-20b Cell Line |
| E023 | 5 | 110235400 | 110237200 | rs1862532 | Mesenchymal Stem Cell Derived Adipocyte Cultured Cells |
| E024 | 5 | 110235200 | 110236600 | rs1862532 | ES-UCSF4 Cell Line |
| E025 | 5 | 110235200 | 110236000 | rs1862532 | Adipose Derived Mesenchymal Stem Cell Cultured  Cells |
| E026 | 5 | 110235400 | 110237600 | rs1862532 | Bone Marrow Derived Cultured Mesenchymal Stem  Cells |
| E028 | 5 | 110234800 | 110236600 | rs1862532 | Breast variant Human Mammary Epithelial Cells (vHMEC) |
| E057 | 5 | 110234800 | 110236400 | rs1862532 | Foreskin Keratinocyte Primary Cells skin02 |
| E058 | 5 | 110234800 | 110236200 | rs1862532 | Foreskin Keratinocyte Primary Cells skin03 |
| E116 | 5 | 110235200 | 110237000 | rs1862532 | GM12878 Lymphoblastoid Cell Line |
| E119 | 5 | 110235000 | 110237800 | rs1862532 | HMEC Mammary Epithelial Primary Cells |
| E120 | 5 | 110235800 | 110237200 | rs1862532 | HSMM Skeletal Muscle Myoblasts Cell Line |
| E125 | 5 | 110235800 | 110236600 | rs1862532 | NH-A Astrocytes Primary Cells |
| E126 | 5 | 110235400 | 110236800 | rs1862532 | NHDF-Ad Adult Dermal Fibroblast Primary Cells |
| E127 | 5 | 110235000 | 110237400 | rs1862532 | NHEK-Epidermal Keratinocyte Primary Cells |
| E128 | 5 | 110235400 | 110238400 | rs1862532 | NHLF Lung Fibroblast Primary Cells |
| E129 | 5 | 110235200 | 110237800 | rs1862532 | Osteoblast Primary Cells |
| E001 | 5 | 110235600 | 110238000 | rs1862530 | ES-I3 Cell Line |
| E014 | 5 | 110235800 | 110238800 | rs1862530 | HUES48 Cell Line |
| E015 | 5 | 110235600 | 110237000 | rs1862530 | HUES6 Cell Line |
| E016 | 5 | 110234800 | 110236200 | rs1862530 | HUES64 Cell Line |
| E018 | 5 | 110234800 | 110237800 | rs1862530 | iPS-15b Cell Line |
| E019 | 5 | 110235400 | 110237600 | rs1862530 | iPS-18 Cell Line |
| E020 | 5 | 110235800 | 110237000 | rs1862530 | iPS-20b Cell Line |
| E023 | 5 | 110235400 | 110237200 | rs1862530 | Mesenchymal Stem Cell Derived Adipocyte Cultured Cells |
| E024 | 5 | 110235200 | 110236600 | rs1862530 | ES-UCSF4 Cell Line |
| E026 | 5 | 110235400 | 110237600 | rs1862530 | Bone Marrow Derived Cultured Mesenchymal Stem  Cells |
| E028 | 5 | 110234800 | 110236600 | rs1862530 | Breast variant Human Mammary Epithelial Cells (vHMEC) |
| E049 | 5 | 110236000 | 110236800 | rs1862530 | Mesenchymal Stem Cell Derived Chondrocyte  Cultured Cells |
| E057 | 5 | 110234800 | 110236400 | rs1862530 | Foreskin Keratinocyte Primary Cells skin02 |
| E058 | 5 | 110234800 | 110236200 | rs1862530 | Foreskin Keratinocyte Primary Cells skin03 |
| E116 | 5 | 110235200 | 110237000 | rs1862530 | GM12878 Lymphoblastoid Cell Line |
| E119 | 5 | 110235000 | 110237800 | rs1862530 | HMEC Mammary Epithelial Primary Cells |
| E120 | 5 | 110235800 | 110237200 | rs1862530 | HSMM Skeletal Muscle Myoblasts Cell Line |
| E125 | 5 | 110235800 | 110236600 | rs1862530 | NH-A Astrocytes Primary Cells |
| E126 | 5 | 110235400 | 110236800 | rs1862530 | NHDF-Ad Adult Dermal Fibroblast Primary Cells |
| E127 | 5 | 110235000 | 110237400 | rs1862530 | NHEK-Epidermal Keratinocyte Primary Cells |
| E128 | 5 | 110235400 | 110238400 | rs1862530 | NHLF Lung Fibroblast Primary Cells |
| E129 | 5 | 110235200 | 110237800 | rs1862530 | Osteoblast Primary Cells |
| E001 | 5 | 110235600 | 110238000 | rs17132484 | ES-I3 Cell Line |
| E014 | 5 | 110235800 | 110238800 | rs17132484 | HUES48 Cell Line |
| E018 | 5 | 110234800 | 110237800 | rs17132484 | iPS-15b Cell Line |
| E019 | 5 | 110235400 | 110237600 | rs17132484 | iPS-18 Cell Line |
| E025 | 5 | 110236200 | 110237800 | rs17132484 | Adipose Derived Mesenchymal Stem Cell Cultured  Cells |
| E026 | 5 | 110235400 | 110237600 | rs17132484 | Bone Marrow Derived Cultured Mesenchymal Stem  Cells |
| E028 | 5 | 110237000 | 110237600 | rs17132484 | Breast variant Human Mammary Epithelial Cells (vHMEC) |
| E052 | 5 | 110236400 | 110238600 | rs17132484 | Muscle Satellite Cultured Cells |
| E058 | 5 | 110237200 | 110237400 | rs17132484 | Foreskin Keratinocyte Primary Cells skin03 |
| E119 | 5 | 110235000 | 110237800 | rs17132484 | HMEC Mammary Epithelial Primary Cells |
| E125 | 5 | 110237000 | 110238600 | rs17132484 | NH-A Astrocytes Primary Cells |
| E127 | 5 | 110235000 | 110237400 | rs17132484 | NHEK-Epidermal Keratinocyte Primary Cells |
| E128 | 5 | 110235400 | 110238400 | rs17132484 | NHLF Lung Fibroblast Primary Cells |
| E129 | 5 | 110235200 | 110237800 | rs17132484 | Osteoblast Primary Cells |

Enhancer regions in the 129 tissues from the ROADMAP Epigenomics Project, as determined from the ChromHMM algorithm from patterns of monomethylation of the fourth residue (lysine) of histone H3 (H3K4Me1). Enhancers overlapping the lead SNP on chromosome 5q22 (rs9885413) and strongly correlated SNPs (r^2^ > 0.8), with enhancers overlapping the lead SNP shown in bold.
