# Supplementary material for: Discovery of Genetic Variation on Chromosome 5q22 Associated with Mortality in Heart Failure
Source: PLoS Genet. 2016 May 5;12(5):e1006034. doi: 10.1371/journal.pgen.1006034 (PMC4858216; doi:10.1371/journal.pgen.1006034)
Supplement: S8 Table — * The two alleles of the SNP rs9885413. (DOCX) [file pgen.1006034.s016.docx]

**S8 Table. Location of rs9885413 in the predicted NHLH1 binding site**

T T **C/A*** A T T C T C A G C T G T T G C C C A A

* The two alleles of the SNP rs9885413.
