# Supplementary material for: Discovery of Genetic Variation on Chromosome 5q22 Associated with Mortality in Heart Failure
Source: PLoS Genet. 2016 May 5;12(5):e1006034. doi: 10.1371/journal.pgen.1006034 (PMC4858216; doi:10.1371/journal.pgen.1006034)
Supplement: S9 Table — Association of rs9885413 with expression of the five genes at the chromosomal locus in 13 tissues from the Gene-Tissue Expression Project with data available in > 60 samples. Empirical P-values were computed using a permutation approach as described in documentation for the GTEx project (http://www.gtexportal.org). N/A, no results available in GTEx. Effect direction is presented for associations with P ≤ 0.1. (DOCX) [file pgen.1006034.s017.docx]

**S9 Table. Association of rs9885413 with gene expression in the GTEx Project**

| **SNP** | **Transcript** | **GTEX tissue** | **Direction** | ***P* value** |
| --- | --- | --- | --- | --- |
| rs9885413 | TMEM232 | Adipose Subcutaneous | + | 0.04 |
| rs9885413 | TMEM232 | Aorta | + | 0.06 |
| rs9885413 | TMEM232 | Artery Tibial | + | 0.03 |
| rs9885413 | TMEM232 | Blood |  | N/A |
| rs9885413 | TMEM232 | Esophagus Mucosa | + | 0.01 |
| rs9885413 | TMEM232 | Esophagus Muscularis | + | 0.02 |
| rs9885413 | TMEM232 | Heart left ventricle |  | N/A |
| rs9885413 | TMEM232 | Lung |  | 0.9 |
| rs9885413 | TMEM232 | Muscle skeletal |  | N/A |
| rs9885413 | TMEM232 | Nerve Tibial | + | 0.01 |
| rs9885413 | TMEM232 | Skin sun exposed | + | 0.1 |
| rs9885413 | TMEM232 | Stomach |  | 0.9 |
| rs9885413 | TMEM232 | Thyroid | + | 0.05 |
| rs9885413 | SLC25A46 | Adipose Subcutaneous |  | 0.2 |
| rs9885413 | SLC25A46 | Aorta |  | 0.9 |
| rs9885413 | SLC25A46 | Artery Tibial |  | 0.3 |
| rs9885413 | SLC25A46 | Blood |  | 0.8 |
| rs9885413 | SLC25A46 | Esophagus Mucosa | - | 0.008 |
| rs9885413 | SLC25A46 | Esophagus Muscularis |  | 0.9 |
| rs9885413 | SLC25A46 | Heart left ventricle |  | 1.0 |
| rs9885413 | SLC25A46 | Lung |  | 0.2 |
| rs9885413 | SLC25A46 | Muscle skeletal |  | 1.0 |
| rs9885413 | SLC25A46 | Nerve Tibial |  | 0.6 |
| rs9885413 | SLC25A46 | Skin sun exposed | + | 0.005 |
| rs9885413 | SLC25A46 | Stomach |  | 0.6 |
| rs9885413 | SLC25A46 | Thyroid |  | 0.8 |
| rs9885413 | TSLP | Adipose Subcutaneous | - | 0.09 |
| rs9885413 | TSLP | Aorta |  | 1.0 |
| rs9885413 | TSLP | Artery Tibial |  | 0.8 |
| rs9885413 | TSLP | Blood |  | N/A |
| rs9885413 | TSLP | Esophagus Mucosa |  | 0.9 |
| rs9885413 | TSLP | Esophagus Muscularis |  | 0.5 |
| rs9885413 | TSLP | Heart left ventricle |  | 0.4 |
| rs9885413 | TSLP | Lung |  | 0.7 |
| rs9885413 | TSLP | Muscle skeletal |  | 0.5 |
| rs9885413 | TSLP | Nerve Tibial |  | 0.8 |
| rs9885413 | TSLP | Skin sun exposed |  | 0.7 |
| rs9885413 | TSLP | Stomach |  | 0.8 |
| rs9885413 | TSLP | Thyroid |  | 0.5 |
| rs9885413 | WDR36 | Adipose Subcutaneous |  | 0.6 |
| rs9885413 | WDR36 | Aorta |  | 0.8 |
| rs9885413 | WDR36 | Artery Tibial |  | 0.8 |
| rs9885413 | WDR36 | Blood |  | 0.5 |
| rs9885413 | WDR36 | Esophagus Mucosa |  | 0.2 |
| rs9885413 | WDR36 | Esophagus Muscularis |  | 0.4 |
| rs9885413 | WDR36 | Heart left ventricle |  | 0.9 |
| rs9885413 | WDR36 | Lung | + | 0.06 |
| rs9885413 | WDR36 | Muscle skeletal |  | 0.5 |
| rs9885413 | WDR36 | Nerve Tibial | + | 0.1 |
| rs9885413 | WDR36 | Skin sun exposed |  | 0.9 |
| rs9885413 | WDR36 | Stomach |  | 0.6 |
| rs9885413 | WDR36 | Thyroid |  | 0.8 |
| rs9885413 | CAMK4 | Adipose Subcutaneous | - | 0.1 |
| rs9885413 | CAMK4 | Aorta |  | 0.4 |
| rs9885413 | CAMK4 | Artery Tibial |  | 1.0 |
| rs9885413 | CAMK4 | Blood |  | 0.8 |
| rs9885413 | CAMK4 | Esophagus Mucosa |  | 0.2 |
| rs9885413 | CAMK4 | Esophagus Muscularis | - | 0.03 |
| rs9885413 | CAMK4 | Heart left ventricle |  | N/A |
| rs9885413 | CAMK4 | Lung |  | 0.2 |
| rs9885413 | CAMK4 | Muscle skeletal |  | N/A |
| rs9885413 | CAMK4 | Nerve Tibial | + | 0.02 |
| rs9885413 | CAMK4 | Skin sun exposed | + | 0.02 |
| rs9885413 | CAMK4 | Stomach | - | 0.1 |
| rs9885413 | CAMK4 | Thyroid | + | 0.1 |

Association of rs9885413 with expression of the five genes at the locus on chromosome 5q22 in 13 tissues from the Gene-Tissue Expression Project with data available in > 60 samples. Empirical *P*-values were computed using a permutation approach as described in documentation for the GTEx project (http://www.gtexportal.org). For these analyses, p-values below 8.5x10^-4^ were considered significant, based on Bonferroni-correction for 59 expressed genes. N/A, no results available in GTEx. Effect direction is presented for associations with *P* ≤ 0.1.
