# Supplementary material for: Discovery of Genetic Variation on Chromosome 5q22 Associated with Mortality in Heart Failure
Source: PLoS Genet. 2016 May 5;12(5):e1006034. doi: 10.1371/journal.pgen.1006034 (PMC4858216; doi:10.1371/journal.pgen.1006034)
Supplement: S10 Table — Association of rs9885413 with expression of the five genes at the locus on chromosome 5q22 in 247 human heart samples from 116 patients with advanced heart failure (HF) and 131 unused transplant donor hearts (controls). Transcript expression levels were considered significantly higher than background noise if expression values from robust multiarray analysis in at least 10% of either cases or controls exceeded of the 80% quantile of expression of genes on the Y-chromosome in female hearts (5.24). Positive direction indicates higher expression with the risk (minor) allele of rs9885413. (DOCX) [file pgen.1006034.s018.docx]

**S10 Table. Association of rs9885413 with gene expression in human heart**

| **Transcript** | **HF mean** | **Control mean** | **Direction** | ***P* value** |
| --- | --- | --- | --- | --- |
| TMEM232 | 5.30 | 4.85 | + | 2.2x10^-6^ |
| SLC25A46 | 8.70 | 8.84 |  | 0.36 |
| TSLP | 3.27 | 3.31 |  | N/A |
| WDR36 | 6.74 | 6.79 |  | 0.27 |
| CAMK4 | 3.88 | 3.97 |  | N/A |

Association of rs9885413 with expression of the five genes at the locus on chromosome 5q22 in 247 human heart samples from 116 patients with advanced heart failure (HF) and 131 unused transplant donor hearts (controls). Transcript expression levels were considered significantly higher than background noise if expression values from robust multiarray analysis in at least 10% of either cases or controls exceeded of the 80% quantile of expression of genes on the Y-chromosome in female hearts (5.24). Positive direction indicates higher expression with the risk (minor) allele of rs9885413.
